# Highly regioselective oxidation of C–H bonds in water using hydrogen peroxide by a cytochrome P450 mimicking iron complex†

**DOI:** 10.1039/d3sc03495j

**Published:** 2023-09-08

**Authors:** Sandipan Jana, Puja De, Chinmay Dey, Somdatta Ghosh Dey, Abhishek Dey, Sayam Sen Gupta

**Affiliations:** a Department of Chemical Sciences, Indian Institute of Science Education and Research Mohanpur 741246 Kolkata India sayam.sengupta@iiserkol.ac.in; b School of Chemical Sciences, Indian Association for the Cultivation of Science Kolkata West Bengal 700032 India icad@iacs.res.in icsgd@iacs.res.in

## Abstract

Cytochrome P450, one of nature's oxidative workhorses, catalyzes the oxidation of C–H bonds in complex biological settings. Extensive research has been conducted over the past five decades to develop a fully functional mimic that activates O_2_ or H_2_O_2_ in water to oxidize strong C–H bonds. We report the first example of a synthetic iron complex that functionally mimics cytochrome P450 in 100% water using H_2_O_2_ as the oxidant. This iron complex, in which one methyl group is replaced with a phenyl group in either wing of the macrocycle, oxidized unactivated C–H bonds in small organic molecules with very high selectivity in water (pH 8.5). Several substrates (34 examples) that contained arenes, heteroaromatics, and polar functional groups were oxidized with predictable selectivity and stereoretention with moderate to high yields (50–90%), low catalyst loadings (1–4 mol%) and a small excess of H_2_O_2_ (2–3 equiv.) in water. Mechanistic studies indicated the oxoiron(v) to be the active intermediate in water and displayed unprecedented selectivity towards 3° C–H bonds. Under single-turnover conditions, the reactivity of this oxoiron(v) intermediate in water was found to be around 300 fold higher than that in CH_3_CN, thus implying the role water plays in enzymatic systems.

## Abstract 2

Development of a molecular iron complex, which *via* oxoiron(v) formation, catalyzes highly efficient and regioselective oxidation of C–H bonds in water using hydrogen peroxide, and thereby acts as a functional model of cytochrome P450.

## Graphical abstract

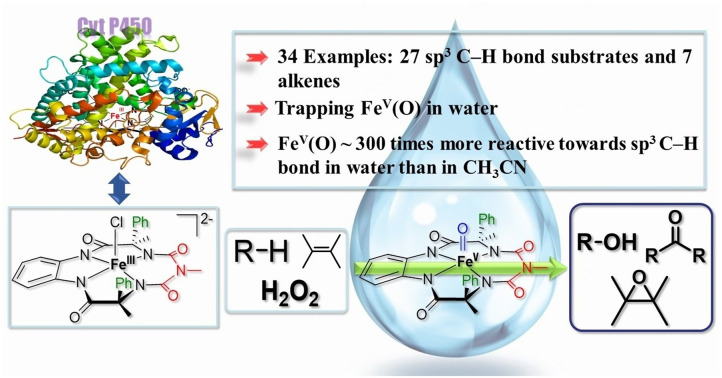

## Introduction

Nature utilizes earth-abundant iron-based enzymes, such as cytochrome P450, for the highly selective oxidation of C–H and C=C bonds, under mild physiological conditions using molecular oxygen (O_2_) or hydrogen peroxide (H_2_O_2_) as the terminal oxidant.^1^ The quest for synthesizing biologically or industrially significant molecules *via* C–H bond functionalization has driven researchers worldwide to develop synthetic model complexes of these oxygenases and other enzymes,^2*a*,*b*^ which can also prove essential in deciphering the mechanism of action of natural enzymes. Synthetic models^2*c*^ of heme enzymes (or Cyt P450), which are excellent structural models due to their ability to activate H_2_O_2_ to form the oxoiron(iv) porphyrin π–cation radical,^2*d*^ showed limited ability in catalytic hydroxylation and were not scalable. In addition, they mostly function in organic solvents.

**Fig. 1:**
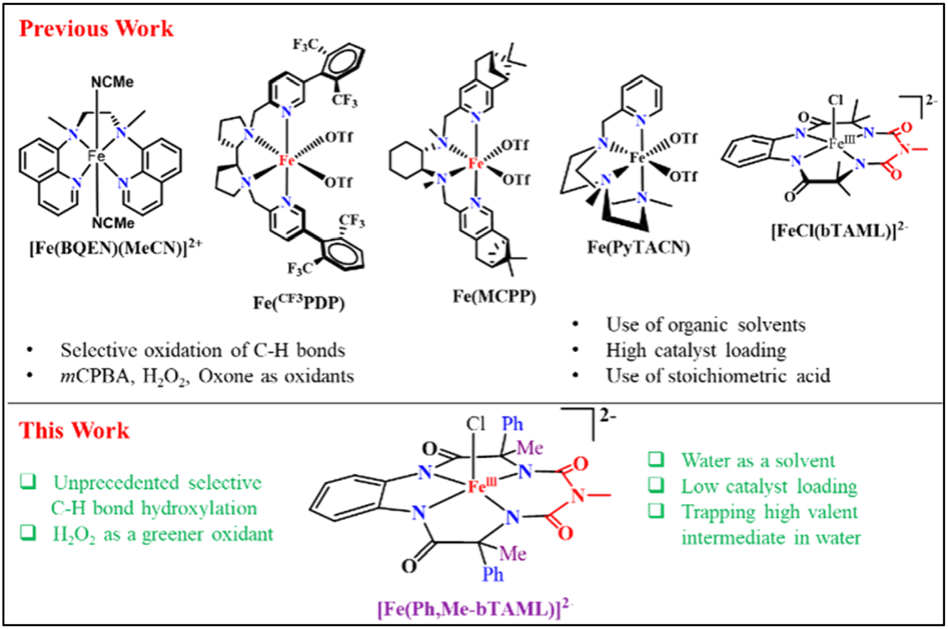
Comparison of various Fe-complexes used in C–H oxidation.

The major breakthrough in selective metal-catalyzed C–H oxidation arrived in the form of structural Rieske dioxygenase model complexes, using H_2_O_2_ as the oxidant and an acid source. Starting from less bulky and less selective Fe-TPA^3*a*^ and Fe-MEP complexes^3*b*^ to pyrrolidine containing pyridine-N_4_ ligand-based Fe-PDP,^3*c*,*d*^ Fe-CF_3_PDP^3*e*^ and related sterically bulky ligated catalysts (Fe-MCPP, Fe-^tips^MCP, Fe-^tips^PDP, and Fe-PyTACN),^4^ they performed highly regioselective oxidation of C–H bonds in a scalable manner. Meanwhile, our group has developed a robust iron complex based on a biuret-modified tetra-amido macrocyclic ligand (Fe-bTAML)^5*a*,*b*^ which, unlike Fe-N_4_ systems, can perform selective hydroxylation of tertiary C–H bonds containing arenes and heteroarene compounds using *m*CPBA as the terminal oxidant.^5*c*,*d*^ It is worth mentioning here that none of the synthetically useful biomimetic non-heme model complexes utilizes the green solvent “water” as the predominant solvent under non-acidic conditions (acetonitrile had been operational in the majority of the reported literature). Reports on the oxidation of C–H or C=C bonds in water are quite rare and limited to small substrate scopes, poor yields, and poor selectivities. These include the use of expensive second row transition metals^6^ like ruthenium or rhodium, as well as earth-abundant metals^7^ like iron and manganese as oxidation catalysts. On the other hand, oxidation of methane to methanol using H_2_O_2_ in water has been achieved by iron based catalysts^8*a*^ such as the μ-nitrido diironphthalocyanine complex^8*b*^ and *N*-heterocyclic carbene-ligated iron(ii) complex.^8*c*^ In 2020, a new heterogenous construct involving an immobilized iron porphyrin complex on a self-assembled monolayer on Au electrodes was implemented for the oxidation of organic substrates in an aqueous solution using H_2_O_2_.^8*d*,*e*^ In summary, iron complexes that utilize H_2_O_2_ as an oxidant in 100% water under homogeneous conditions to catalyze selective and scalable oxidation of unactivated C–H bonds are unknown (Fig. 1).

Fe-TAMLs are historically known to activate H_2_O_2_ for oxidation in water under near-neutral conditions with properties comparable to those of native peroxidase enzymes,^9*a*^ namely, for the degradation of dyes, drugs, and pesticides which have mainly very weak C–H and O–H bonds.^9*a*–*d*^ The main species formed in this case, however, were Fe^III^ and Fe^IV^ dimers in near-neutral pH and Fe^IV^(O) in highly basic pH respectively, which were unable to oxidize strong sp^3^ C–H bonds. The fifth-generation Fe-bTAMLs reported so far also form non-catalytic μ-Fe^IV^-oxo dimers in pH < 10 in the presence of H_2_O_2_. However, in CH_3_CN and CH_3_CN : H_2_O mixtures, Fe-bTAML activates *m*CPBA or NaOCl to generate the oxoiron(v) intermediate which is capable of oxidation of strong C–H bonds (<99 kcal mol^−1^), both under single-turnover and catalytic conditions.^5*b*–*d*^ However, no reaction occurred in 100% water.

In TAMLs and bTAMLs reported so far, high-valent reactive oxoiron(v) species, except in one case,^10^ cannot be generated in 100% water or 100% aq. buffer solutions chemically, photochemically, or electrochemically (the one reported is not reactive toward C–H bonds). Generation of oxoiron(v) is critical for catalytic hydroxylation reactions. Hence activation of H_2_O_2_ to catalytically hydroxylate C–H bonds *via* the formation of an oxoiron(v) intermediate has been an unrealized dream. Such systems would be the first functional models of the Cyt P450 that hydroxylates C–H bonds using H_2_O_2_ as the terminal oxidant.

In this paper, we report a new modified Fe-bTAML complex that can generate an oxoiron(v) intermediate in 100% water that is competent to oxidize strong C–H bonds. The reactivity of this intermediate was a few hundred fold higher than the reactivity in CH_3_CN. We also show, for the first time, that this newly modified bTAML can activate hydrogen peroxide in water and oxidize a wide variety of substrates containing 3° C–H bonds (including natural products) and substrates containing C=C bonds in a selective and scalable manner. Notably, the selectivity and reactivity in water far exceed those observed in CH_3_CN – which helps us understand how protic solvents like water are critical to such reactivity.

## Results and discussion

### Synthesis and characterization of “picket-fence” Fe-bTAML

**Scheme 1:**
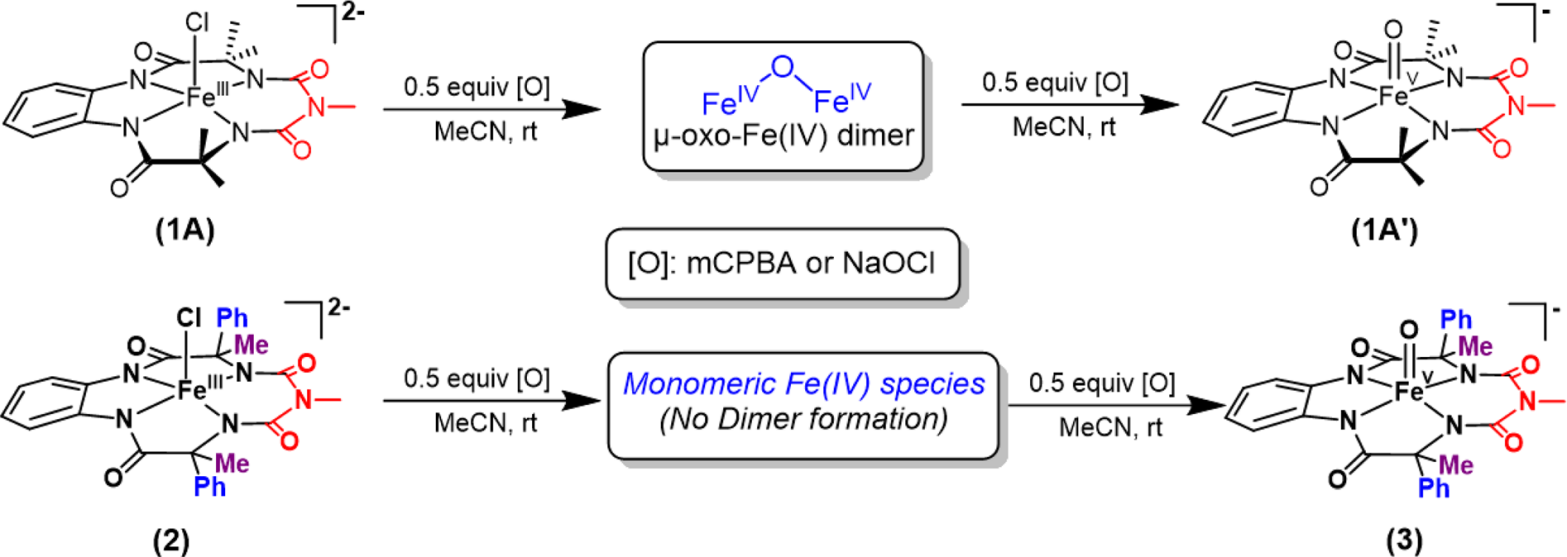
Formation of oxoiron(v) species (1′ and 3) *via* two-electron oxidation of 1 and 2 respectively in acetonitrile at room temperature.

**Fig. 2:**
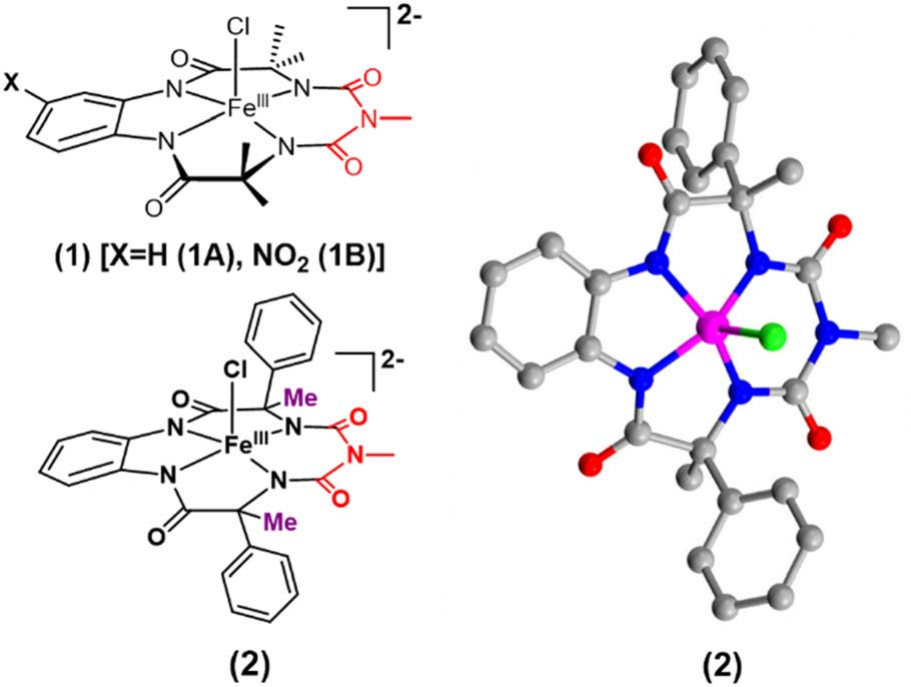
Left top: previously reported [FeCl(bTAML)]^2−^ complexes (1) relevant to this work; left bottom: (Et_4_N)_2_[Fe(Cl)(Ph,Me-bTAML)] complex (2); right: crystal structure of complex 2.

We report the synthesis of a Fe-bTAML complex (Et_4_N)_2_[Fe-(Cl)(Ph,Me-bTAML)], 2, where a phenyl group replaces a methyl group on either side of the biuret moiety (Scheme 1); thereby creating a steric bulk akin to a “picket fence”, in close proximity to the iron center. (Et_4_N)_2_[Fe(Cl)(Ph,Me-bTAML)] (2) was synthesized following a new methodology (9 steps) wherein (dl)-2-phenylpropanoic acid was chosen as the starting material. Before metalation, the macrocyclic ligand was crystallized (Fig. S17†) and found to be racemic and optically inactive (Fig. S18:† chiral HPLC, optical rotation measurement). It should be noted that during the macrocyclization reaction, the diastereomer of amine (R, S) does not cyclize since the resultant macrocycle would have both the Ph groups oriented in a sterically unfavourable *cis* conformation to each other. Likewise, 2 was also found to be achiral (optically inactive), and crystallizes in an orthorhombic crystal system (space group, *Pbca* (no. 61)) (Fig. 2: right and Fig. S23†). The complex was further characterized by ESI-MS, UV-Vis, EPR, and electrochemical studies. EPR of 2 exhibited *g*-values of 4.94, 4.29, and 2.02, suggesting an *S* = 3/2 spin state (Fig. S24†), similar to regular Fe(iii)-bTAML (1A).^5*a*^ The electrochemical properties of this complex in CH_3_CN revealed two reversible peaks with peak potentials at *E*_1/2_ = 0.227 V (Δ*E*_p_ = 64 mV) and *E*_1/2_ = 0.786 V (Δ*E*_p_ = 71 mV) *vs.* Ag/AgNO_3_ (0.01 M) (Fig. S27†). The peak-to-peak separation (Δ*E*_p_) indicated that both involve one electron redox process, which may be assigned to the Fe^IV^/Fe^III^ and Fe^V^/Fe^IV^ couples. In 100% water the *E*_1/2_[Fe^IV^/Fe^III^] value has increased up to 0.63 V (*vs.* Ag/AgCl, 3 M KCl), albeit with a loss of reversibility (Fig. S28†). This may be attributed to the formation of a six coordinated diaquo Fe^III^ complex upon the addition of water.^11^

**Fig. 3:**
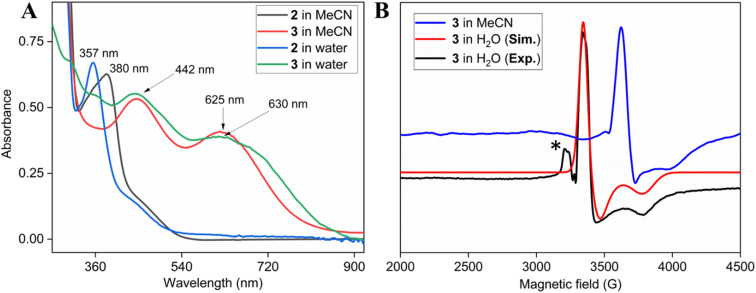
(A) Formation of 3 by addition of *m*CPBA to 0.12 mM 2 both in CH_3_CN and 100% water; (B) EPR spectra of 3 at 85 K (black: experiment, red: simulation; * mark: impurity coming from *m*CPBA), formed in both acetonitrile (blue spectrum) and 100% water medium (black).

Two-electron oxidation of 2 in acetonitrile by either *meta*-chloroperbenzoic acid (*m*CPBA) or aqueous sodium hypochlorite (NaOCl) led to the formation of oxoiron(v) species (3) whose UV spectral features (green colour, *λ*_max_ 442 nm and 625 nm) are reminiscent of 1A′ (Fig. 3A). 3 was well-characterized by UV-Vis (Fig. S29†), HRMS (Fig. S32†), EPR, and Mössbauer spectroscopies. The X-band EPR spectrum of 3 at 85 K revealed a rhombic *S* = ½ species with *g* = 2.00, 1.98, and 1.83 (*g*_ave_ 1.93) (Fig. 3B and S25†), consistent with oxoiron(v)-TAML^12^ and 1A′^5*b*^ respectively. The Mössbauer spectrum of ^57^Fe enriched 3 at 77 K showed characteristic *δ*_iso_ and Δ*E*_q_ of −0.58 cm^−1^ and 4.11 cm^−1^, respectively (Fig. S26†), consistent with the oxoiron(v) species (>60%). In addition to this, there was another species with *δ*_iso_ and Δ*E*_q_ of −0.2 cm^−1^ and 3 cm^−1^, respectively, which may correspond to a monomeric Fe^IV^ species (23%) forming under the same reaction conditions. These values are nearly identical to that of oxoiron(v) species of bTAML, 1A′.^5*b*^

Earlier studies have shown that the formation of the oxoiron(v) intermediate of Fe-bTAML (1A′) in CH_3_CN medium upon the addition of *m*CPBA or NaOCl occurs through the formation of the dimeric intermediate, μ-oxo-diiron(iv), [{(bTAML)Fe^IV^}_2_-μ-oxo]^2−^.^5*b*^ In fact, the addition of 0.5 equiv. of *m*CPBA to 1 led to the formation of the diamagnetic μ-oxo-diiron(iv)dimer *via* comproportionation of 1A and 1A′ (Scheme 1). The addition of another 0.5 equiv. of *m*CPBA converts the μ-oxo-diiron(iv)dimer to the oxoiron(v) 1A′ intermediate. However, unlike 1, complex 2 does not dimerize upon addition of 0.5 equiv. of *m*CPBA (Scheme 1). In this case, a new intermediate species with *λ*_max_ of 432 nm and 625 nm was observed. The UV-Vis spectral features were devoid of the broad peak between 850 and 1000 nm (Fig. S29†) which is characteristic of dimer formation. EPR (Fig. S30†) and paramagnetic NMR experiments ((expt) = 3.4) (Fig. S31†) (high magnetic moment value) indicate that this intermediate is paramagnetic and can be best explained as a mixture of Fe(iii) and Fe(v) or formation of a new monomeric Fe(iv) species (*S* = 1). In summary, the installation of the phenyl groups did result in preventing dimer formation as was hypothesized. The oxoiron(v) species (3) was reactive towards the hydroxylation of unactivated C–H bonds and the mechanism observed was similar to that reported for oxoiron(v) FebTAML 1A′. However, 3 was found to be around 300 times more reactive toward C–H bonds in comparison to 1A′.

### Formation of oxoiron(v) in 100% water and its reactivity towards C–H bonds

The formation and spectroscopic characterization of the oxoiron(v) intermediate in 100% water is extremely challenging. In 2016, Collins *et al.* reported a beheaded TAML activator that can stabilize oxoiron(v) species in pure water, although it was not shown to be reactive towards C–H or C=C bonds.^10^ Barring this report, quantitative oxoiron(v) formation was not achieved using any synthetic model complexes including Fe-TAML and bTAML systems in 100% water.

Addition of *m*CPBA to the parent (Et_4_N)_2_[Fe(Cl)(Ph,Me-bTAML)] complex 2 at 10 °C in >99.5% water (0.5% CH_3_CN) produced green-coloured species, whose UV-Vis showed spectral features at *λ*_max_ of 442 nm and 630 nm that are similar to that obtained in CH_3_CN (Fig. 2A and S37†). The X-band EPR spectrum at 85 K showed a rhombic *S* = ½ species with *g* = 1.985, 1.950, 1.755 (Fig. 3B). HRMS examination of the green-coloured species in 100% water revealed one prominent ion at a mass-to-charge ratio of 553.1165, which corresponds to the oxoiron(v) species 3 (calculated *m*/*z* 553.1064 of 3) (Fig. S38†). The introduction of H_2_O^18^ as the solvent medium during HRMS showed 70% incorporation of labeled oxygen (*m*/*z* 555.0997; corresponding to the ^18^O-incorporated 3) (Fig. S39†).

**Scheme 2:**
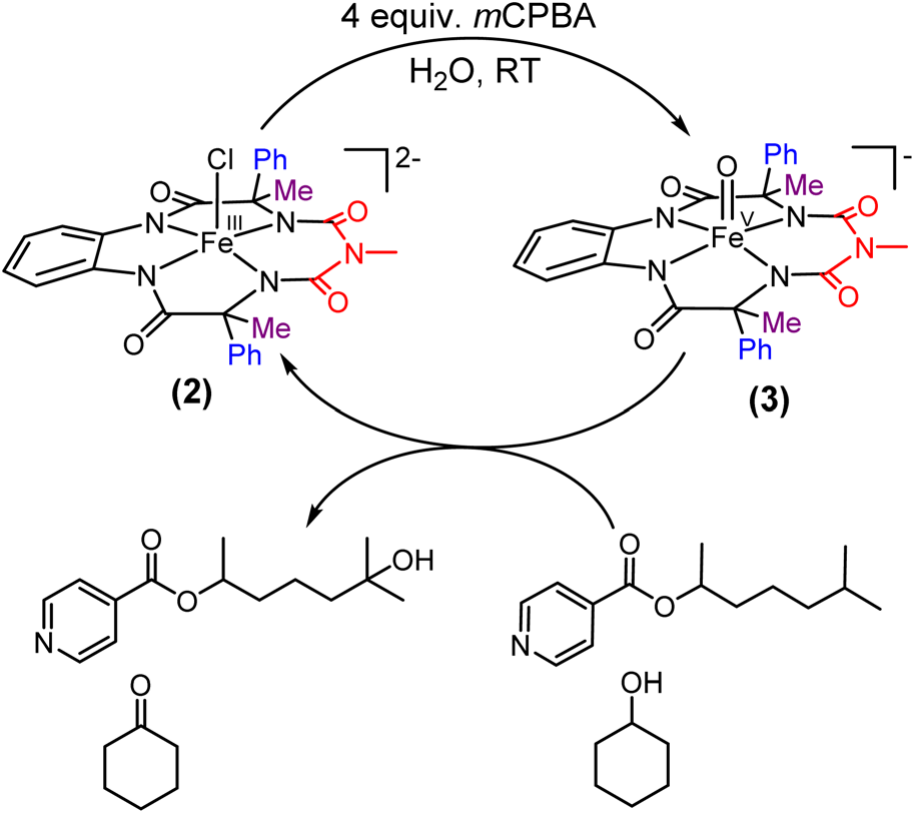
Formation of the oxoiron(v) species in 100% water and its reactivity towards strong C–H bonds.

The self-decay rate of 3 in water at 10 °C, measured by monitoring the decrease in the characteristic 630 nm band of 3 (0.1218 s^−1^), was ∼200 times faster (Fig. S40†) than in CH_3_CN (6.0531 × 10^−4^ s^−1^) medium and also ∼2700 times faster than oxoiron(v) of regular bTAML (1A′). However, this low self-stability in water does not compromise its reactivity toward C–H bonds (Fig. S41†). In fact, 3 was found to be highly reactive toward the oxidation of strong C–H bonds. In water, under single turnover conditions, 0.5 mM 2 selectively oxidized 250 mM toluene leading to the exclusive formation of benzaldehyde in 35% yield (the yield is greater than the theoretical yield of 25% since a slight excess of *m*CPBA is present in the solution). Similar selectivity, conversion, and formation of the corresponding 3° hydroxylated product were also obtained for *cis*-/*trans*-dimethylcyclohexane and decahydronaphthalene. Especially remarkable is the 100% stereo- and regioselectivity (3°) observed for the *trans*-hydroxylated product during the oxidation of *trans*-isomers of dimethylcyclohexane and decahydronaphthalene. Earlier studies showed that oxidation of 3° C–H bonds in these substrates is difficult because 1,3-diaxial interaction and oxidation are preferred towards the 2° C–H bonds. For example, in oxidation using Fe-PDP complexes,^3*e*^ the selectivity towards 2° C–H bonds over 3° C–H bonds in *trans*-dimethylcyclohexane is approximately 9-fold. Similarly, in the case of 1B catalyzed oxidation in CH_3_CN,^5*c*^ 24% and 62% of the 2° products were obtained for *trans*-isomers of dimethylcyclohexane and decahydronaphthalene respectively, along with 3° products. Based on these literature reports, the complete stereo retention observed in the hydroxylated product for these *trans* isomers with 2 in water is truly remarkable. For the determination of reaction rates in 100% water, 6-methylheptan-2-yl isonicotinate (alkane substrate, BDE >92 kcal mol^−1^) and cyclohexanol^13*a*^ (alcohol substrate, BDE 92.4 kcal mol^−1^) were used as substrates due to their comparatively higher water-solubility and the presence of C–H bonds with BDEs >90 kcal mol^−1^ (Scheme 2) (conventional benchmarked substrates like cyclohexane, 2,3-dimethylbutane, toluene, *etc.* were not used since they were almost insoluble in >95% water). Oxidation under single turnover conditions also led to the selective formation of 6-hydroxy-6-methylheptan-2-yl isonicotinate and cyclohexanone in 32 and 75% yield respectively (in cyclohexanol the yields were greater than the theoretical yield of 50% since a slight excess of *m*CPBA was used). The second-order rate constants for 6-methylheptan-2-yl isonicotinate and cyclohexanol at 10 °C were determined to be 14.4 (Fig. S50†) and 90.55 M^−1^ s^−1^ (Fig. S44†) respectively. The kinetic isotope effect (KIE) for the oxidation of cyclohexanol at 10 °C was determined to be 10 (Fig. S44†), ensuring that the rate-determining step involves H-atom abstraction from the C–H bond by 3, similar to that observed for 1A. Moreover, the ^18^O labelling experiment was performed on the oxidation of toluene, using H_2_O^18^ as the solvent, resulting in oxygen incorporation from 3 into the substrate toluene, yielding 45% ^18^O enriched product [a previous study^13*d*^ shows that <5% ^18^O is incorporated from the exchange of benzaldehyde with H_2_O^18^] with benzaldehyde, thereby supporting a rebound mechanism (Fig. S51†).^13*b*^ The absence of exclusive formation of ^18^O-benzaldehyde negates the possibility of a direct water attack.

The second order rate constants of cyclohexanol and 6-methylheptan-2-yl isonicotinate indicated that these reactions performed in 100% water were ∼281 times and ∼350 times faster, respectively, as compared to that in 97 : 3 CH_3_CN : H_2_O medium (Fig. S42 and S45†). We have earlier reported up to 70-fold rate enhancements in C–H oxidation by 1A′ (oxoiron(v) formed from regular FebTAML 1A) upon the addition of water in CH_3_CN.^13*b*^ To further understand the role of water, the solvent kinetic isotope effect (SKIE) was estimated for cyclohexanol oxidation with D_2_O as the solvent maintaining pH using a buffered solution. An SKIE (*k*_H_2_O_/*k*_D_2_O_) value of 2 was observed in 100% D_2_O (Fig. S48†), indicating the involvement of possible hydrogen bonding in the transition state. Earlier DFT studies have indicated that the high reactivity upon the addition of water resulted from the preferential stabilization of the rate-determining transition state in 100% water.^13*b*^ We believe that in 100% water, the transition state during the C–H abstraction is significantly stabilized resulting in very high reaction rates in comparison to CH_3_CN. Such stabilization of transition states by water molecules may also occur in enzymes since the reaction also occurs in 100% water.

### Activation of H_2_O_2_: formation of oxoiron(v) and its regioselective hydroxylation of C–H bonds

Intrigued by the absence of dimer formation as well as the faster reaction rate in C–H bond hydroxylation, we perceived the need to perform the same oxidation by a more natural oxidant (after dioxygen) in the form of hydrogen peroxide, which is also a 2e^−^ reduced surrogate of O_2_.^2*b*^

**Fig. 4:**
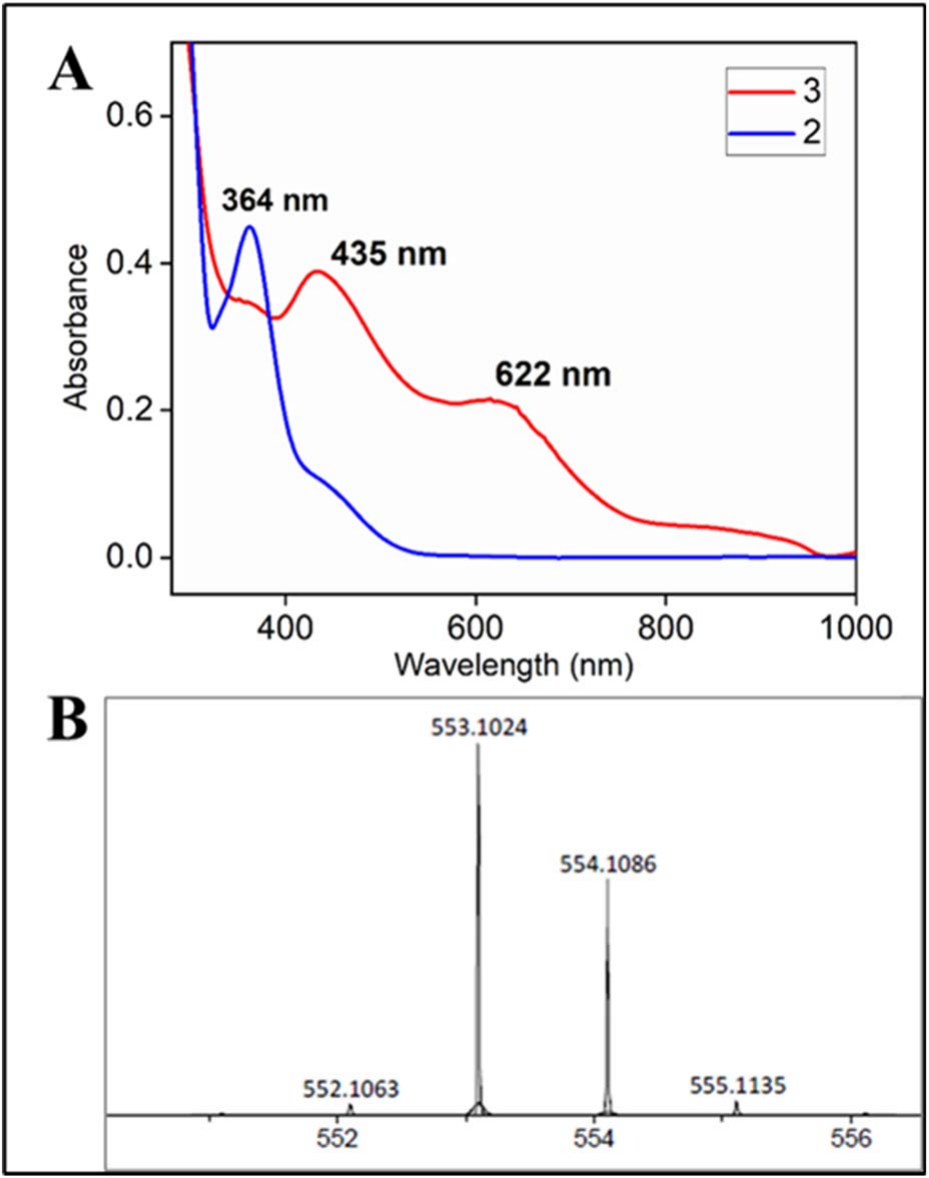
(A) UV-Vis spectra of 3 (red) formed upon addition of H_2_O_2_ to 2 in CH_3_CN–H_2_O (1 : 1) solvent (B) HRMS of 3 formed upon addition of H_2_O_2_ to 2 in 80 : 20 acetonitrile–water medium; experimental *m*/*z* (M^−^) 553.1024 (100%); calc'd *m*/*z* (M^−^) 553.1049 (100%).

Recent studies by our group have demonstrated that oxoiron(v) can only be formed *via* the addition of strong oxidants like mCPBA and NaOCl to 1.^5*b*,13^ In contrast, the addition of hydrogen peroxide to 1 resulted in the formation of unreactive and non-catalytic μ-oxo-dimeric species (mixture of Fe^III^Fe^IV^ and Fe^IV^Fe^IV^, characteristic broad peak at ∼900 nm) (Fig. S56†), which were unreactive towards strong C–H bonds.^5*d*,9*b*,9*d*^ Herein, the addition of aqueous hydrogen peroxide to 2 in 1 : 1 CH_3_CN–H_2_O (phosphate buffer pH 8.5) medium resulted in a green coloured species with the characteristic UV-Vis spectrum (*λ*_max_ of 435 nm and 625 nm) (Fig. 4A), HRMS (*m*/*z* 553.01165) (Fig. 4B) and EPR spectrum (*g* values: 2.13, 2.09 and 1.98); reminiscent of 3 (Fig. S53†).

**Fig. 5:**
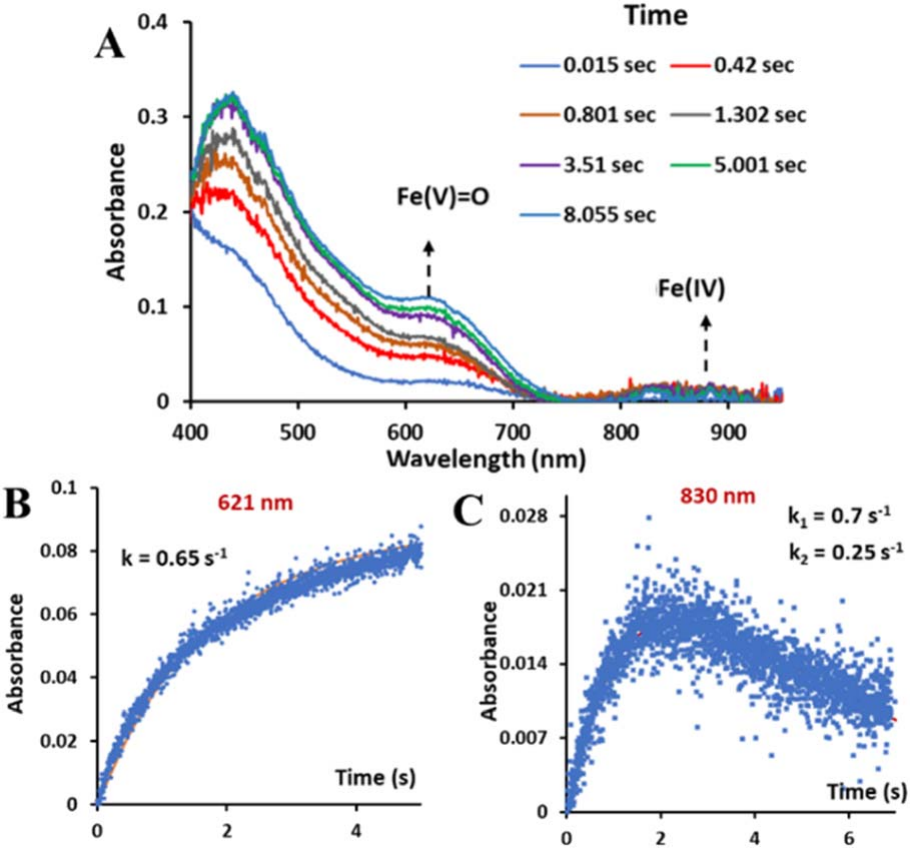
(A) Absorption spectra of 3 formed upon addition of H_2_O_2_ to 2 in CH_3_CN–H_2_O (1 : 1) solvent. (B) Reaction kinetics upon addition of H_2_O_2_ to 2 in CH_3_CN–H_2_O (1 : 1) solvent, with *λ*_max_ at 621 nm. (C) Reaction kinetics upon addition of H_2_O_2_ to 2 in CH_3_CN–H_2_O (1 : 1) solvent, with *λ*_max_ at 830 nm.

Stop flow kinetics of the reaction of 2 with hydrogen peroxide in 1 : 1 CH_3_CN–H_2_O (phosphate buffer pH 8.5) showed the formation of both the oxoiron(v) species, with *λ*_max_ at 620 nm, and a minor monomeric Fe^IV^ species, (as indicated in the Mössbauer data, Fig. S26†) with *λ*_max_ at 830 nm, simultaneously (Fig. 5A). The absorbance *vs.* time plots show that the “Fe(iv)” species initially formed decays with time while the concentration of oxoiron(v) increases with time continuously (Fig. 5B and C).

In 100% water medium, however, we were unable to fully form this intermediate under ambient conditions (4 to 27 °C), with hydrogen peroxide, as evident from both UV-Vis (Fig. S54†) and stop flow measurements at room temperature, since the highly reactive intermediate decomposed in the presence of excess H_2_O_2_. However, when 2 equiv. of H_2_O_2_ was added to 2 (0.2 mM) in 100% water (pH 8.5, 10 mM phosphate buffer) in the presence of 10 mM *cis*-dimethylcyclohexane, exclusive formation of the 3° hydroxylated product was observed in high yield. This result encouraged us to perform catalytic reactions in 100% water.

### Catalytic activity of 2 towards C–H hydroxylation with H_2_O_2_ in water

**Fig. 6:**
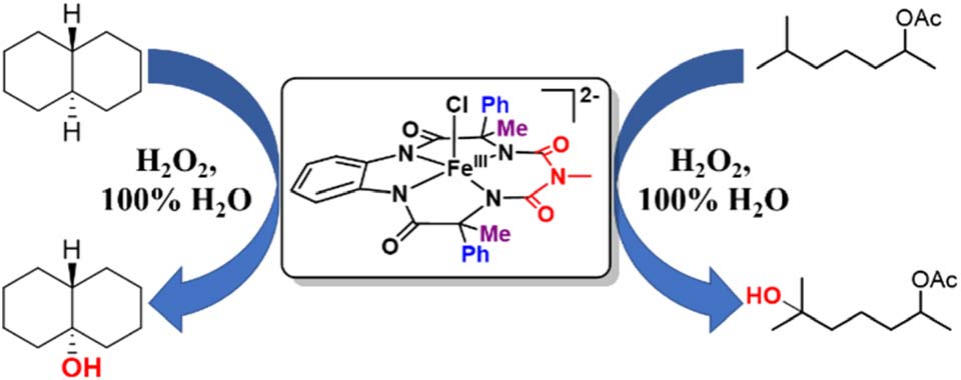
Selective hydroxylation of *trans*-DMCH and 6-methylheptan-2-yl acetate by catalyst 2 using H_2_O_2_ in 100% water.

Catalytic reactions were performed using the Fe-complex 2 (1–4 mol%), substrate (0.2–0.5 mmol scale), and H_2_O_2_ (2–3 equiv.), which was added using a syringe pump in a complete aqueous medium comprising pH 8.5 phosphate buffer (Fig. 6 and Table 1). A wide range of 3° C–H bond substrates including complex products were oxidized with low catalyst loadings (1–4 mol%) in 2–8 h. Here, 3° C–H bonds were found to be preferentially oxidized in the presence of statistically more important 2° C–H bonds with high retention of configuration (RC). Although these organic substrates are in general insoluble in an aqueous solvent system, good yields (except entries 13 and 14) along with very high retention of configuration (stereoselectivity) have been obtained.

**Table 1:** Substrate scope for C–H bond oxidation in water

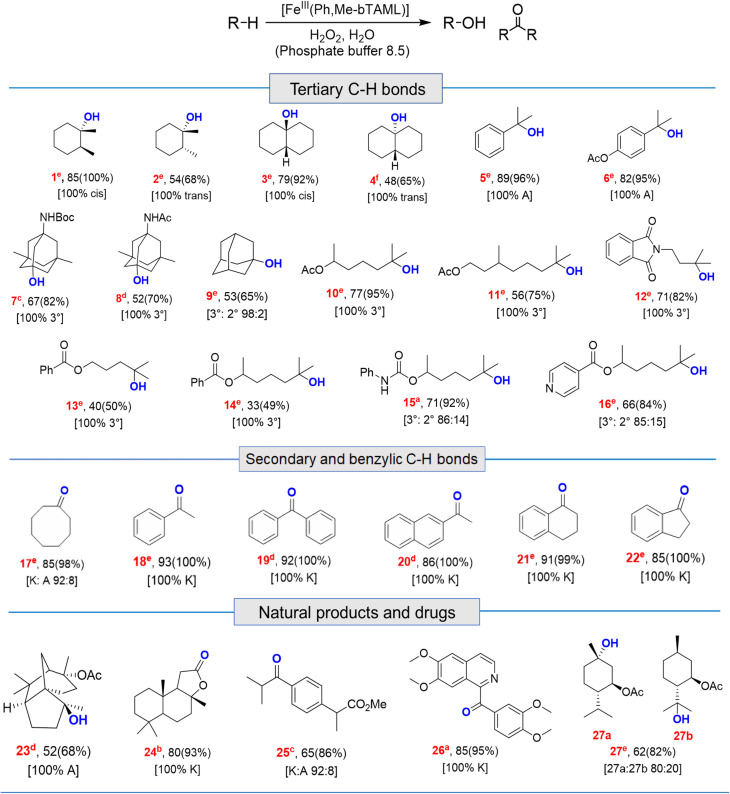

a 0.20 mmol.

b 0.25 mmol.

c 0.27 mmol.

d 0.30 mmol.

e 0.40 mmol.

f 0.50 mmol scale; A and K denote alcohol and ketone respectively. Loading: 2 mol% catalyst (entries 1, 3, 5, 6, 10, 15, 16, 17, 18, 19, 20, 21, 22 and 26); 3 mol% (entries 2, 4, 7, 8, 9,11, 23, 24 and 25); 4 mol% (entries 12, 27, 13 and 14); for hydroxylation, % isolated yield (GC conversion) [selectivity]. For details and structure determination see the ESI.

Both *cis*- and *trans*-isomers of dimethylcyclohexane and decahydronaphthalene (entries 1 to 4, Table 1) were solely hydroxylated at tertiary 3° C–H sites, along with 100% retention of configuration. Cumene (5), *para*-acetoxy substituted cumene (6), –Boc and acetate protected adamantane derivatives (entries 7 and 8, Table 1), and substrates containing acetate, benzoate, and phthalimide functionalities (entries 10 to 14, Table 1) were all preferentially hydroxylated at 3° C–H sites only, which is unprecedented, whereas, carbamate (15) and isonicotinate (16) containing substrates yielded little amount of ketone (14–15% w.r.t. 3° site). For menthyl acetate (27), oxidation occurred only at the two available 3° C–H positions (no ketone formation), leading to the formation of products 27a and 27b in 62% overall yield at a ratio of 4 : 1 in favor of the more sterically available 3° C–H bond. Natural products containing non-activated methylene sites which include (+)-cedryl acetate were hydroxylated very selectively at the least sterically hindered 3° C–H bond to give product 23 with an isolated yield of 52%. The substrates bearing activated methylenic C–H bonds like (−)-ambroxide and papaverine (opium alkaloid) were selectively oxidized at the 2° site to give (+)-sclareolide (entry 24, 72% yield; which is in contrast to the formation of 2-oxosclareolide by Fe-PDP complexes^3*d*^) and papaveraldine (entry 26, 85% yield; no formation of the undesired papaverine *N*-oxide) respectively. Besides, the benzylic position in the ibuprofen methyl ester (derivatized anti-inflammatory drug) was predominantly oxidized to the ketone (25) along with minor hydroxylation at the 3° C–H bond adjacent to the isopropyl group. In addition to this, cyclooctane, which contains only 2° C–H bonds, gets primarily oxidized to ketone (17) with an overall yield of 85% along with a minor hydroxylated product. Additionally, when cyclohexane was used as a substrate, cyclohexanol was predominantly formed (GC yield of 71% with a high A : K ratio of 9 : 1; Fig. S57†). Substrates containing benzylic C–H bonds like ethylbenzene, diphenylmethane, 2-ethyl naphthalene, tetralin, and indan were oxidized at the benzylic position with excellent yields to form ketone as products (entries 18 to 22, Table 1). In cyclohexane derivatives, the stereochemical orientation of the 3° C–H bonds (axial or equatorial) controls the rate of regioselective oxidation. Higher reactivity for *cis* isomers (entries 1 and 3) w.r.t. *trans* isomers (entries 2 and 4) is expected which can be attributed to the strain release in the transition state for the cis isomers, as was observed in our previous reports.^3*a*,*d*,14^ Remarkably, in our case, despite having a statistically higher number of methylenic C–H bonds, there is no 2° C–H bond oxidation accompanied by 100% RC, unlike observed previously for 1B catalyzed C–H bond oxidation using *m*CPBA^5*c*^ (where 98% RC was observed) and other previously reported non-heme complexes (Tables 3 and S8†). For catalysis with 1B, the ratio of oxidation at the tertiary C–H site *vs.* secondary C–H site (*i.e.* 3° : 2° ratio, or alcohol : ketone ratio) for entries 2 and 4 was reported to be around 3 : 1 and 2 : 3 respectively.^5*c*^ Thus, we believe that such regioselectivity with no secondary alcohol or ketone product has never been encountered before. Moreover, for substrate entries 10 and 27 (menthyl acetate), catalysis with 1B exhibited 25% and 15% 2° C–H bond oxidation (*i.e.*, 75% 3° and 85% 3° respectively), whereas catalysis with 2 showed none.

Apart from aliphatic C–H bond oxidation, epoxidation of alkenes was also performed (Table 2). Full conversion of alkenes to epoxides was obtained with very low catalyst loading and with high retention of configuration. Here, it can be noted that with 1A′, *via* the formation of dimeric species, alkene epoxidation was successful albeit in CH_3_CN solvent with modest yields.^13*c*^

**Table 2:** Substrate scope for alkene epoxidation in water^a^

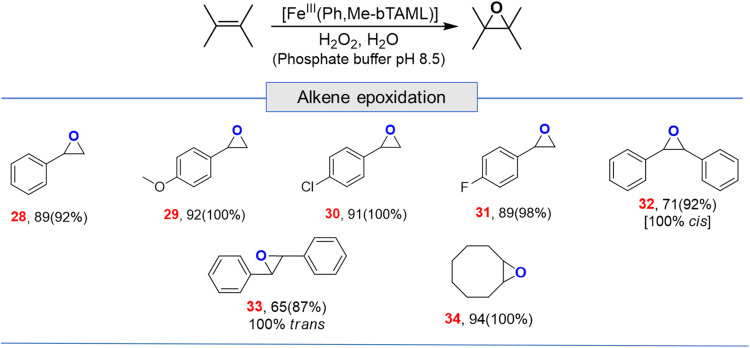

a For epoxidation, % GC yield based on standard (bromobenzene) (conversion)[selectivity], for alkenes 1 mol% loading (entries 28, 29, 30, 31 and 34); 2 mol% (entries 32 and 33); here selectivity was determined by GC.

**Table 3:** Comparison of selectivities of different substrates with previous reports using Cat 1B

Sl. no.	Reaction condition	% Isolated yield (GC conversion) [selectivity]		
		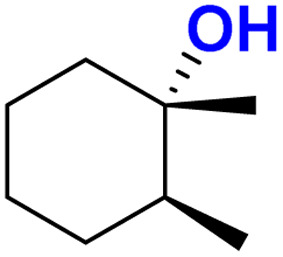	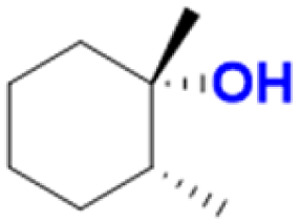	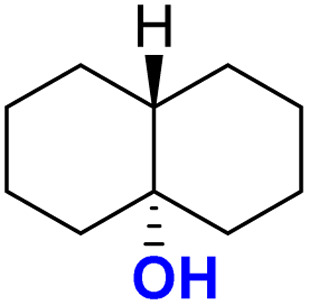
1	Water (100% buffer), Cat 2	85 (100%), [100% RC], [100% 3°]	54 (68%), [100% RC], [100% 3°]	48 (65%), [100% RC], [100% 3°]
2	CH_3_CN : water (4 : 1), Cat 1B	85(97%), [99% RC], [100% 3°]	60 (85%), [98% RC], [3° : 2° 76 : 24]	27 (90%), [98% RC], [3° : 2° 38 : 62]

## Conclusions

A new modified “picket fence” bTAML based iron complex was synthesized upon substituting one methyl group present in either wing of the macrocycle with one phenyl group. By doing this, we were able to fine-tune the reactivity of Fe-TAMLs from a peroxidase to a cytochrome P450 mimic. During oxidation reactions, this complex activated hydrogen peroxide to generate oxoiron(v), and thereby selectively oxidized strong C–H bonds in water medium (pH 8.5 buffer). The oxoiron(v) intermediate generated in water was around 300 times more reactive towards C–H bonds than in CH_3_CN under single turnover conditions. Catalytic oxidation was performed on substrates containing C–H and C=C bonds using H_2_O_2_ as the terminal oxidant in water. Several substrates (34 examples) that contained arenes, heteroaromatics, and polar functional groups were oxidized with predictable selectivity and stereoretention using steric, electronic, and stereoelectronic rules with moderate to high yields (50–90%), low catalyst loadings (1–4 mol%) and a small excess of H_2_O_2_ (2–3 equiv.). Mechanistic studies indicated the oxoiron(v) to be the active intermediate during the catalytic reactions and displayed unprecedented selectivity towards 3° C–H bonds. To summarize, for the first time, using H_2_O_2_ as the sole oxidant, an iron complex was synthesized that performs highly selective and efficient catalytic hydroxylation of strong C–H bonds and epoxidation of alkenes in 100% water. However, the exact nature of the oxoiron(v) in water is still not fully understood and requires further investigation.

## Data availability

All the data supporting this article have been uploaded as part of the ESI.†

## Author contributions

The concept of the work was designed by S. S. G. S. J. optimized and synthesized the molecular catalyst followed by its characterization. P. D. with the help of S. J. performed all the oxidation reactions and their optimizations with H_2_O_2_ along with kinetic studies with both H_2_O_2_ and *m*CPBA. C. D. performed stop flow UV experiments and Mössbauer analysis. A. D. and S. G. D. gave their inputs to improve the manuscript. S. J., P. D., and S. S. G. analyzed the data and wrote the manuscript. All authors have given approval to the final version of the manuscript.

## Conflicts of interest

There are no conflicts to declare.

## Supplementary Material

SC-014-D3SC03495J-s001

SC-014-D3SC03495J-s002
