# Supplementary material for: Highly regioselective oxidation of C–H bonds in water using hydrogen peroxide by a cytochrome P450 mimicking iron complex
Source: Chem Sci. 2023 Sep 8;14(38):10515–23. doi: 10.1039/d3sc03495j (PMC10548533; doi:10.1039/d3sc03495j)
Supplement: SC-014-D3SC03495J-s001 [file SC-014-D3SC03495J-s001.pdf]

Electronic Supplementary Material (ESI) for Chemical Science.
This journal is © The Royal Society of Chemistry 2023

Supporting Information

Highly Regioselective Oxidation of C–H Bonds in Water using Hydrogen Peroxide by a Cytochrome P450 mimicking Iron Complex

Sandipan Jana, ‡[a] Puja De, ‡[a] Chinmay Dey,[b] Somdatta Ghosh Dey,*[b] Abhishek Dey,*[b] and Sayam Sen Gupta*[a]

[a]Department of Chemical Sciences, Indian Institute of Science Education and Research, Kolkata; Mohanpur 741246, India.

[b]School of Chemical Sciences, Indian Association for the Cultivation of Science, Kolkata, West Bengal 700032

[‡] These authors contributed equally to this work.

Sl. No.	Table of contents	Page No.
1	Material and Methods	S2
2	General Instrumentations and Techniques	S2-S5
3	Experimental Results	S6
3A	Synthesis of 2	S6
3B	Synthetic Procedures and Characterization Data	S6-S29
3C	Reactivity Studies	S29-S30
3D	Kinetic Studies	S31-S33
3E	Oxoiron(V) intermediate formation by mCPBA treatment in 100% water and its kinetic study	S33-S40
3F	High valent oxo intermediate formation by H_2O_2 treatment	S40-S42
3G	Procedure for preparative hydroxylation reaction	S42
3H	Epoxidation Reaction Procedure	S42
3I	Substrate scopes for C-H oxidation and epoxidation in CH_3CN-water (1:1) solvent	S42-S46
3J	Comparison Studies	S46-S48
3K	Product Characterization Data	S48-S76
4	References	S76

1. Materials and Methods

Aqueous sodium hypochlorite solution (4% Chlorine), Merck, was used directly without any purification, meta-Chloroperoxybenzoic acid (*m*CPBA, Sigma Aldrich 77%) was purified following an established methodology.[1] Aqueous sodium hypochlorite (4 % available chlorine, Merck) (NaOCl) was used as purchased. Hydrogen peroxide (30%, Merck) (H_2O_2) was also used as purchased. Acetonitrile (LC-MS and HPLC grade, Sigma Aldrich) was passed through an activated neutral alumina column and distilled and used thereafter. All the other solvents required were purchased and used after distillation over drying agents. All the substrates required for hydroxylation and epoxidation were bought from TCI, Sigma Aldrich or Thermo-fischer scientific. Unless mentioned separately, the reactions were performed at room temperature under air. Analytical thin layer chromatography (TLC) was performed on TLC Silica gel 60 F254. Visualization was accomplished with short wave UV light or with ninhydrin and PMA staining solutions followed by heating. Purification of desired products were performed by column chromatography which was performed on silica gel (230-400 mesh or 100-200 mesh) by standard techniques eluting with solvents as indicated. NMR solvents were dried using pre-activated 4 Å molecular sieves. All indicated solvents were purified prior to use as per standard literature protocols. All the organic chemicals required for ligand synthesis (Sigma Aldrich, TCI India, Spectrochem) were used as received unless synthesized or mentioned; their purity was checked by GC. H_2O^{18} was purchased from Icon isotopes. For electrochemical experiments, Glassy Carbon, Platinium (wire and mesh), Ag/$AgNO_3$ (Non-aqueous), and Ag/AgCl (sat. KCl) electrodes were purchased from CH Instruments.

2. General Instrumentations and Techniques

^1H and ^{13}C NMR spectra were recorded on Bruker AV 400 or JEOL 400 instruments in solvents as indicated. Chemical shifts (δ) are given in ppm. The residual solvent signals ($CDCl_3$ or DMSO-d_6) were used as references and the chemical shifts converted to the TMS scale ($CDCl_3$: $\delta H = 7.26$ ppm, $\delta C = 77.16$ ppm, or DMSO-d_6: $\delta H = 2.50$ ppm, $\delta C = 39.51$ ppm).

All the cyclic voltammetry studies were carried out in CHI-660 potentiostat. UV−vis spectral studies were carried out using an Agilent diode array Cary-8454 spectrophotometer with an attached electrically controlled thermostat. GC-MS was performed on a Thermo Scientific ISQ QD Mass Spectrometer attached with Thermo Scientific TRACE 1300 gas chromatograph using an HP-5 ms capillary column (30 m × 0.25 mm × 0.25 μm, J&W Scientific) with helium as the carrier gas. HRMS was performed in a Bruker micrOTOF-Q II Spectrometer.

2A. EPR Measurements

Electron Paramagnetic Resonance (EPR) spectroscopy was studied by a Bruker spectrometer (Bruker, EMXmicro) operating at X-band frequency (Microwave frequency 9.31 GHz) and magnetic field modulation of 100 kHz, with a microwave power of 48.83 mW and modulation amplitude of 10 G at 85 K.

Treatment with mCPBA in CH_3CN: 3 mM **2** in CH_3CN (200 µL) was taken in an EPR tube and the measurement was taken. For **3**, the same sample was then cooled to sub-zero temperature by intermittent cooling with liquid nitrogen followed by addition of 2 equiv *m*CPBA, and the measurement was taken at 100 K.

Treatment with mCPBA in water: 4 mM **2** in water (200 µL) was taken in an EPR tube followed by the addition of 20 mM pH 8.5 phosphate buffer. The EPR was cooled at sub-zero temperature by intermittent cooling with liquid N_2 (no freezing of the sample). Then 4 eq. *m*CPBA was added to the EPR tube, mixed properly and frozen it immediately and lastly given for measurement at 85 K.

Treatment of Hydrogen peroxide: 8 mM **2** in acetonitrile (80 µL) was taken in an EPR tube followed by the addition of aq.10 mM pH 8.5 phosphate buffer (80 µL). The EPR was cooled at sub-zero temperature by intermittent cooling with liquid N_2 (no freezing of the sample). Then 5 eq. H_2O_2 was added to the EPR tube and mixed properly and given for measurement at 85 K.

2B. Mössbauer Measurements

Mössbauer spectra were recorded using an alternating constant Wissel Mössbauer spectrometer, consisting of an MR 360 drive unit, an MVT 1000 velocity transducer, and an LND-45431 proportional counter mounted on an MB-600 Mössbauer bench with cryostat stand. The system was operated in horizontal transmission geometry with source (^{57}Co in Rh-matrix), absorber, and detector in a linear arrangement. The temperature was controlled and maintained using MBBC-N20106 Mössbauer cryostat for liquid-nitrogen connected with Lake Shore-325 temperature controller unit. Measurements were performed at 77 K. Data acquisition was performed using a 512 channel analyzer. Isomer shifts were referenced versus α-iron metal foil at the same temperature. The simulation of experimental data was performed using the Normos Site and Dist.

2C. Electrochemical Measurements

Cyclic voltammetry and differential pulse voltammetry experiments were carried out in CHI-660 potentiostat instrument. Solutions of **2** were placed in simple undivided three electrode cells. Glassy Carbon (GC) (3 mm of diameter) was used as working electrode, Silver/Silver nitrate (10 mM) as reference electrode (unless explicitly mentioned) and Pt wire as counter electrode. Before each measurement, working electrode was polished with 0.05 µm alumina paste and rinsing with water/acetone and finally blow-drying.

2D. Single Crystal X-ray Diffraction (SCXRD) Measurements

X-ray diffraction data for $(Et_4N)_2[Fe\text{-}(Ph,Me\text{-}bTAML)]^{2-}$ (**2**) crystal was collected at 100 K on a SuperNova, Eos diffractometer using monochromatic Mo-Kα radiation (λ = 0.71073 Å) and Cu-Kα radiation (λ = 1.54178 Å) having a 300 µm beam size. Using Olex2 (1.2.9 version)[2] the structure was solved using the SHELXT[3] structure solution program following an intrinsic phasing solution method and refined with the ShelXL[4] refinement package using Least Squares minimization. All the non-hydrogen atoms were refined anisotropically. All the crystal packing diagrams were prepared using Mercury (3.10.1 version) software.

2E. UV-Vis experiments

Preparation of 3 by *m*CPBA or NaOCl: $Fe^{V}(O)$, **3**, was prepared quantitatively at room temperature from the parent TAML activator, $(Et_4N)_2[Fe^{III}(Cl)(Ph,Me\text{-}bTAML)]$ (0.1 mM), **2**, in CH_3CN by adding 1.2 equiv of mCPBA or NaOCl.

Preparation of 3 by H_2O_2: 3 was prepared at room temperature from the parent TAML activator, $(Et_4N)_2[Fe^{III}(Cl)(Ph,Me\text{-}bTAML)]$ (0.1 mM), **2**, in $CH_3CN:H_2O$ (3:2), where the water part contains pH 8.5 phosphate buffer (5 equiv, 0.5 mM) by adding 2.5 to 5 equiv of H_2O_2 (0.25-0.50 mM). The stock solution of the Fe^{III} complex was prepared in acetonitrile whereas the stock solutions of phosphate buffer and hydrogen peroxide were made in distilled water. Same spectra were obtained when the acetonitrile-water ratio was varied from 4:1 to 1:1.

Half-equivalent oxidant addition and comproportionation reaction: 0.1 mM of **2** in acetonitrile was subjected to 0.5-0.6 equivalents of mCPBA and analyzed through UV-vis spectrometer. For the comproportionation reaction, equimolar amounts (0.1 mM in acetonitrile) of both **2** and **3** (from *m*CPBA) were mixed and UV-vis spectra were recorded.

2F. Rate Measurements from UV-Vis Kinetics

Self-decay of 3 in CH_3CN: The spontaneous reduction or the self-decay of **3** to Fe^{IV}/Fe^{III} is monitored using UV spectral decay of its characteristic 625 nm band. The initial rate of decay was estimated to be first order with respect to the concentration of **3** and the decay first order rate constant ($k_{5/4,3}$) was determined from the slope of the straight lines (obtained from several concentrations of **3**) at 298 K. The decay constant (k_{decay}) value at 298K (6.0531×10^{-4} s^{-1}; $t_{1/2}$ of 19 mins) was found to be higher compared to **1A′** reported at the same temperature (Figure S33).

Second-order rate constant (k_2) determination in CH_3CN: The room temperature stability of **3** has prompted us to study the oxidation of strong and weak C-H bonds whose bond dissociation energies range from 97-75 kcal/mol. Starting from 2,3-dimethylbutane (BDE 96.5 kcal/mol) to xanthene (BDE 75.5 kcal/mol), we have performed the single turnover kinetics of five substrates wherein *m*CPBA is used for the formation of **3** (Figures S34 and S35). The reactions follow second order and the rates of these reactions were found to be around three times faster than the rates obtained for **1A′** (Table S3). The rate constants also vary linearly with BDEs of the substrates. Also, the linear free energy relationship was determined by using Bells-Evans-Polanyi (BEP) relation (Figure S36) where the plot was found to be linear for all the substrates from toluene to xanthene (slope -0.15); similar to the slope in case of **1A** (slope -0.17). This linearity supports hydrogen atom abstraction from C–H bond by **3** in the rate-determining step, as has been reported earlier.

Stopped Flow UV Kinetics: Stopped-flow analysis of the reaction of complexes **1A** and **2** with H_2O_2 were performed on SFM 4000 stopped-flow absorption spectrophotometer (light source Xe lamp). The reactions were performed by mixing 0.25 mM of these complexes with

1.25 mM H_2O_2 in 1:5 concentration ratio. The kinetic trace of 621 nm and 830 nm were followed.

2G. Magnetic moment measurements (Evans method NMR)

The Evans method makes use of differences in the ^{1}H NMR chemical shift in a solvent caused by the presence of a paramagnetic species. It can be used to calculate the magnetic moment of the species and the number of unpaired electrons present. Two coaxial NMR tubes were used in which a CD_3CN solution of the paramagnetic sample is introduced into the outer tube, and only CD_3CN (without the sample) is filled in the inner tube. The concentration of TMS was identical in both tubes. The NMR spectra were recorded in a 400 MHz NMR instrument. The effective spin-only magnetic moment was calculated by a simplified Evans method analysis[17] according to the following equation:

$$\mu_{eff} = 0.0618\sqrt{\frac{\Delta\nu \mathrm{T}}{2\mathrm{fM}}}$$

where f is the oscillator frequency (MHz) of the superconducting spectrometer (f = 400 MHz), T is the temperature (K) (T = 297 K), M is the molar concentration of the paramagnetic metal complex, and $\Delta\nu$ is the difference in frequency (Hz) between the two reference (TMS) signals.

3. Experimental Results

3A. Synthesis of 2:

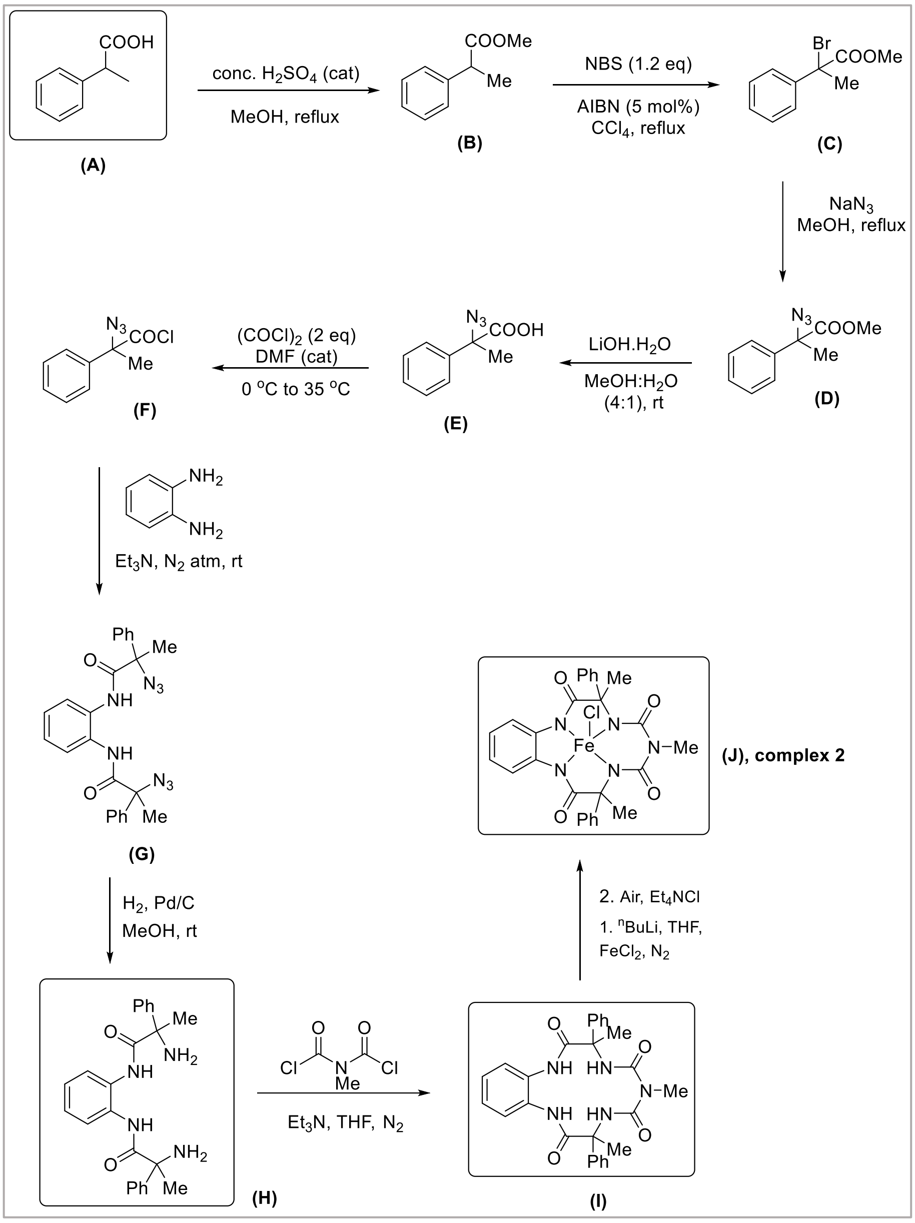

Scheme S1: Synthesis of a modified bTAML system (Ph,Me-bTAML) and its corresponding Fe complex

3B. Synthetic Procedures and Characterization Data

Methyl 2-phenylpropanoate[6] (B)

2-phenylpropanoic acid (A) (6 g, racemic) was dissolved in methanol (54 mL) in a 250 mL RB. Conc. Sulphuric acid (H_2SO_4) (98%, 300 µL) was then added and the reaction was refluxed (65 °C) for 8 h. After completion (checked by TLC), the reaction solution was allowed to come at room temperature and was concentrated to half volume. Hexane (100 mL) was added

followed by sat. aq. $NaHCO_3$ (30 mL) solution. The organic layer was separated via separating funnel and aqueous part was discarded. The organic part was again washed once with water and once with brine (sat. NaCl solution), and then dried with anhydrous Na_2SO_4, and concentrated to get pure product (B) as viscous liquid. Yield quantitative, 6 g (92%). TLC done in 10% EA-hexane.

^{1}H NMR (400 MHz, CDCl3) δ 7.37-7.28 (m, 5 H), 3.78-3.73 (q, J = 7.2 Hz, 1 H), 3.69 (s, 3 H), 1.54-1.52 (d, J = 8 Hz, 3 H).

^{13}C NMR (400 MHz, CDCl3) δ 174.98, 140.55, 128.62, 127.45, 127.11, 51.98, 45.41, 18.57

GC-MS (EI) m/z: M^+ peak:164.12

^{1}H NMR:

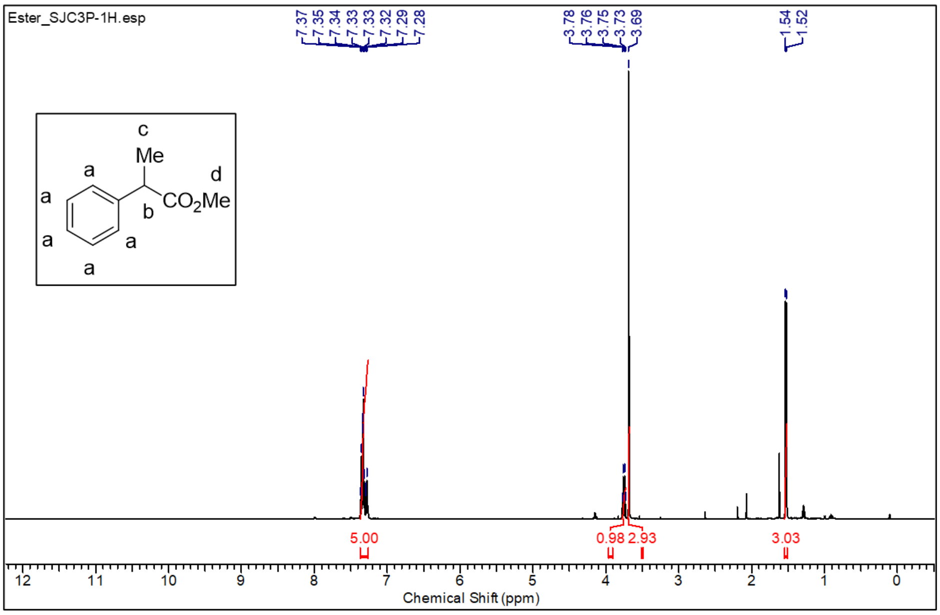

Figure S1. ^{1}H NMR of B

13C NMR:

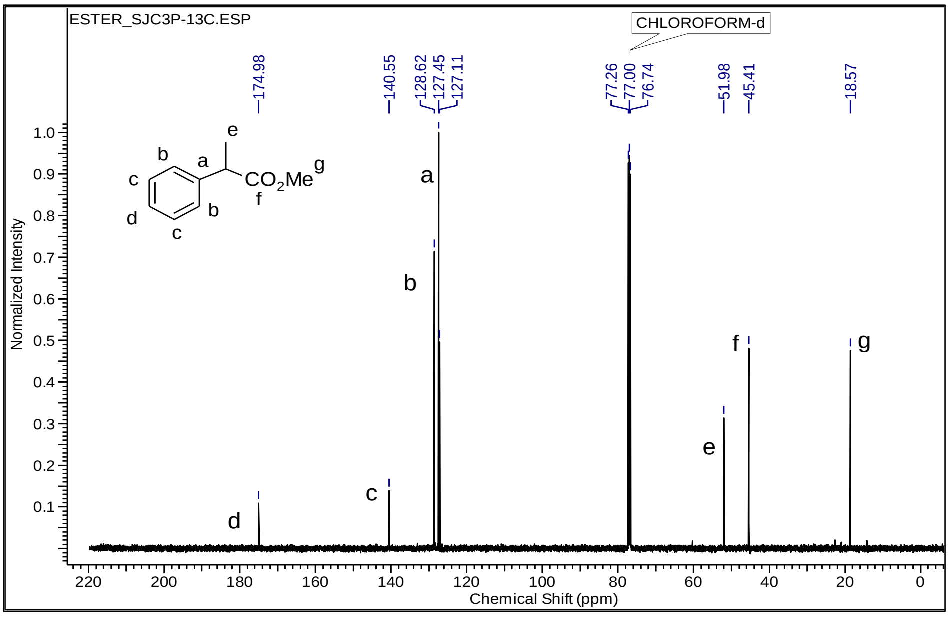

Figure S2. ^{13}C NMR of B

Methyl 2-bromo-2-phenylpropanoate[7] (C)

In a 250 mL RB, 60 mL carbon tetrachloride (CCl_4) was added to 4 g of ester (B) followed by addition of 5.2 gm (1.2 eq) of N-bromosuccinimide (NBS) and 200 mg (5 mol%) AIBN. The reaction mixture was refluxed at 80 °C for 6-8 h. The colour changes to orange with time. After completion, the reaction mixture was allowed to cool till room temperature and then filtered via frit funnel using celite. The filtrate was concentrated and purified by column chromatography to get pale yellow liquid as product. Yield quantitative, 5.7 g (96%). TLC at 10% EA-hexane.

Ref: PCT Int. Appl. (2011), WO 2011149921 A1 Dec 01, 2011

^{1}H NMR (400 MHz, CDCl3) δ 7.57-7.32 (m, 5H), 3.81 (s, 3H), 2.32 (s, 3H)

^{13}C NMR (400 MHz, CDCl3) δ 171.50, 140.94, 128.37, 126.71, 61.71, 53.56, 31.34

GC-MS (EI) m/z: M^+ peak: 241.97, 243.98

^{1}H NMR:

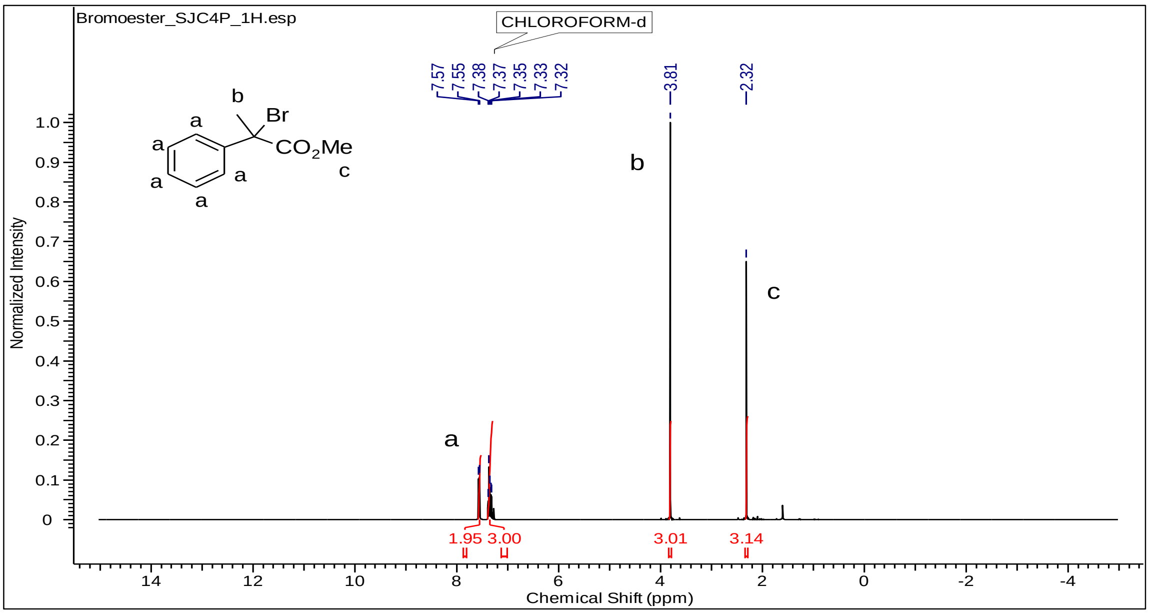

Figure S3. ^{1}H NMR of C

^{13}C NMR:

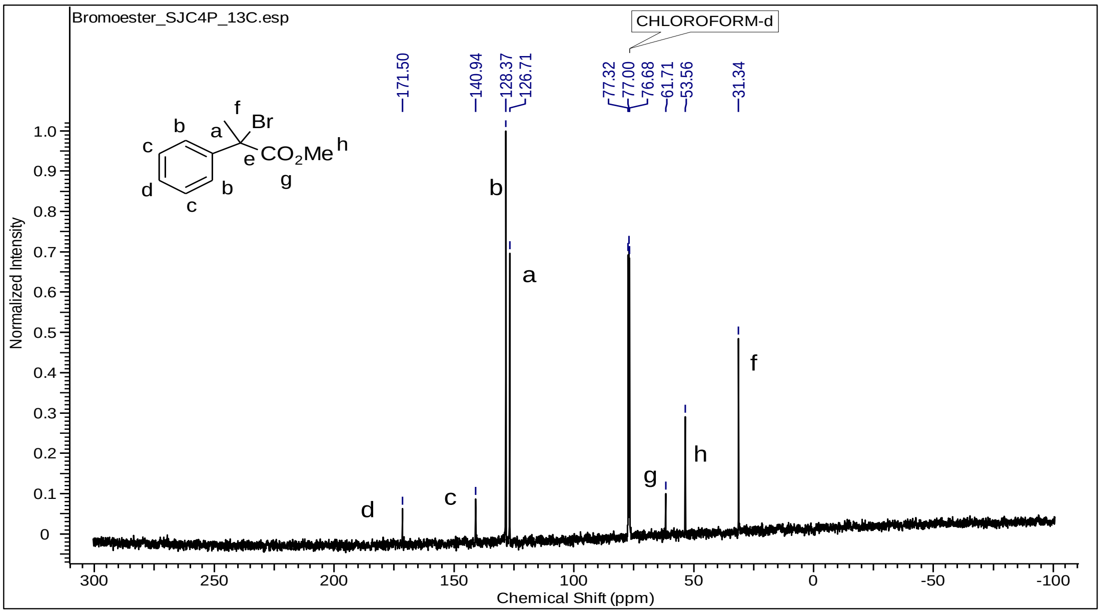

Figure S4. ^{13}C NMR of C

IR Data of Bromo Ester:

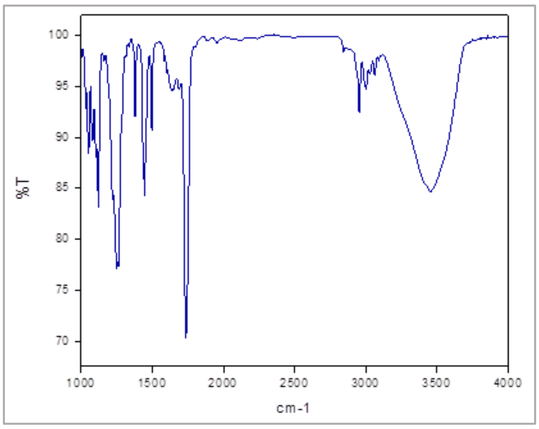

Figure S5. IR spectrum of C

Methyl 2-azido-2-phenylpropanoate (D)

To a solution of 4 g bromo-ester (C) in 80 mL methanol, taken in a 250 mL RB, was added 5 eq. sodium azide (5.3 g), and the mixture was refluxed at 65 °C for 12 h. After completion, most of the methanol was rota-evaporated (rem. 20 mL), and the mixture was diluted with 100 mL ethyl acetate. It was then washed with brine solution (2-3 times), where excess sodium azide got separated. The organic part was dried with anhydrous sodium sulphate and concentrated to get pure product as yellow viscous liquid. Yield obtained was 3.2 g (95%). TLC done at 10% EA-hexane.

^{1}H NMR (400 MHz, CDCl3) δ 7.41-7.42 (m, 5H), 3.81 (s, 3H), 1.84 (s, 3H)

^{13}C NMR (400 MHz, CDCl3) δ 171.99, 138.83, 128.80, 128.52, 125.57, 69.23, 53.11, 24.36

^{1}H NMR:

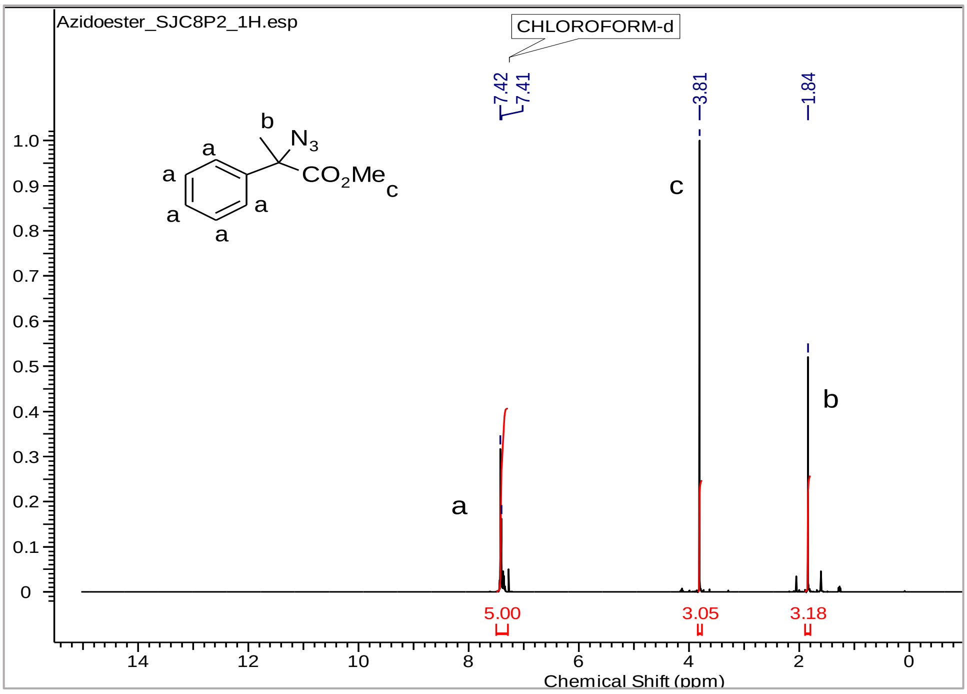

Figure S6. ^{1}H NMR of D

^{13}C NMR:

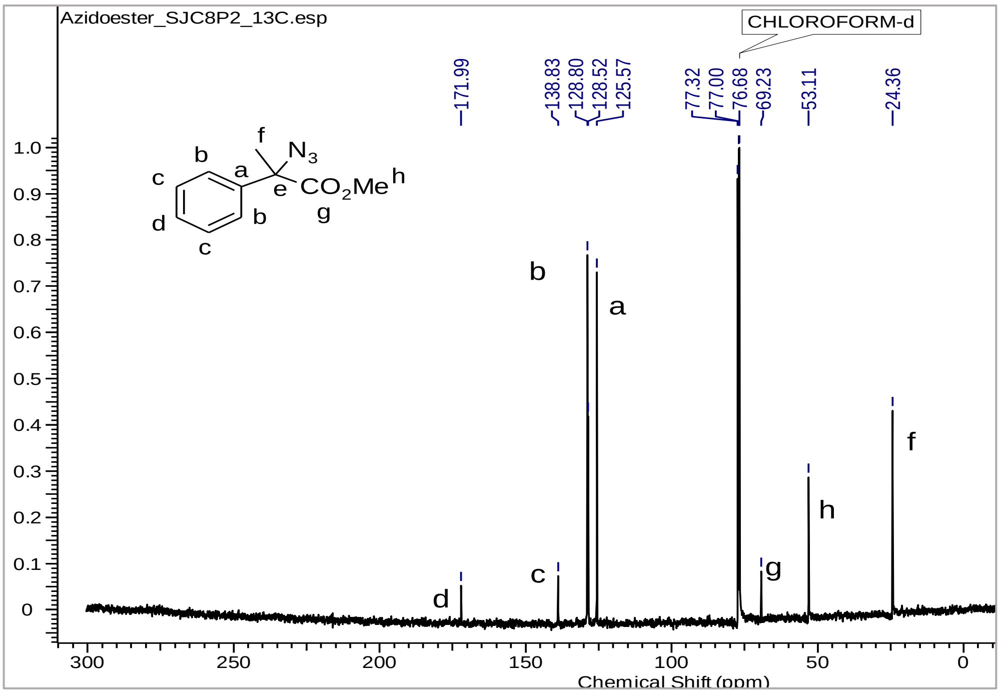

Figure S7. ^{13}C NMR of D

IR Data of Azido Ester:

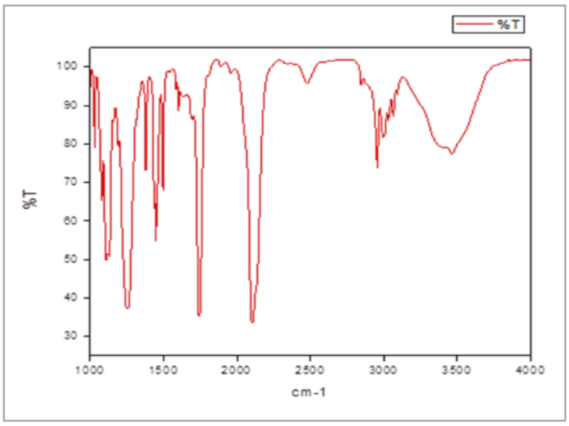

Figure S8. IR spectrum of D

Comparison between bromo and azido ester (2100 cm^{-1} peak for azido group):

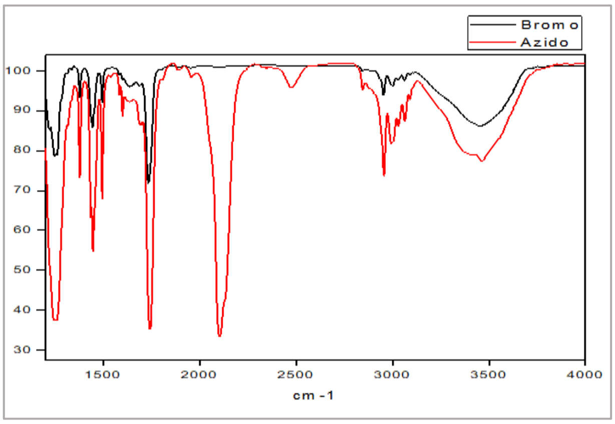

Figure S9. Comparison of IR spectra between C and D

2-Azido-2-phenylpropanoic acid (E)

In a 100 mL RB, 3 g of azido ester (D) was taken and 50 mL MeOH-water (4:1) was added, followed by the addition of 1.84 g (3 eq.) of lithium hydroxide monohydrate. The mixture was stirred at room temperature for 8 h. After completion, the reaction was quenched with 60 mL 1(N) cold HCl till pH reaches 1-2. Then, the resulting mixture was extracted with ethyl acetate (4 times). The combined organic part was washed with brine and then dried (Na_2SO_4) and concentrated to get semi-liquid product. TLC done at 10% EA-hexane.

^{1}H NMR (400 MHz, CDCl3) δ 7.51-7.40 (m, 5H), 1.94 (s, 3H)

^{13}C NMR (400 MHz, CDCl3) δ 176.64, 138.07, 128.87, 125.75, 68.84, 23.84

FT-IR: Characteristic azido group peak at 2100 cm^{-1}

^{1}H NMR:

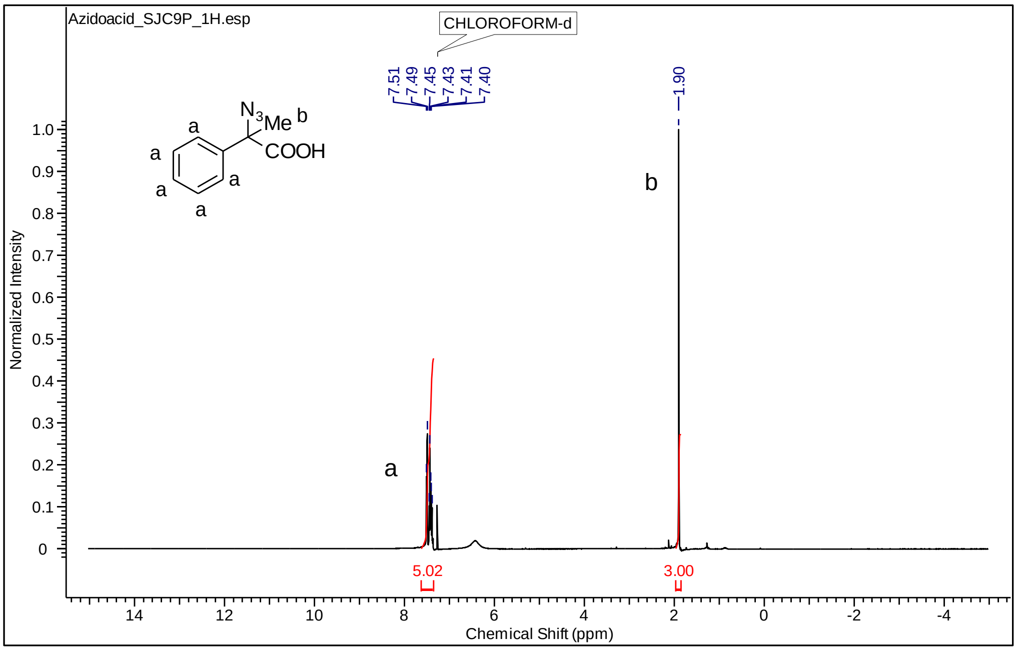

Figure S10. ^{1}H NMR of E

^{13}C NMR:

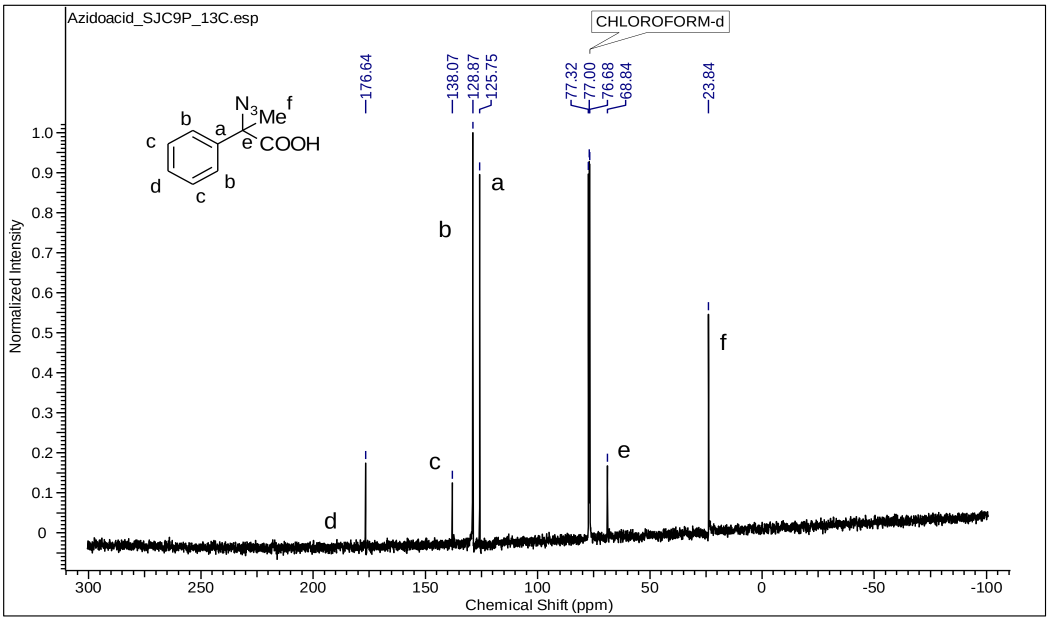

Figure S11. ^{13}C NMR of E

2-Azido-2-phenylpropanoyl chloride (F)

In a 100 mL 2-neck RB, 2.54 g of E was taken and 30 mL dry DCM was added under inert atmosphere. At 0 °C, 2.3 mL oxalyl chloride (2 eq.) was added slowly followed by addition of catalytic amount (250 µL) of DMF (strong effervescence was observed after DMF addition). 0 °C was maintained for 15 mins. Then, the reaction was allowed to come to room temperature and slightly heated to 35 °C (with warm water). The reaction was continued for 4 h. The solvent was then evaporated by using a vacum trap in liquid nitrogen. The RB content was washed

with hexane twice followed by drying under high vacuum. It was used as such for the next step (calculated 80% yield).

N,N'-(1,2-phenylene)bis(2-azido-2-phenylpropanamide) (G)

Everything was under N_2 atmosphere. Acid chloride (F) solution (2.2 eq) was prepared by adding 35 mL dry THF in that 2-neck RB used in the previous step. In another 250 mL 2-neck RB, 560 mg of 1,2-phenylene diamine (1 eq) was taken and 30 mL dry THF was added to it. At 0 °C, 1.75 mL of dry triethylamine (2.4 eq.) was added to the diamine solution. The acid chloride solution was then added dropwise to the amine solution (for 20 mins). After addition, the temperature was maintained at 0 °C for 10 more mins, followed by stirring for 15 h at room temperature. After completion, the reaction mixture was filtered via frit funnel to remove insoluble salts. The filtrate was rota-evaporated to complete dryness, followed by the addition of sufficient amount of DCM to solubilize it well. The DCM solution was washed twice with 1(N) HCl and twice with sat aq. $NaHCO_3$ solution and once with brine. The solution was then dried with Na_2SO_4 and concentrated to get pure G as a dark coloured semi-liquid compound. The yield obtained was 75% w.r.t. amine.

^{1}H NMR (400 MHz, CDCl3) δ 8.62 (s, 2H), 7.34-6.94 (m, 10H), 1.84 (s, 3H), 1.79 (s, 3H)

^{13}C NMR (400 MHz, CDCl3) δ 169.76, 138.76, 129.68, 129.68, 129.61, 128.66, 128.44, 126.26, 125.77, 125.05, 124.88, 22.62, 22.43

HRMS: 455.1928 (M+H) [calc'd 455.1938 (M+H)]

^{1}H NMR:

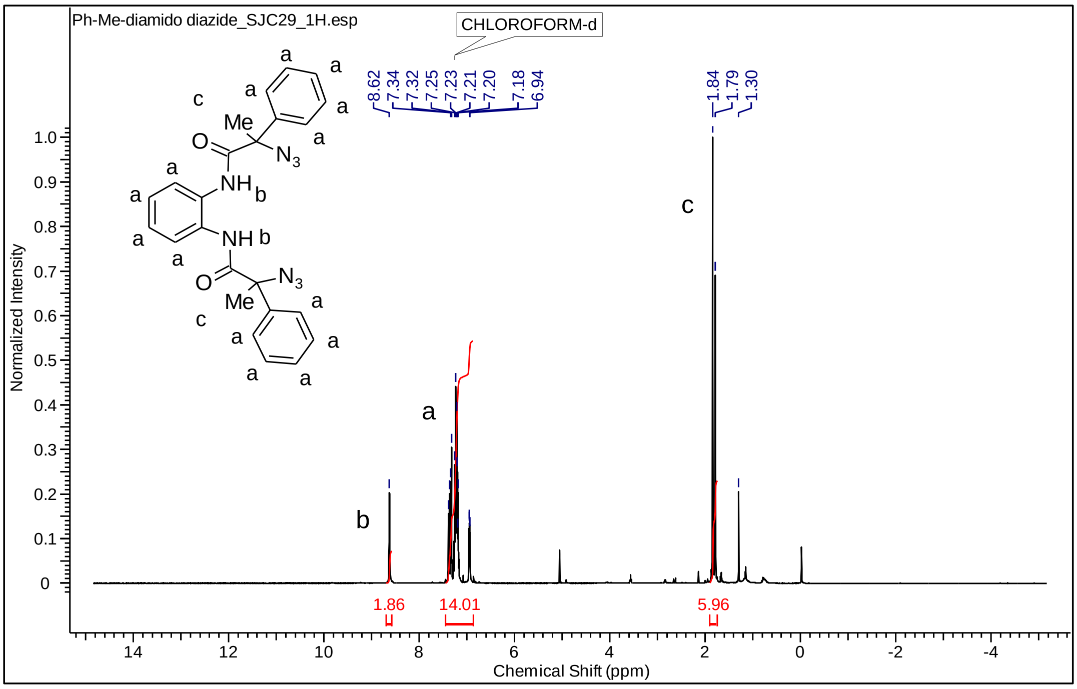

Figure S12. ^{1}H NMR of G

^{13}C NMR:

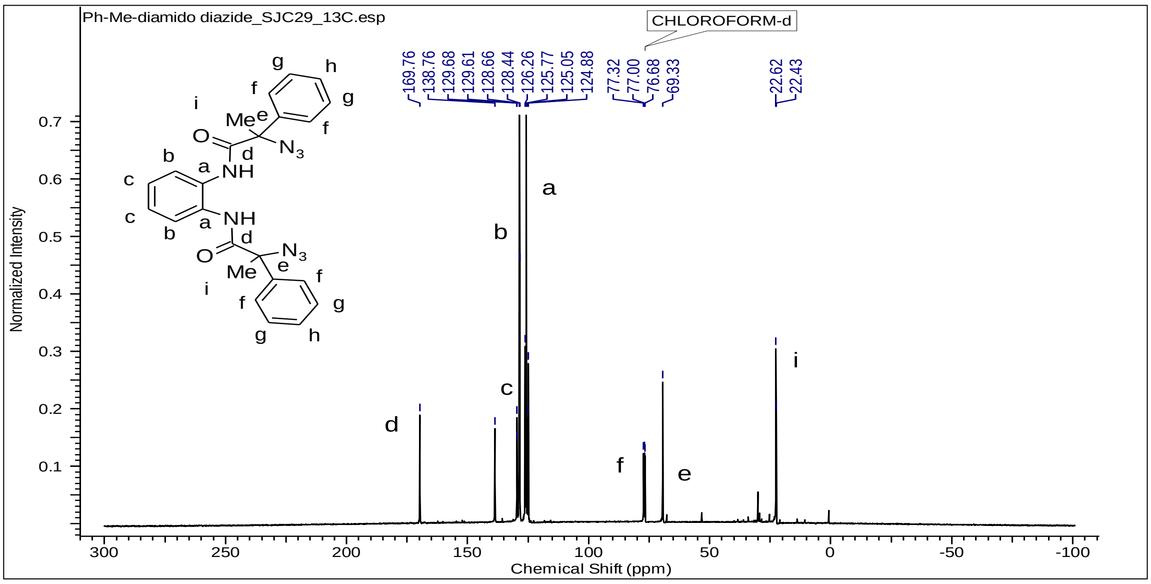

Figure S13. ^{13}C NMR of G

N,N'-(1,2-phenylene)bis(2-amino-2-phenylpropanamide) (H)

300 mg of the diamide (G) was taken a 50 mL RB and 30 mL dry methanol was added to it. Under hydrogen atmosphere (double balloon), 60-70 mg of 10% Pd/Charcoal (Sigma Aldrich) (20-25 wt%) was added. The reaction mixture was stirred under H_2 for 12 h at room temperature. After completion, the reaction mixture was filtered via frit funnel using celite. The filtrate was rota-evaporated to dryness to get pure H. Yield obtained was 85%.

^{1}H NMR (400 MHz, DMSO-d6) δ 7.60-7.26 (m, 14H), 5.02 (broad, 4H), 1.69 (s, 6H)

^{13}C NMR (400 MHz, DMSO-d6) δ 174.79, 145.41, 130.80, 128.06, 126.79, 125.63, 124.95, 124.14, 60.64, 28.06

HRMS: 403.2152 (M+H), 425.1955 (M+Na) [calc'd 403.2134 (M+H)]

****The macrocyclization and metalation procedures were same as reported for bTAML synthesis[8] (see I and J).**

^{1}H NMR:

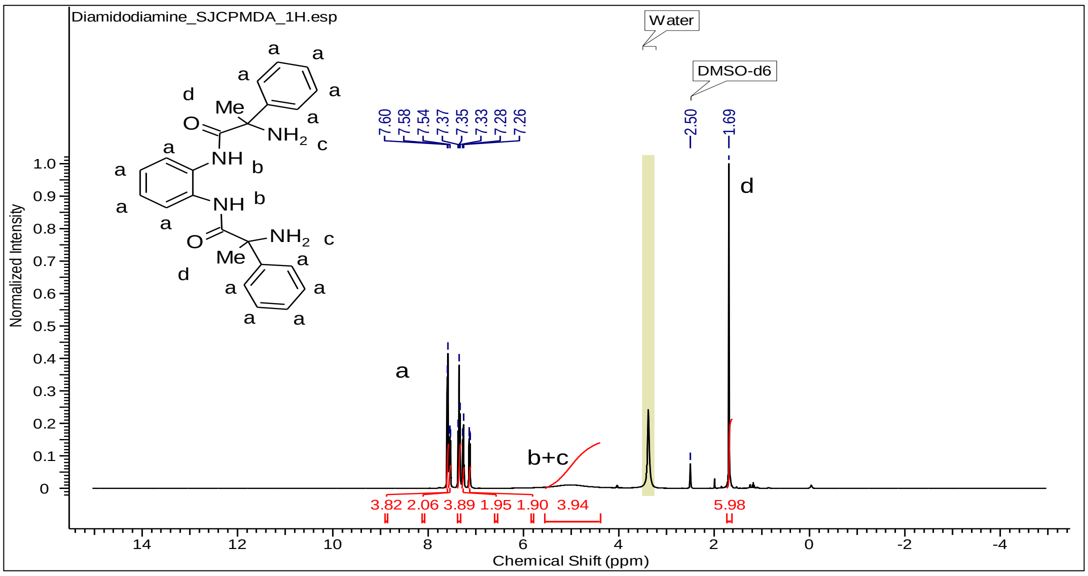

Figure S14. ^{1}H NMR of H

^{13}C NMR:

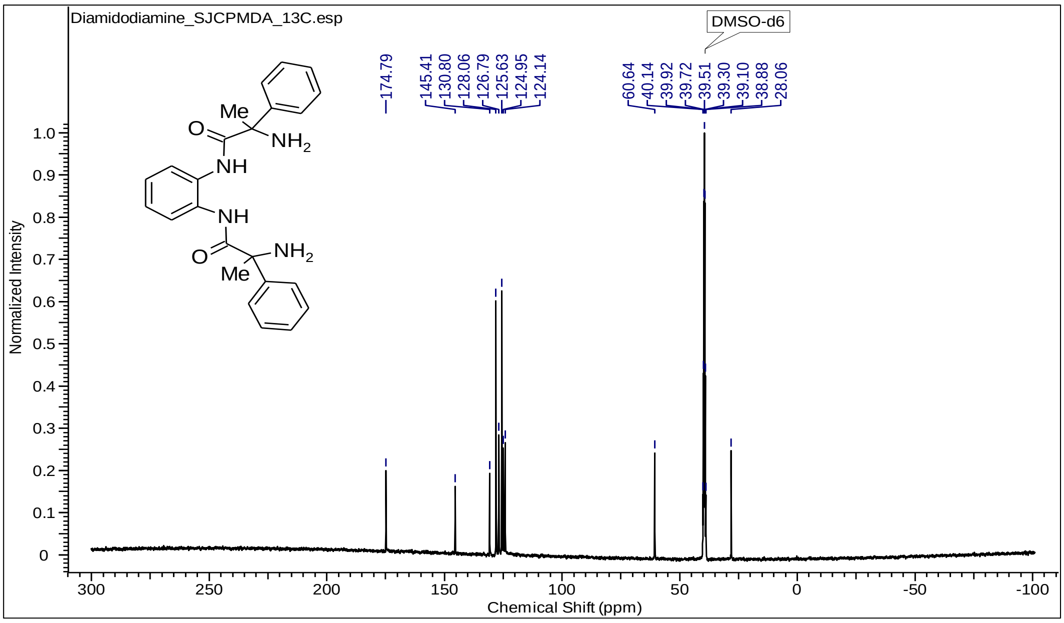

Figure S15. ^{13}C NMR of H

HRMS:

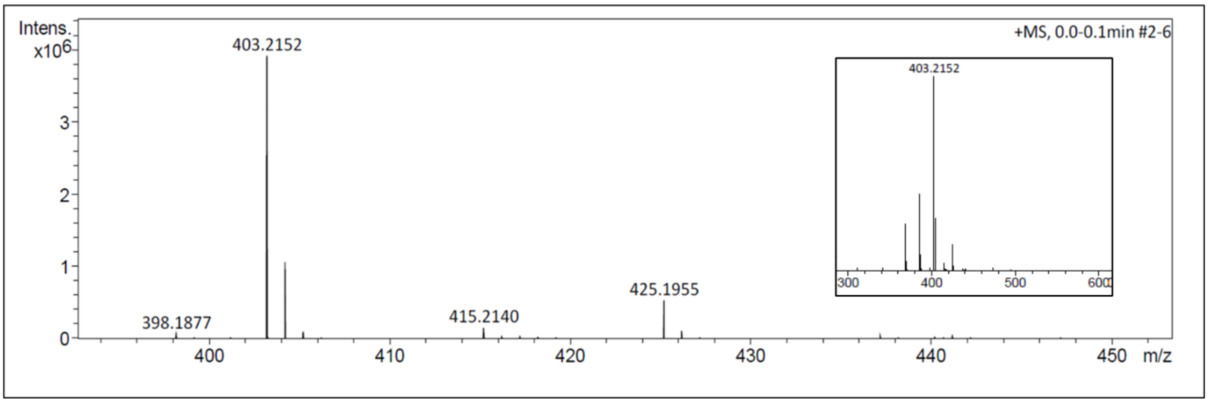

Figure S16. HRMS of H

Macrocycle (Ph,Me-bTAML) (I)

^{1}H NMR (400 MHz, $CDCl_3$) δ 8.35 (s, 2H), 7.71 (s, 2H), 7.43-7.27 (m, 14H), 2.50 (s, 3H), 1.63 (s, 3H), 1.62 (s, 3H)

^{13}C NMR (400 MHz, $CDCl_3$) δ 171.96, 157.35, 141.85, 129.47, 128.52, 126.90, 124.48, 66.38, 30.93, 26.15, Yield 29%

HRMS: 486.2136 (M+H), 508.1956 (M+Na), 524.1693 (M+K) [calc'd 485.2063 (M^+), 486.2141 (M+H)]

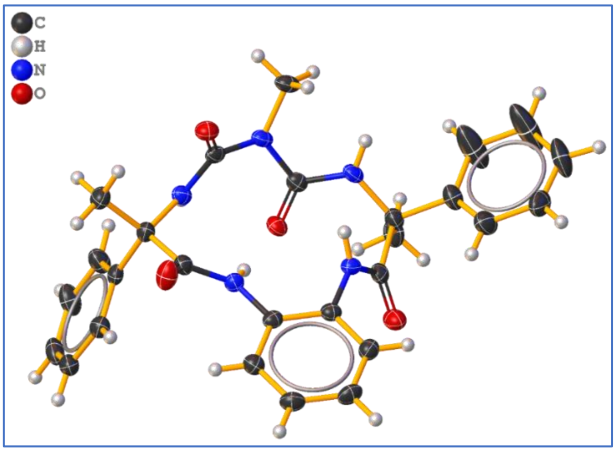

Figure S17. Model diagram of the crystal structure of the macrocycle (Ph,Me-bTAML)

Table S1a. Data collection and structure refinement parameters of macrocycle (Ph,Me-bTAML, **I**):

Identification code	2225607
Formula	$C_{27}H_{27}N_5O_4$
Formula weight	485.54

Crystal colour, habit	White
T / K	293 K
Crystal system	Monoclinic
Space group	$P2_1/c$
a/Å	11.9001(2)
b/Å	19.1517(3)
c/Å	10.5986(1)
α/°	90
β/°	102.996(2)
γ/°	90
V/Å^3	2353.63(6)
Z	4
D_c/g cm^{-3}	1.370
F(000)	1024.0
Crystal size/mm^3	0.08 × 0.06 × 0.02
Radiation	CuKα (λ = 1.54184)
2Θ range for data collection/°	8.916 to 136.56
Index ranges	$-13 \le h \le 14$, $-22 \le k \le 22$, $-12 \le l \le 12$
Reflections collected	13674
Independent reflections	4256 [R_{int} = 0.0368, R_{sigma} = 0.0363]
Data/restraints/parameters	4256/0/328
Goodness-of-fit on F^2	1.059
Final R indexes [I>=2σ (I)]	R_1 = 0.0621, wR_2 = 0.1674
Final R indexes [all data]	R_1 = 0.0704, wR_2 = 0.1758
Largest diff. peak/hole / e Å^{-3}	2.02/-0.50

$^aR_1 = \Sigma||F_o| - |F_c||/\Sigma|F_o|$. $^bwR_2 = \{\Sigma[w(|F_o|^2 - |F_c|^2)^2]/\Sigma[w(|F_o|^2)^2]\}^{1/2}$.

Table S1b. All bond lengths for **I**

**Atom1**	**Atom2**	**Length (Å)**		**Atom1**	**Atom2**	**Length (Å)**
O3	C18	1.232(2)		C3	H3	0.93
O2	C16	1.228(3)		C3	C4	1.383(3)
O1	C7	1.219(3)		C19	C20	1.534(3)
O4	C27	1.224(3)		C10	C11	1.361(4)
N5	H5	0.86		C10	C15	1.393(6)
N5	C1	1.422(3)		C4	H4A	0.93
N5	C27	1.362(3)		C17	H17A	0.96
N1	H1	0.86		C17	H17B	0.96
N1	C6	1.428(3)		C17	H17C	0.96
N1	C7	1.357(3)		C26	H26	0.93

N4	H4	0.86		C26	C25	1.386(4)
N4	C18	1.349(3)		C20	H20A	0.96
N4	C19	1.478(3)		C20	H20B	0.96
N2	H2	0.86		C20	H20C	0.96
N2	C16	1.354(3)		C9	H9A	0.96
N2	C8	1.476(3)		C9	H9B	0.96
N3	C18	1.416(3)		C9	H9C	0.96
N3	C16	1.415(3)		C22	H22	0.931
N3	C17	1.466(3)		C22	C23	1.390(4)
C1	C6	1.401(3)		C25	H25	0.931
C1	C2	1.395(3)		C25	C24	1.382(4)
C6	C5	1.383(3)		C24	H24	0.93
C7	C8	1.550(4)		C24	C23	1.371(5)
C21	C19	1.534(3)		C23	H23	0.93
C21	C26	1.389(4)		C11	H11	0.93
C21	C22	1.386(4)		C11	C12	1.391(4)
C2	H2A	0.93		C12	H12	0.93
C2	C3	1.386(4)		C12	C13	1.354(5)
C27	C19	1.555(3)		C13	H13	0.93
C5	H5A	0.93		C13	C14	1.400(6)
C5	C4	1.391(3)		C15	H15	0.929
C8	C10	1.534(4)		C15	C14	1.384(6)
C8	C9	1.534(3)		C14	H14	0.93

Table S1c. All bond angles for **I**

**Atom1**	**Atom2**	**Atom3**	**Angle/°**		**Atom1**	**Atom2**	**Atom3**	**Angle/°**
H5	N5	C1	117.2		C27	C19	C20	110.3(2)
H5	N5	C27	117.2		C8	C10	C11	123.3(2)
C1	N5	C27	125.7(2)		C8	C10	C15	118.5(3)

H1	N1	C6	118.3		C11	C10	C15	118.1(3)
H1	N1	C7	118.4		C5	C4	C3	119.6(2)
C6	N1	C7	123.3(2)		C5	C4	H4A	120.2
H4	N4	C18	118.8		C3	C4	H4A	120.3
H4	N4	C19	118.8		N3	C17	H17A	109.5
C18	N4	C19	122.5(2)		N3	C17	H17B	109.4
H2	N2	C16	119.5		N3	C17	H17C	109.5
H2	N2	C8	119.5		H17A	C17	H17B	109.5
C16	N2	C8	121.1(2)		H17A	C17	H17C	109.5
C18	N3	C16	117.3(2)		H17B	C17	H17C	109.5
C18	N3	C17	114.6(2)		C21	C26	H26	119.6
C16	N3	C17	119.7(2)		C21	C26	C25	120.8(2)
N5	C1	C6	119.0(2)		H26	C26	C25	119.6
N5	C1	C2	121.4(2)		C19	C20	H20A	109.5
C6	C1	C2	119.5(2)		C19	C20	H20B	109.5
O3	C18	N4	124.4(2)		C19	C20	H20C	109.5
O3	C18	N3	119.8(2)		H20A	C20	H20B	109.5
N4	C18	N3	115.8(2)		H20A	C20	H20C	109.5
N1	C6	C1	120.0(2)		H20B	C20	H20C	109.5
N1	C6	C5	120.2(2)		C8	C9	H9A	109.4
C1	C6	C5	119.8(2)		C8	C9	H9B	109.5
O1	C7	N1	123.4(2)		C8	C9	H9C	109.4
O1	C7	C8	120.2(2)		H9A	C9	H9B	109.5
N1	C7	C8	116.4(2)		H9A	C9	H9C	109.5
C19	C21	C26	119.8(2)		H9B	C9	H9C	109.5
C19	C21	C22	121.8(2)		C21	C22	H22	119.6
C26	C21	C22	118.2(2)		C21	C22	C23	120.8(3)
O2	C16	N2	122.9(2)		H22	C22	C23	119.6
O2	C16	N3	121.8(2)		C26	C25	H25	119.9

N2	C16	N3	115.2(2)		C26	C25	C24	120.2(2)
C1	C2	H2A	120		H25	C25	C24	119.9
C1	C2	C3	120.0(2)		C25	C24	H24	120.3
H2A	C2	C3	120		C25	C24	C23	119.5(3)
O4	C27	N5	123.8(2)		H24	C24	C23	120.2
O4	C27	C19	121.1(2)		C22	C23	C24	120.3(3)
N5	C27	C19	115.0(2)		C22	C23	H23	119.9
C6	C5	H5A	119.7		C24	C23	H23	119.8
C6	C5	C4	120.6(2)		C10	C11	H11	119.3
H5A	C5	C4	119.7		C10	C11	C12	121.4(3)
N2	C8	C7	110.7(2)		H11	C11	C12	119.3
N2	C8	C10	107.3(2)		C11	C12	H12	119.6
N2	C8	C9	110.5(2)		C11	C12	C13	120.8(3)
C7	C8	C10	109.9(2)		H12	C12	C13	119.6
C7	C8	C9	108.4(2)		C12	C13	H13	120.8
C10	C8	C9	109.9(2)		C12	C13	C14	118.4(3)
C2	C3	H3	119.7		H13	C13	C14	120.8
C2	C3	C4	120.6(2)		C10	C15	H15	119.9
H3	C3	C4	119.7		C10	C15	C14	120.3(4)
N4	C19	C21	108.5(2)		H15	C15	C14	119.9
N4	C19	C27	109.5(2)		C13	C14	C15	120.3(4)
N4	C19	C20	112.0(2)		C13	C14	H14	119.8
C21	C19	C27	108.7(2)		C15	C14	H14	119.9
C21	C19	C20	107.7(2)					

Chiral HPLC of I:

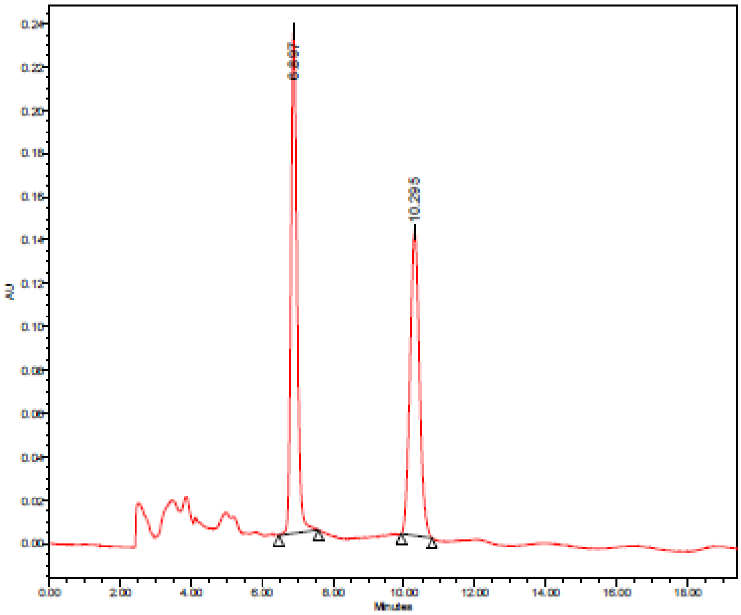

Retention Time (min)	*Area*	*% Area*	*Height*
6.897	*2718185*	*51.27*	*231446*
10.295	*2583967*	*48.73*	*139738*

Figure S18. Chiral HPLC of macrocycle; indicating the presence of racemic mixture (achiral)

^{1}H NMR:

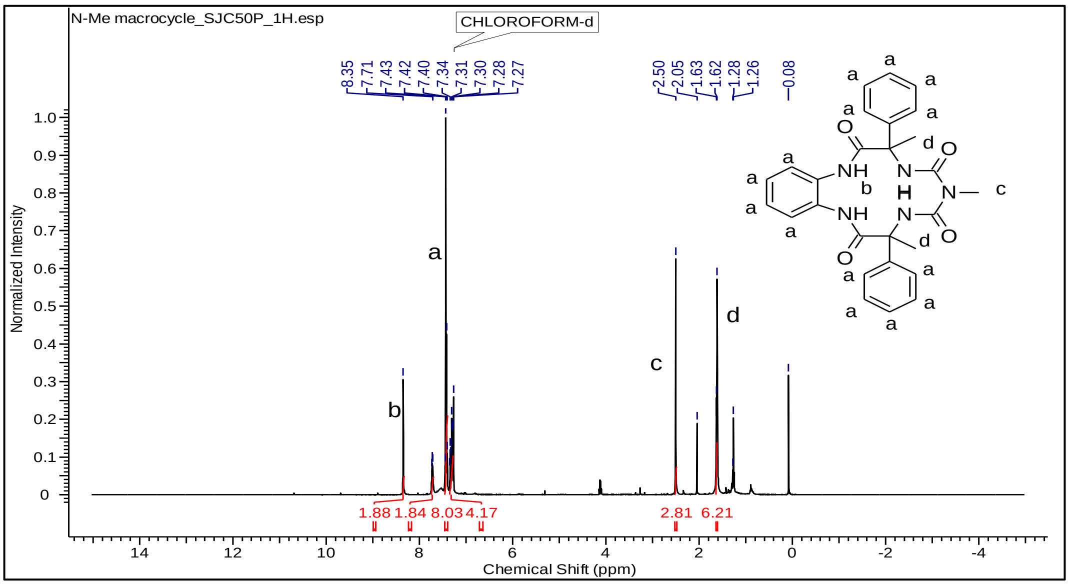

Figure S19. ^{1}H NMR of I

^{13}C NMR:

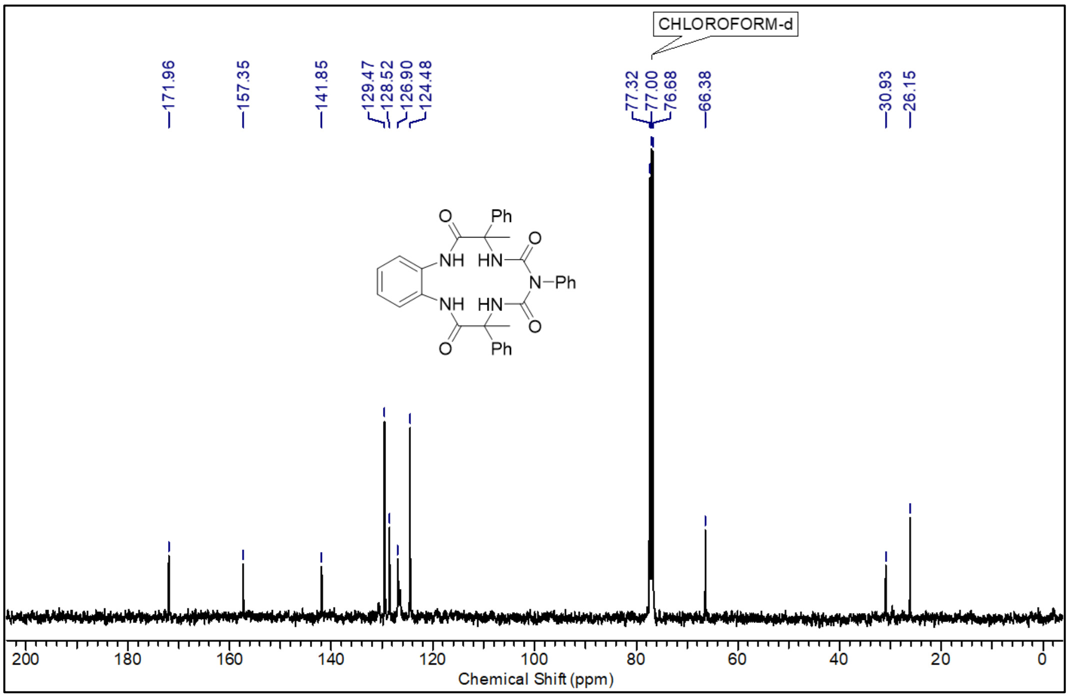

Figure S20. ^{13}C NMR of I

HRMS:

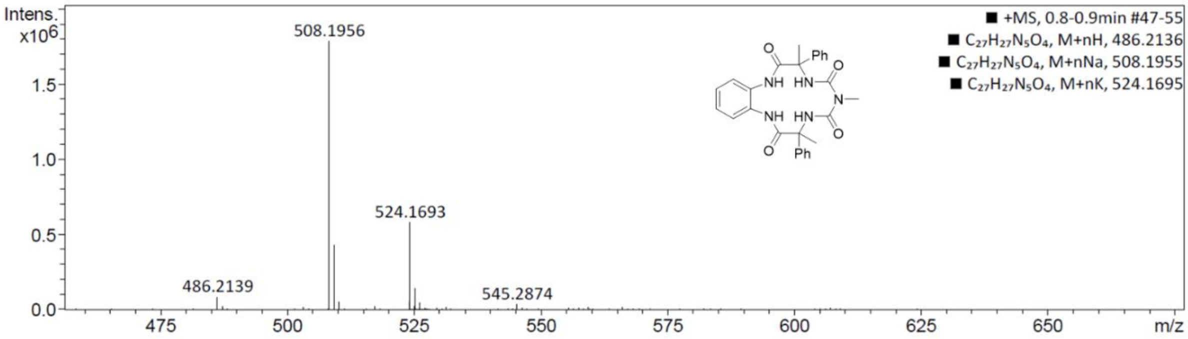

Figure S21. HRMS of I

Fe^{III}(Ph,Me-bTAML)Cl (J, complex **2)**

NMR inactive (paramagnetic)

HRMS: 537.1182 (m/z) [calc'd 537.1099 (m/z)]

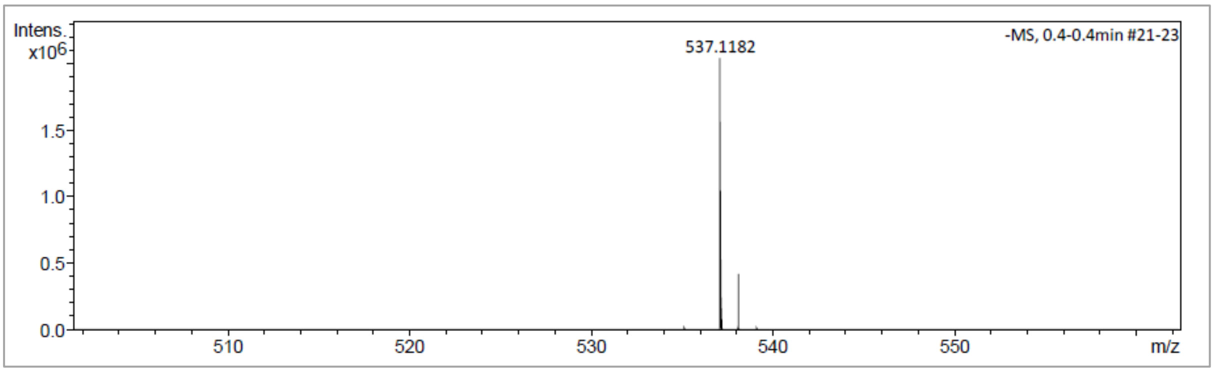

Figure S22. HRMS of complex **2**

Single Crystal X-ray Diffraction (SCXRD):

CCDC number: 2132158

The X-ray crystal structure of **2** indicates a square-pyramidal Fe(III) with an axial Cl atom (Fig. S1), resulting from an orthorhombic crystal system (space group, *Pbca* (no. 61)). All the four Fe–N bond lengths are around 1.884 Å and the Fe(III) lies 0.45 Å above the plane formed by the four donor nitrogens (N2, N3, N4, N5). The N1 atom of the Me-biuret ring is 3.141 Å away from the Fe(III) indicating that this N-atom is not involved in bonding with the Fe(III) center. The Me-biuret ring is close to planarity and the torsion angle between the C1–N1 and C2–N2 planes is around 174°. Therefore, the N5 atom in the six-membered ring is strictly sp^2 hybridized and the nitrogen lone pair, residing in the p orbital of N1 atom, is conjugated extensively to the carbonyl C-atom on both sides.

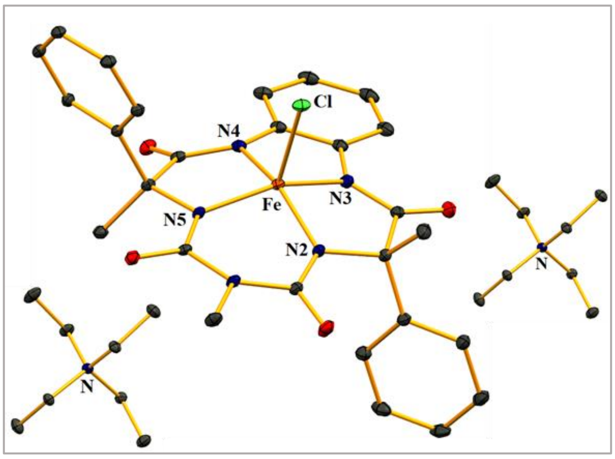

Figure S23. ORTEP diagram of **[Fe^{III}(Ph,Me-bTAML)Cl](Et_4N)$_2$ (2)** with 50% probability

Table S2a. Some important bond lengths and bond angles of the crystal structure of **2**

Bond length (Å)		Bond angles (°)			
Fe–N2	1.8793(9)	N2–Fe–N3	84.36(4)	N3–Fe–N5	151.64(4)
Fe–N3	1.8810(9)	N2–Fe–N5	94.90(4)	N2–Fe–Cl	100.21(3)

Fe–N4	1.8915(9)	N3–Fe–N4	83.24(4)	N3–Fe–Cl	101.03(3)
Fe–N5	1.8850(9)	N4–Fe–N5	84.59(4)	N4–Fe–Cl	106.54(3)
Fe–Cl	2.3606(3)	N4–Fe–N2	152.20(4)	N5–Fe–Cl	106.97(3)

Table S2b. Data collection and structure refinement parameters of **2**:

Identification number	2132158
Formula	$C_{43}H_{63}ClFeN_7O_4$
Formula weight	833.30
Crystal colour, habit	Orange-red
T / K	100(2)
Crystal system	Orthorhombic
Space group	*Pbca* (no. 61)
a/Å	13.9731(5)
b/Å	16.3251(5)
c/Å	37.9097(13)
α/º	90
β/º	90
γ/º	90
V/Å^3	8647.7(5)
Z	8
D_c/g cm^{-3}	1.280
μ/mm^{-1}	0.460
Reflections measured	160929
Unique reflections/R_{int}	13215 /R_{int} = 0.0526,
Reflections used [$I > 2\sigma(I)$]	12290
R_1^a, wR_2^b [$I > 2\sigma(I)$]	R_1= 0.0355^a, wR_2= 0.0911^b
R_1^a, wR_2^b (all data)	R_1=0.0389^a, wR_2= 0.0929^b
GOF on F^2	1.082

$^aR_1 = \Sigma||F_o| - |F_c||/\Sigma|F_o|$. $^bwR_2 = \{\Sigma[w(|F_o|^2 - |F_c|^2)^2]/\Sigma[w(|F_o|^2)^2]\}^{1/2}$.

Table S2c. All bond lengths for crystal structure of **2**

**Atom**	**Atom**	**Length/Å**		**Atom**	**Atom**	**Length/Å**
Fe1	Cl1	2.3606(3)		C26	C25	1.3951(15)
Fe1	N5	1.8850(9)		C24	C23	1.3891(17)
Fe1	N3	1.8810(9)		C24	C25	1.3920(16)
Fe1	N4	1.8915(9)		C38	C39	1.5158(17)
Fe1	N2	1.8793(9)		C21	C19	1.5388(14)
O3	C18	1.2345(13)		C21	C22	1.4024(14)
O4	C27	1.2404(13)		C23	C22	1.3964(15)
O2	C11	1.2299(14)		C19	C18	1.5591(15)
O1	C2	1.2402(13)		C19	C20	1.5377(15)
N6	C30	1.5195(14)		C12	C17	1.4166(16)

N6	C28	1.5205(14)		C12	C13	1.3964(15)
N6	C32	1.5219(14)		C30	C31	1.5149(18)
N6	C34	1.5219(14)		C40	C41	1.5149(17)
N5	C27	1.3460(13)		C28	C29	1.5128(16)
N5	C19	1.4819(13)		C32	C33	1.5095(17)
N7	C42	1.5188(14)		C16	C17	1.3968(15)
N7	C38	1.5287(15)		C16	C15	1.4004(17)
N7	C40	1.5192(15)		C14	C13	1.4005(18)
N7	C36	1.5159(15)		C14	C15	1.380(2)
N1	C27	1.4243(13)		C34	C35	1.5080(16)
N1	C1	1.4675(14)		C6	C5	1.3971(15)
N1	C2	1.4267(14)		C6	C7	1.3994(17)
N3	C12	1.4085(14)		C11	C3	1.5613(16)
N3	C11	1.3495(14)		C3	C5	1.5303(15)
N4	C18	1.3525(14)		C3	C4	1.5339(16)
N4	C17	1.4147(13)		C5	C10	1.3929(16)
N2	C2	1.3419(14)		C7	C8	1.381(2)
N2	C3	1.4851(14)		C8	C9	1.386(2)
C42	C43	1.5212(16)		C10	C9	1.3922(16)
C26	C21	1.3945(15)		C36	C37	1.5227(19)

Table S2d. All bond angles for crystal structure of **2**

**Atom**	**Atom**	**Atom**	**Angle/°**		**Atom**	**Atom**	**Atom**	**Angle/°**
N5	Fe1	Cl1	106.97(3)		N5	C19	C21	113.03(8)
N5	Fe1	N4	84.59(4)		N5	C19	C18	106.13(8)
N3	Fe1	Cl1	101.03(3)		N5	C19	C20	113.01(8)
N3	Fe1	N5	151.64(4)		C21	C19	C18	106.14(8)
N3	Fe1	N4	83.24(4)		C20	C19	C21	111.03(8)
N4	Fe1	Cl1	106.54(3)		C20	C19	C18	106.94(9)
N2	Fe1	Cl1	100.21(3)		N3	C12	C17	112.55(9)
N2	Fe1	N5	94.90(4)		C13	C12	N3	127.47(11)
N2	Fe1	N3	84.36(4)		C13	C12	C17	119.96(11)
N2	Fe1	N4	152.20(4)		C24	C25	C26	120.56(11)
C30	N6	C28	111.96(9)		C31	C30	N6	115.48(10)
C30	N6	C32	112.15(9)		C41	C40	N7	114.79(9)
C30	N6	C34	104.73(8)		C29	C28	N6	114.84(9)
C28	N6	C32	105.19(8)		O1	C2	N1	117.37(10)
C28	N6	C34	111.76(8)		O1	C2	N2	124.60(10)
C34	N6	C32	111.22(9)		N2	C2	N1	118.04(9)
C27	N5	Fe1	125.34(7)		O3	C18	N4	126.56(10)
C27	N5	C19	118.68(9)		O3	C18	C19	121.10(10)
C19	N5	Fe1	115.38(6)		N4	C18	C19	112.33(9)
C42	N7	C38	107.40(9)		C33	C32	N6	115.11(10)
C42	N7	C40	108.90(8)		C17	C16	C15	119.18(12)
C40	N7	C38	111.28(9)		C15	C14	C13	121.29(11)
C36	N7	C42	111.39(9)		N4	C17	C12	112.78(9)
C36	N7	C38	108.81(9)		C16	C17	N4	126.99(11)

C36	N7	C40	109.06(9)		C16	C17	C12	120.23(10)
C27	N1	C1	113.97(9)		C35	C34	N6	115.43(9)
C27	N1	C2	130.61(9)		C12	C13	C14	118.90(12)
C2	N1	C1	114.87(9)		C5	C6	C7	120.55(12)
C12	N3	Fe1	115.34(7)		O2	C11	N3	127.04(11)
C11	N3	Fe1	117.82(8)		O2	C11	C3	120.60(10)
C11	N3	C12	126.83(10)		N3	C11	C3	112.36(9)
C18	N4	Fe1	117.30(7)		C14	C15	C16	120.43(11)
C18	N4	C17	124.22(9)		N2	C3	C11	107.38(9)
C17	N4	Fe1	114.83(7)		N2	C3	C5	113.01(9)
C2	N2	Fe1	126.39(7)		N2	C3	C4	110.61(9)
C2	N2	C3	118.10(9)		C5	C3	C11	104.16(9)
C3	N2	Fe1	114.86(7)		C5	C3	C4	113.67(9)
O4	C27	N5	124.98(10)		C4	C3	C11	107.46(9)
O4	C27	N1	117.02(9)		C23	C22	C21	120.76(10)
N5	C27	N1	118.00(9)		C6	C5	C3	121.97(10)
N7	C42	C43	114.13(9)		C10	C5	C6	118.33(11)
C21	C26	C25	120.75(10)		C10	C5	C3	119.37(10)
C23	C24	C25	119.18(10)		C8	C7	C6	120.21(12)
C39	C38	N7	114.99(10)		C7	C8	C9	119.81(11)
C26	C21	C19	121.94(9)		C9	C10	C5	121.05(12)
C26	C21	C22	118.32(9)		N7	C36	C37	114.45(10)
C22	C21	C19	119.71(9)		C8	C9	C10	120.03(12)
C24	C23	C22	120.37(10)					

EPR Spectroscopy:

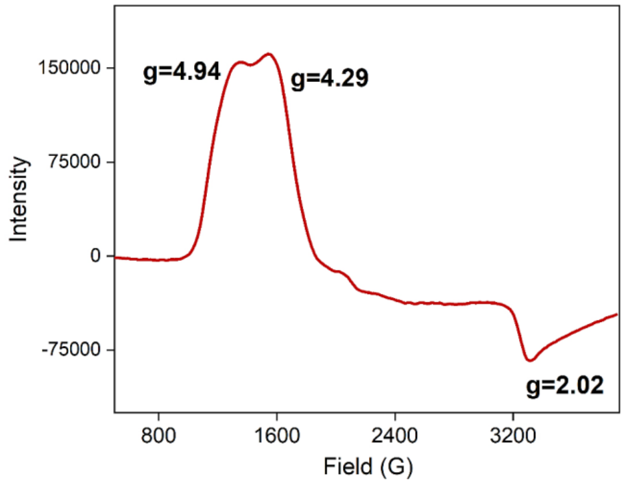

Figure S24. EPR of **2** in acetonitrile (7.5 mM) at 100 K.

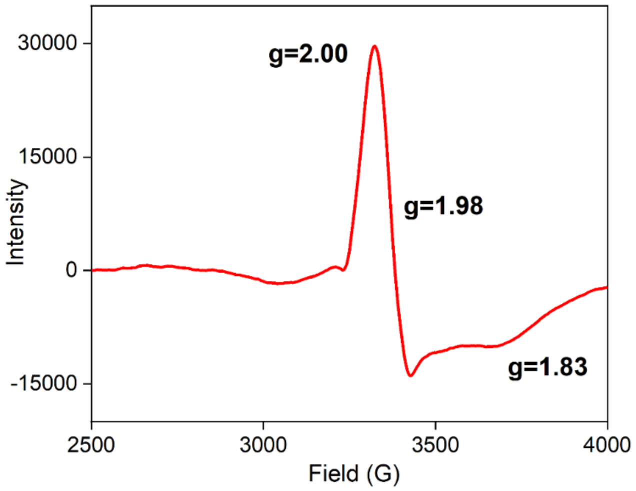

Figure S25. EPR of **3** in acetonitrile (3 mM) at 100 K; the g-values indicate the formation of S = ½ species [i.e., $Fe^{V}(O)$]

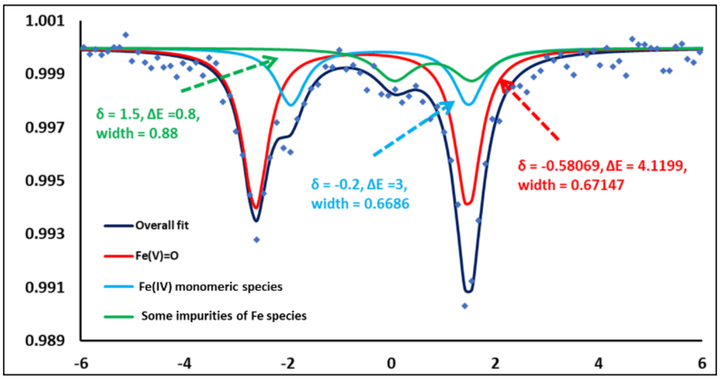

Figure S26. Mössbauer spectrum of **3** (2 mM) at 77K [61% $Fe^{V}(O)$ with 23% Fe(IV) and 16% some impurities of Fe species].

Electrochemistry:

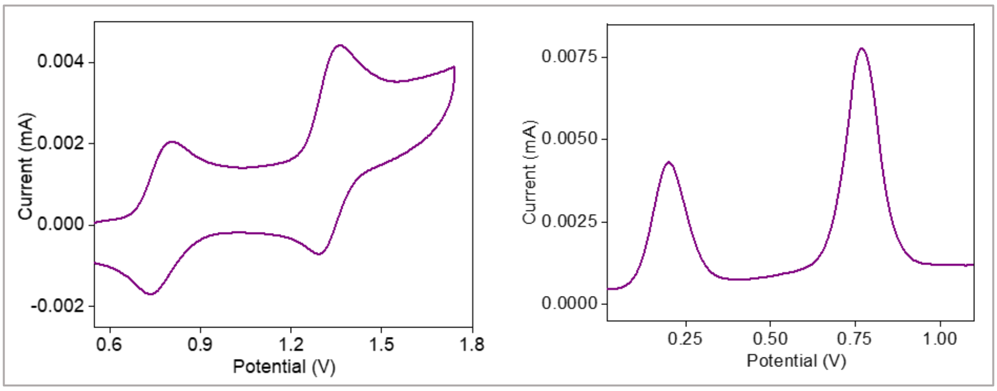

Figure S27. CV and DPV of **2** in acetonitrile solvent (0.1 mM); reduction potential values are plotted with respect to NHE

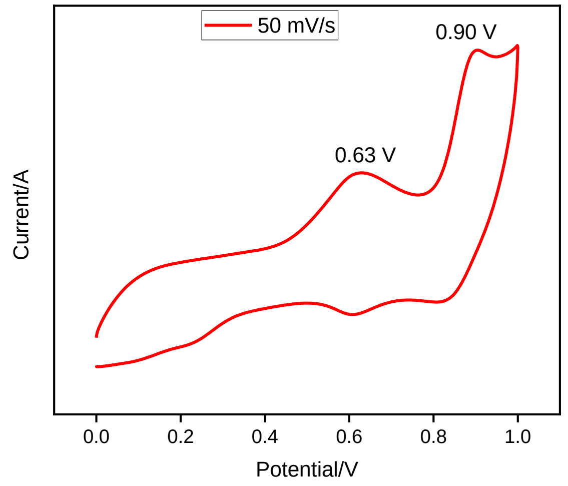

Figure S28. CV **2** in 100% water solvent (0.1 mM); reduction potential values are plotted with respect to Ag/AgCl (3M KCl) electrode.

3C. Reactivity Studies

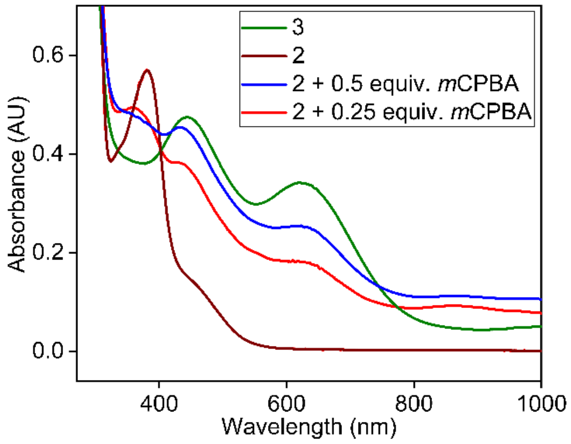

Figure S29. **Red spectrum:** Addition of 0.25 equiv *m*CPBA to **2** in MeCN (λ_{max}: 434 nm and 625 nm); **Blue spectrum:** Addition of 0.5 equiv *m*CPBA to **2** in MeCN (λ_{max}: 434 nm and 625 nm); Formation of a new species; Same spectrum was obtained when comproportionation reaction was performed between equimolar amounts of **2** and **3**.

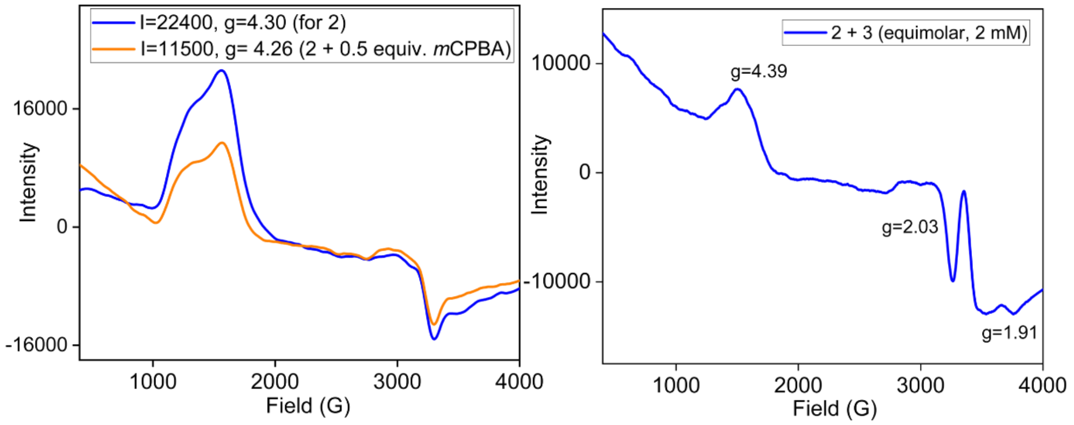

Figure S30. Left: EPR of **2** and taken with 0.5 equiv *m*CPBA; Right: EPR of equimolar mixture of **2** and **3**

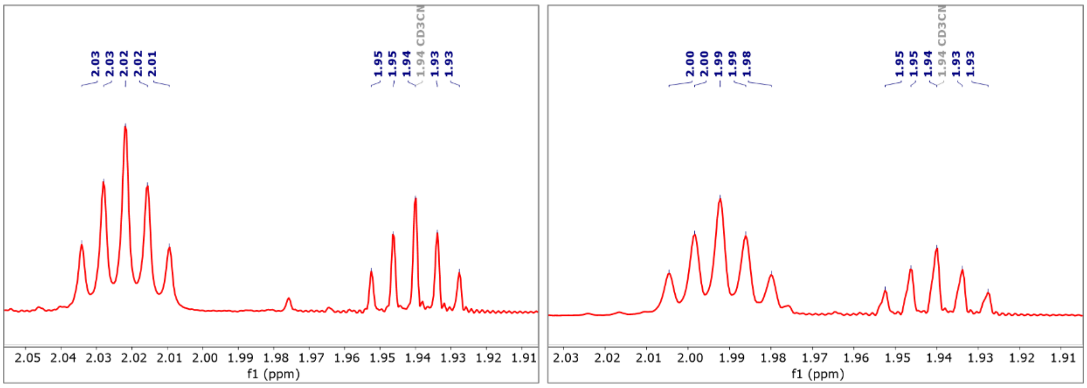

Figure S31. **Left:** Paramagnetic NMR: **2** in CD_3CN (2.4 mM); no. of unpaired electrons: 3 (μ = 3.9), (expt) = 4.3; **Right:** Paramagnetic NMR: **2** + 0.5 equiv NaOCl (2.4 mM): (expt) = 3.4, no. of unpaired electrons ≈ 2.5, indicates a decrease in Fe(III) concentration

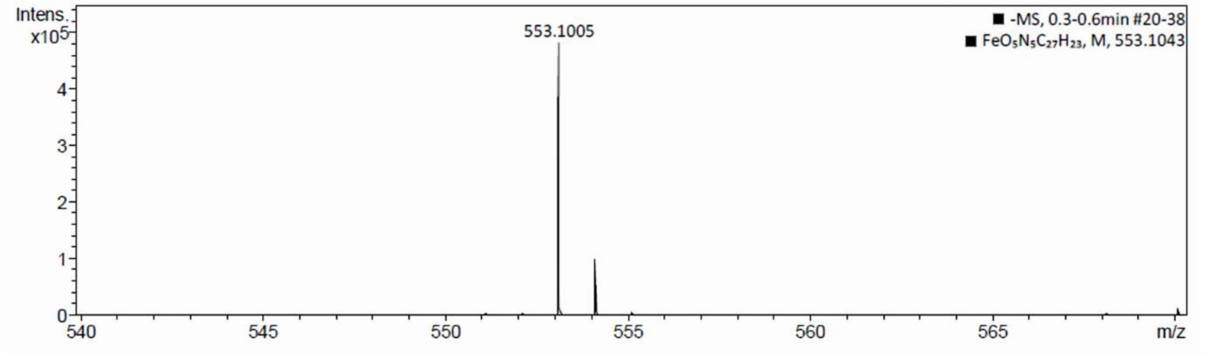

Figure S32. HRMS of **3** formed upon addition of *m*CPBA to **2** in CH_3CN; Experimental m/z (M^-) 553.1005 (100%); calc'd m/z (M^-) 553.1049 (100%).

3D. Kinetics Studies

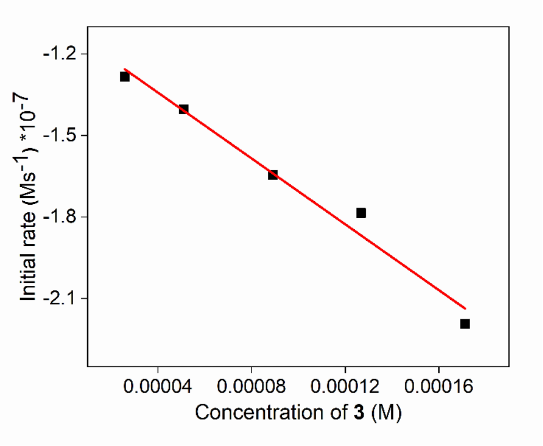

Figure S33. Self decay of **3** (prepared from mCPBA) in CH_3CN solvent at 298 K (k_{decay} 6.0531 x 10^{-4} s^{-1}).

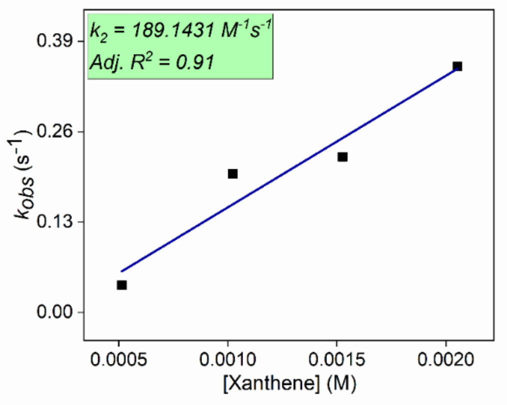

Figure S34. Plot of k_{obs} vs concentration of xanthene (k_2 = 189.1431 ± 33.975 $M^{-1}s^{-1}$).

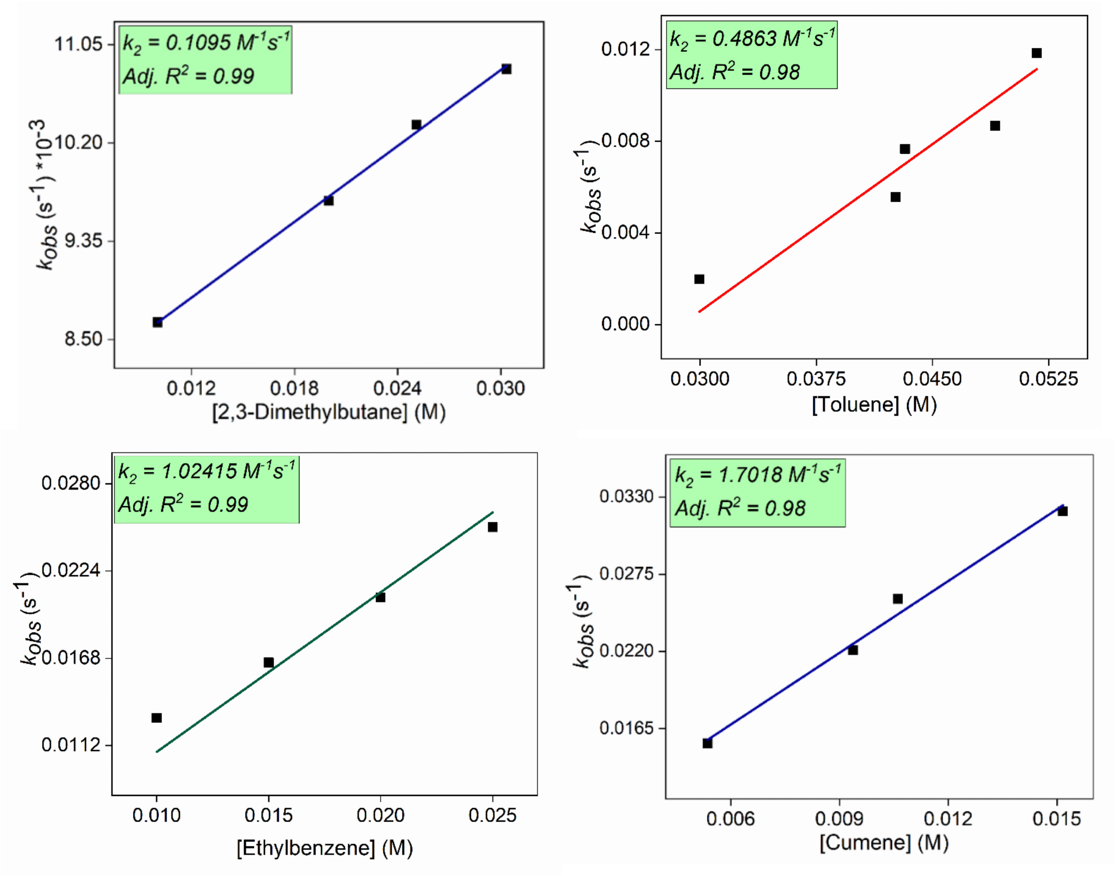

Figure S35. Plot of k_{obs} vs concentration of 2,3-dimethylbutane (k_2 = 0.1095 ± 0.004 $M^{-1}s^{-1}$), toluene (k_2 = 0.4863 ± 0.012 $M^{-1}s^{-1}$), ethylbenzene (k_2 = 1.0242 ± 0.039 $M^{-1}s^{-1}$), cumene (k_2 = 1.7018 ± 0.130 $M^{-1}s^{-1}$).

Table S3. Second order rate constants (k_2) for oxidation of model sp^3 C–H bond-containing substrates in CH_3CN; comparison of **3** with **1A′**.

**Substrates**	**BDE(C-H) (kcal/mol)**	**k_2 ($M^{-1}s^{-1}$) with Fe(*PhMeb*TAML)-Cl complex**	**k_2 ($M^{-1}s^{-1}$) with Fe(bTAML)-Cl complex**
Xanthene	75.5	189.1431 ± 33.975	-
Cumene	84.5	1.7018 ± 0.130	0.791 ± 0.090
Ethylbenzene	87	1.0242 ± 0.039	0.284 ± 0.021
Toluene	90	0.4863 ± 0.012	0.136 ± 0.016
2,3-Dimethylbutane	96.5	0.1095 ± 0.004	0.0350 ± 0.010

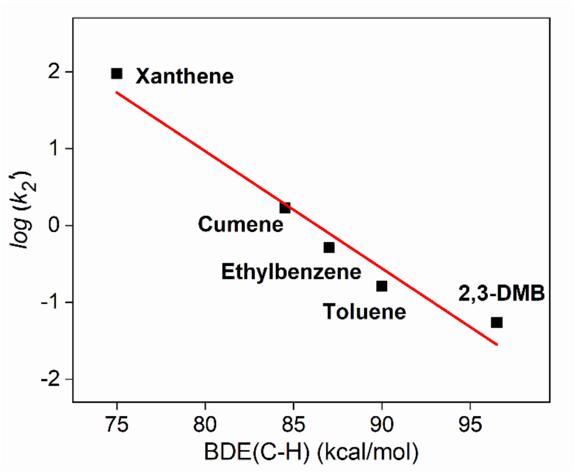

Figure S36. BEP curve for **3** (by *m*CPBA) catalyzed C–H bond oxidation at single turnover conditions in CH_3CN. A linear plot (slope 0.1525 ± 0.0170 $M^{-1}s^{-1}$) was observed.

3E. Oxoiron(V) intermediate formation by *m*CPBA treatment in 100% water and its kinetic study

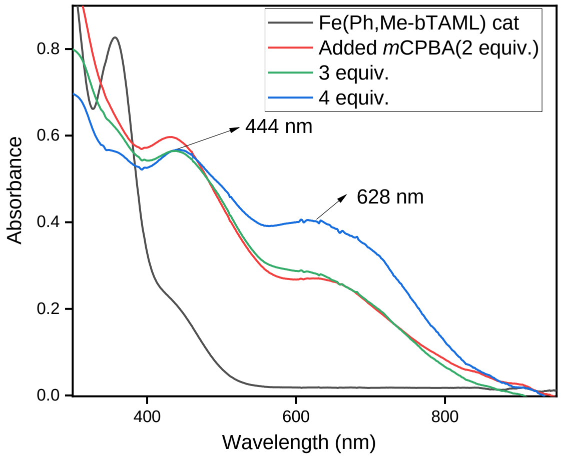

Figure S37. Qualitative formation of $Fe^{V}(O)$ from complex **2** with the addition of *m*CPBA of different equivalents.

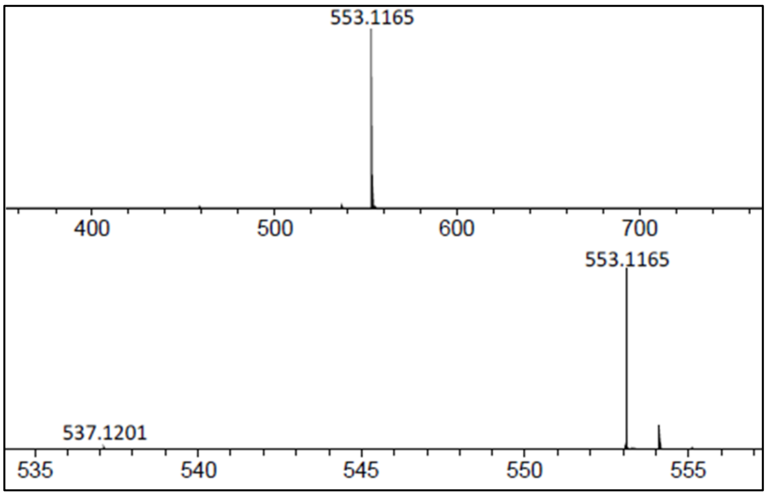

Figure S38. HRMS data of **3** formed by *m*CPBA in 100% water; Experimental m/z (M^-) 553.1165 (100%); calc'd m/z (M^-) 553.1049.

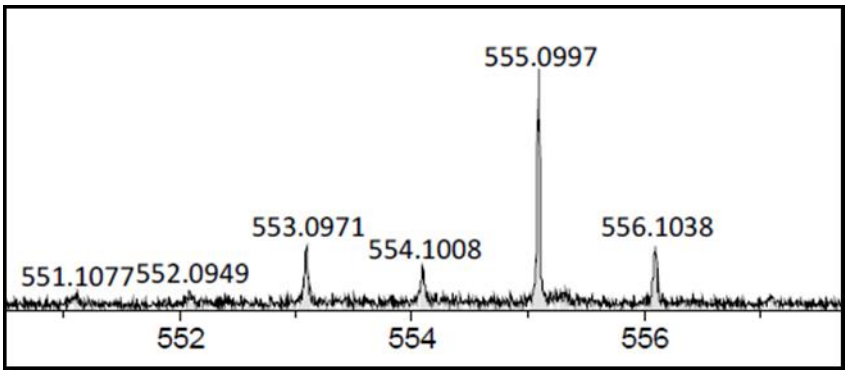

Figure S39. HRMS of **3** (with *m*CPBA) in 100% water in presence of H_2O^{18}. Calculated m/z of **2** after O^{18} incorporation = 555.0997.

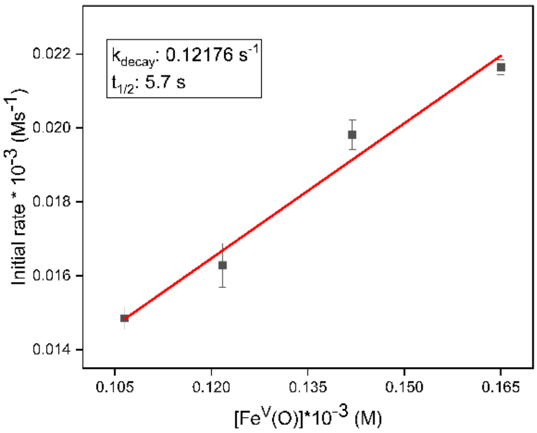

Figure S40. Self decay of complex **3** (prepared from *m*CPBA) in 100% water at 298 K (k_{decay} 0.12176 s^{-1})

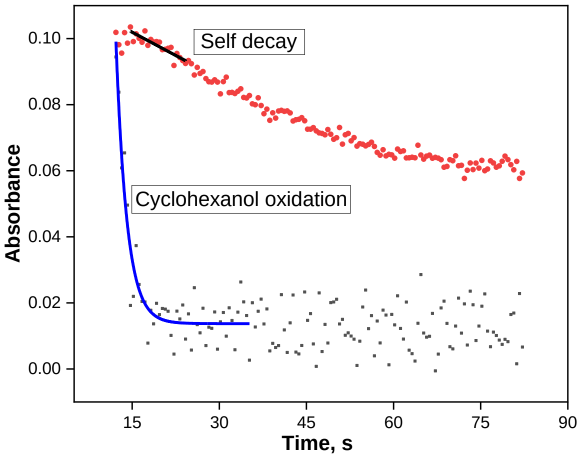

Figure S41. Comparison rate data of self decay and cyclohexanol oxidation of complex **3** in 100% water at 298 K.

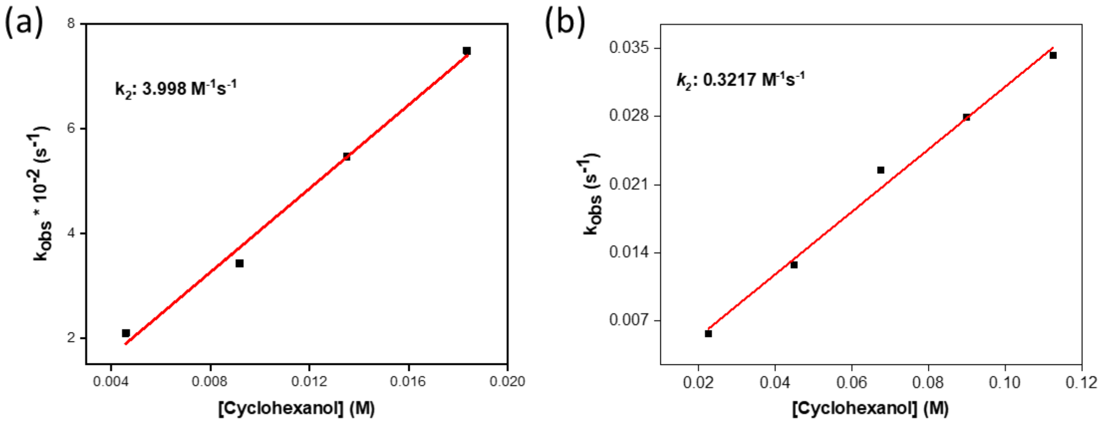

Figure S42. Determination of second order rate constant values of complex **3** for cyclohexanol oxidation in (a) 80% CH_3CN-H_2O and (b) 97% CH_3CN-H_2O medium with *m*CPBA.

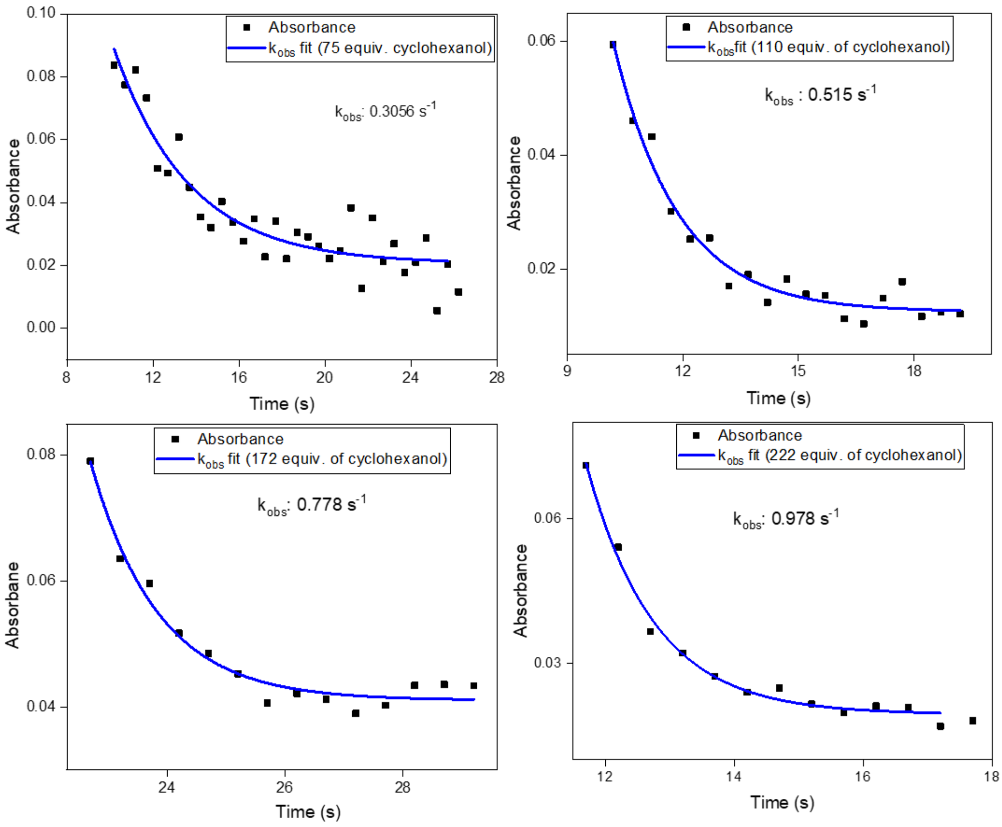

Figure S43. Kinetic traces for the reaction of complex **3** (0.045 mM) in 100% water by *m*CPBA with cyclohexanol at various concentrations at 10°C. The kinetic traces were fitted to the equation, $[(A_t = A_\alpha - (A_\alpha - A_o)e(^{-k_{obs}t})]$ for obtaining k_{obs} values.

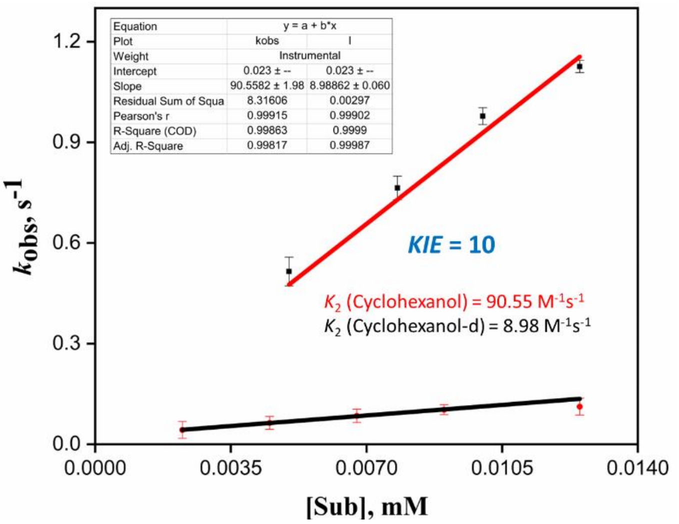

Figure S44. Plot of k_{obs} / s^{-1} vs [cyclohexanol] (k_2: 90.55 $M^{-1}s^{-1}$) (red line) and [cyclohexanol-*d*] (k_2: 8.98 $M^{-1}s^{-1}$) (black line) by 3 formed by *m*CPBA in water medium showing significant kinetic isotope effect at 10 °C.

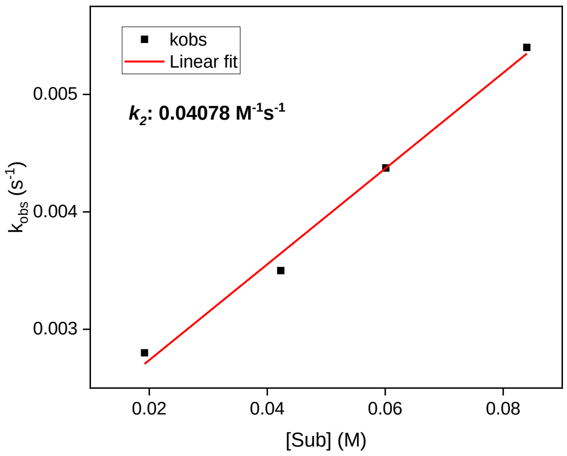

Figure S45. Determination of second order rate constant values of complex **3** for 6-methylheptan-2-yl isonicotinate oxidation in 97% CH_3CN-H_2O medium with *m*CPBA.

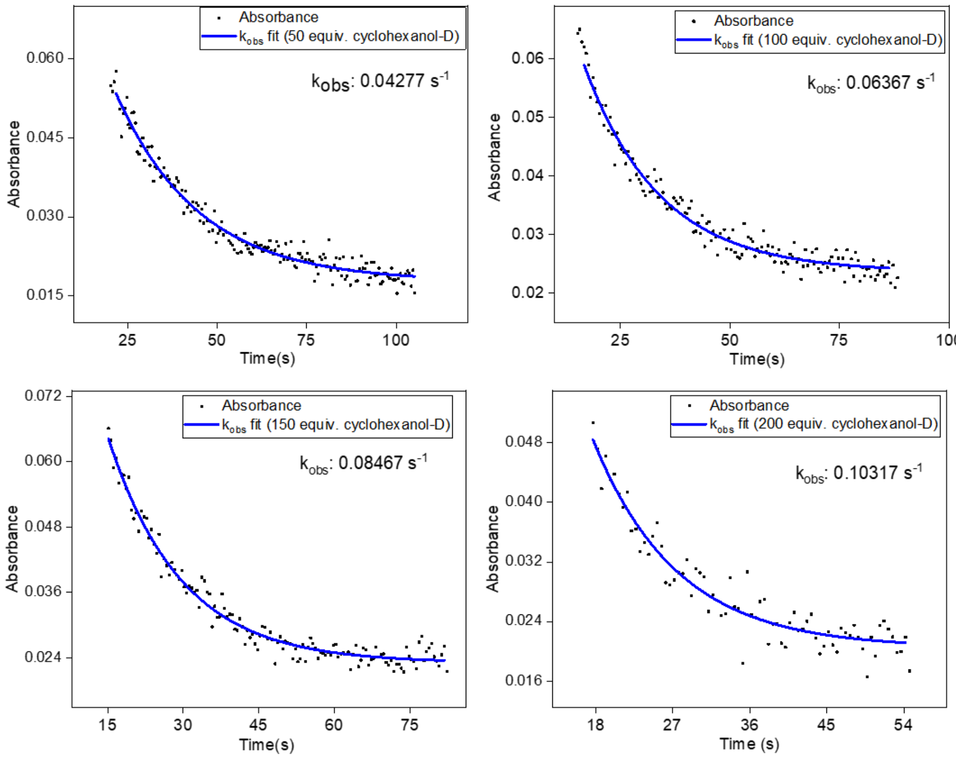

Figure S46. Kinetic traces for the reaction of complex **3** (0.045 mM) in 100% water by *m*CPBA with cyclohexanol-*d* at various concentrations. The kinetic traces were fitted to the equation, $[(A_t = A_\alpha - (A_\alpha - A_o)e(^{-k_{obs}t})]$ for obtaining k_{obs} values.

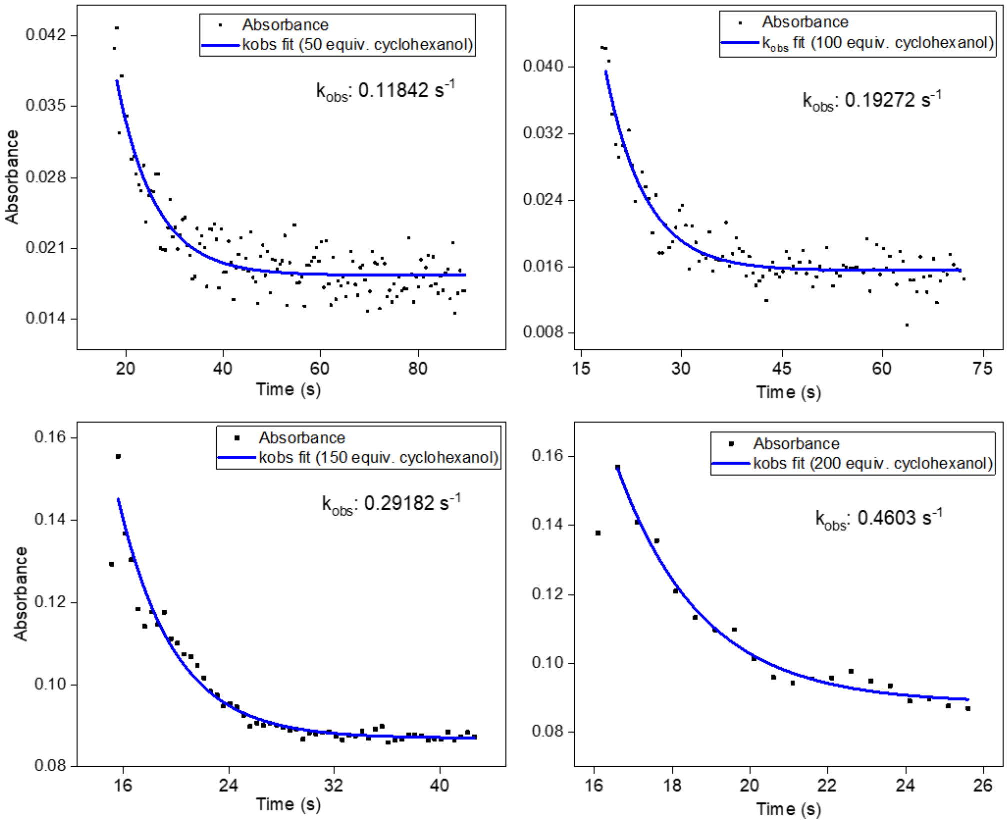

Figure S47. Kinetic traces for the reaction of complex **3** (0.045 mM) in 100% D_2O by *m*CPBA with cyclohexanol at various concentrations using the buffered solution to maintain pH.

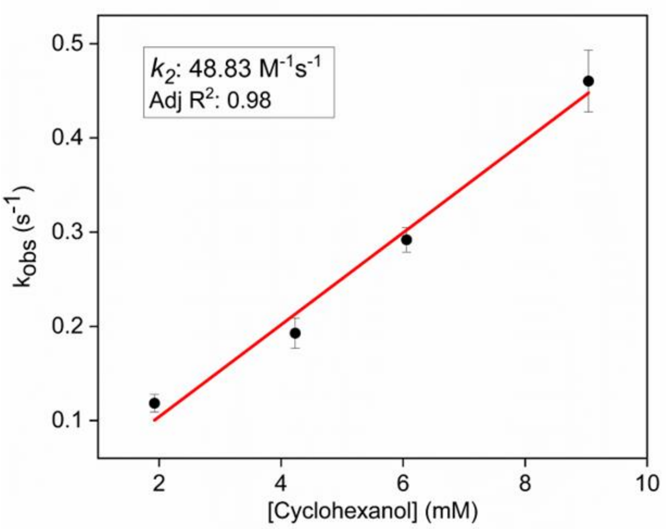

Figure S48. Determination of second order rate constant value of complex **3** for cyclohexanol oxidation in 100% D_2O with *m*CPBA.

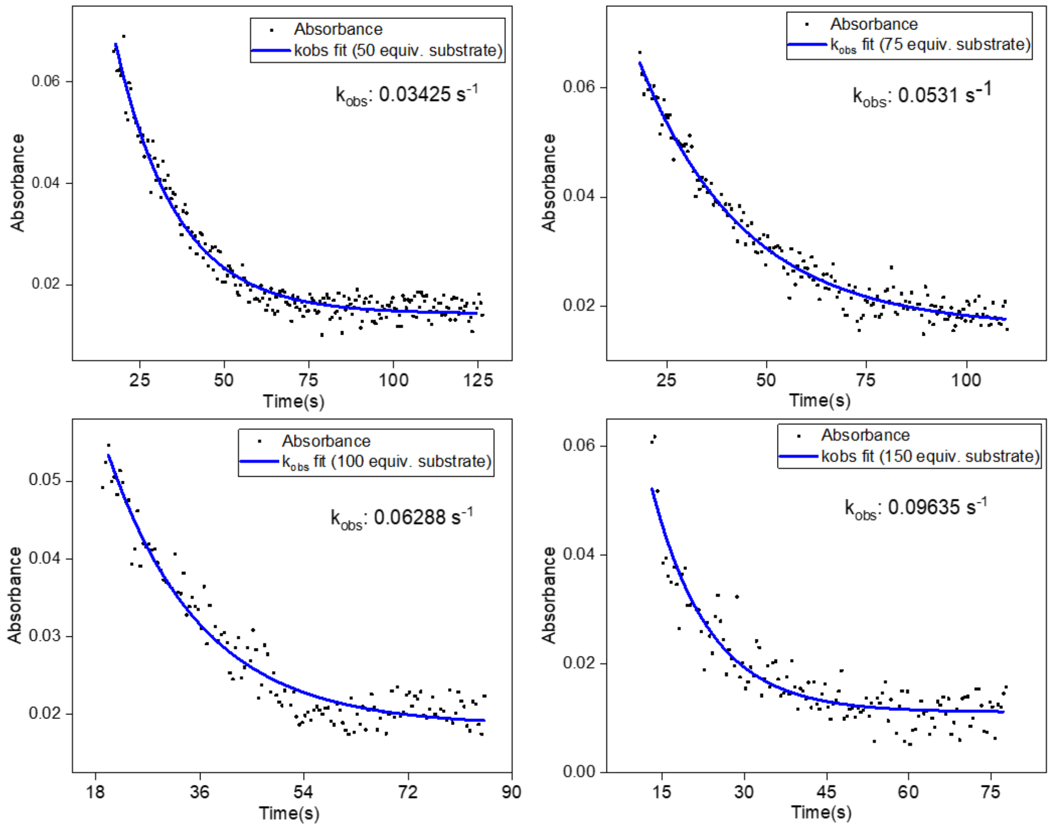

Figure S49. Kinetic traces for the reaction of complex **3** (0.045 mM) in 100% water by *m*CPBA with 6-methylheptan-2-yl isonicotinate at various concentrations. The kinetic traces were fitted to the equation, $[(A_t = A_\alpha - (A_\alpha - A_o)e(^{-k_{obs}t})]$ for obtaining k_{obs} values.

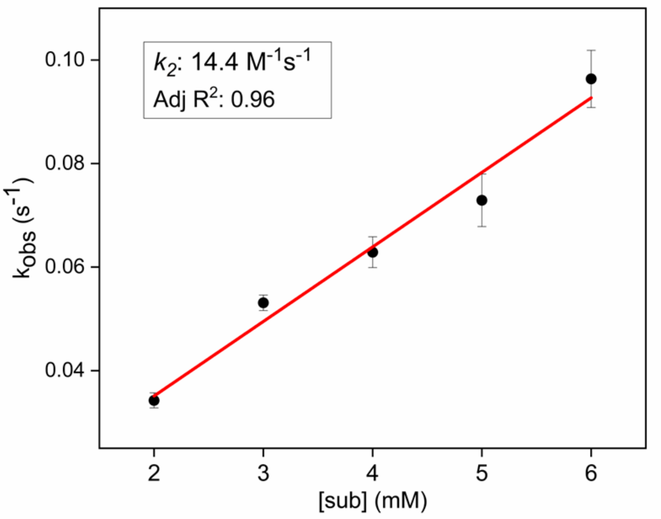

Figure S50. Determination of second order rate constant values of complex **3** for 6-methylheptan-2-yl isonicotinate oxidation in 100% H_2O with *m*CPBA.

Table S4. Product yields for oxidation of different hydrocarbons by complex **3** in 100% water by treatment with *m*CPBA at single turnover condition (the yield is greater than the theoretical yield of 25% ($4e^-$ oxidation) and 50% ($2e^-$ oxidation) since a slight excess of *m*CPBA is present in the solution):

C-H bond substrates	BDE_{C-H} (kcal mol^{-1})	Products	% GC yield
Cyclohexane	99.3	$C_6H_{11}OH$; $C_6H_{10}O$	32%
Cyclohexanol	92.4	$C_6H_{10}O$	75%
Toluene	90	PhCHO	35%
Ethyl benzene	87	$PhCH(OH)CH_3$; $PhC(O)CH_3$	62%
Cumene	84.5	$PhC(OH)(CH_3)_2$;	71%

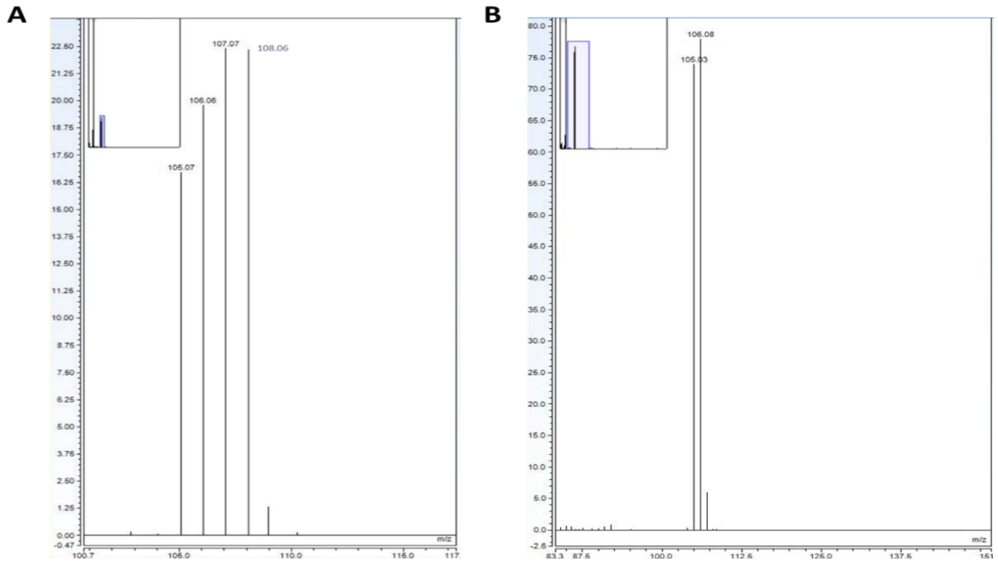

Figure S51. GC-MS data for the reaction of Complex **3** (2×10^{-4} M) with toluene (0.1M) in presence of (A) H_2O^{18} and (B) H_2O^{16}. 52% O^{18} incorporation was observed in benzaldehyde when reaction was carried out in presence of H_2O^{18}

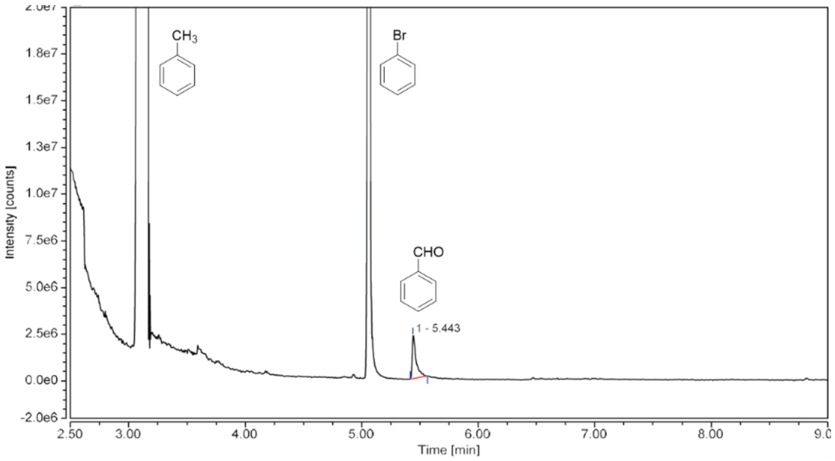

Figure S52. GC trace for the product formed upon reaction of **2** (2×10^{-4} M) with toluene (500 equiv).

3F. High valent oxo intermediate formation by H_2O_2 treatment

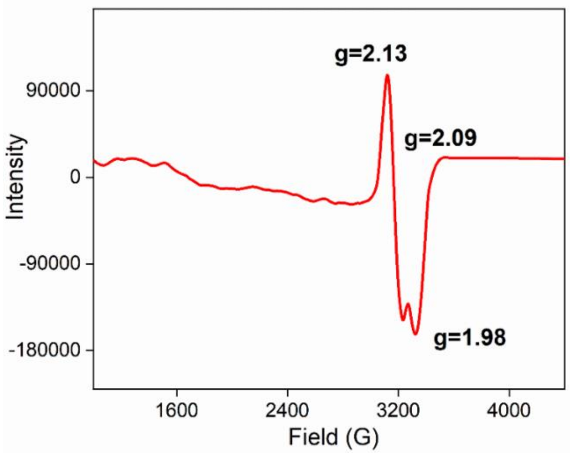

Figure S53. EPR of **3** formed upon treatment of 5 equiv hydrogen peroxide to **2** in 1:1 CH_3CN-aq. pH 8.5 phosphate buffer (8 mM) at 85 K; the g-values indicate the formation of S = ½ species.

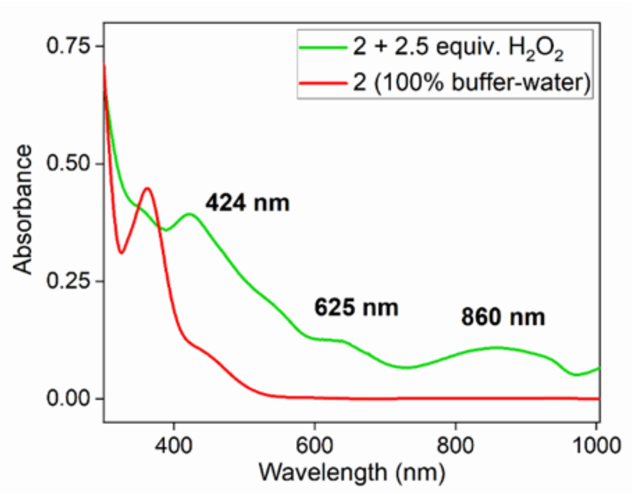

Figure S54. UV-Vis spectra of **2** (red) and the species (green) formed upon the addition of H_2O_2 to **2** in 100% aqueous water (phosphate buffer pH 8.5).

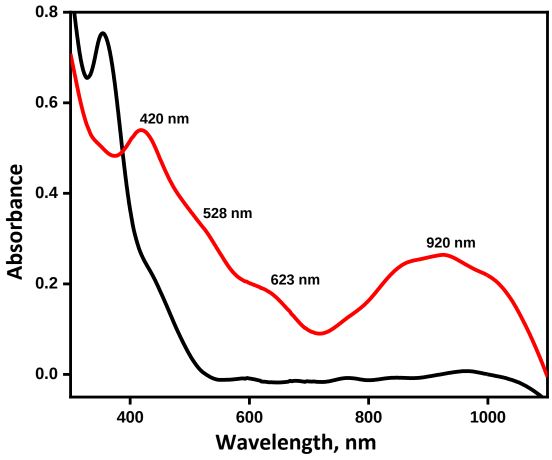

Figure S55. Formation of dimeric species (mixture of $Fe^{III}Fe^{IV}$ and $Fe^{IV}Fe^{IV}$, characteristic broad band at ~900 nm) (red) upon addition of H_2O_2 to **1A**(black) in both CH_3CN-water (1:1) mixture and 100% water medium.

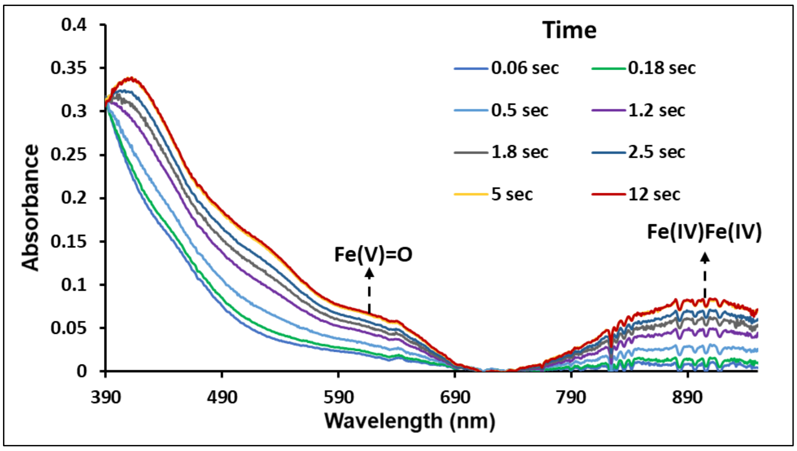

Figure S56. Stop flow formation kinetics of dimeric species (mixture of $Fe^{III}Fe^{IV}$ and $Fe^{IV}Fe^{IV}$, characteristic broad band at ~900 nm) upon addition of H_2O_2 to **1A** in CH_3CN-water (1:1) medium.

3G. Procedure for preparative hydroxylation reaction

To a 25 mL round-bottom flask containing a magnetic stir bar, catalyst **2** (2-16 µmol, 1-4 mol %), substrate (0.2-0.4 mmol scale) in 7 ml water (phosphate buffer, pH 8.5) were added. An aqueous solution of hydrogen peroxide (H_2O_2) (0.4-1.2 mmol, 2-3 equiv) was added *via a* syringe pump with continuous stirring over a period of 2-5 h at room temperature. The reaction was monitored by TLC and/or GC. After completion of the reaction, the excess H_2O_2 was quenched with a small amount of catalase. The resulting solution was saturated with NaCl and extracted with ethyl acetate (2-3 times). The organic part was dried with anhydrous Na_2SO_4, analyzed by GC-MS to estimate substrate conversion and then further purified by column chromatography to obtain the desired product (silica gel, 100-200 or 230-400 mesh). (NB: for more volatile substrates, evaporation was performed at reduced temperatures). The purity of the obtained product was determined by ^1H, ^{13}C NMR, HRMS and GC-MS. The regioselectivity and diastereoselectivity in the products are in accordance with the NMR data of the reported products as referenced in the characterization data.

3H. Epoxidation Reaction Procedure

To a 2 mL glass vial containing a magnetic stir bar, catalyst **2** (0.2 mM, 0.5-2 mol %), substrate (10-40 mM) in 1 ml water (phosphate buffer, pH 8.5) were added. An aqueous solution of hydrogen peroxide (H_2O_2) (1-2 equiv w.r.t substrate) was added *via a* syringe pump with continuous stirring over a period of 15-45 mins at room temperature. The reaction was monitored by GC-MS. After completion of the reaction, the excess H_2O_2 was quenched with a small amount of catalase. The resulting solution was saturated with NaCl and extracted with ethyl acetate (2-3 times). The organic part was dried with anhydrous Na_2SO_4. The product yield and substrate conversion were quantified by GC-MS.

3I. Substrate scopes for C-H oxidation and epoxidation in CH_3CN-water (1:1) solvent

Table S5. Substrate Scope for **3** catalyzed C-H bond oxidation in CH_3CN-water (1:1) solvent using H_2O_2 as the oxidant

**Sl. No.**	**Substrate**	**Product**	**% Isolated yield (GC conversion) [selectivity] in CH_3CN: water (1:1) solvent**
1	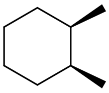	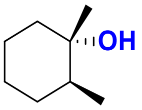 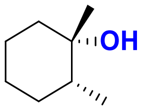	87(97%) [Cis: Trans 98:2]

2	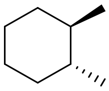	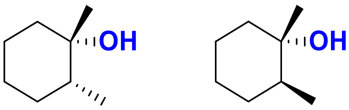	39(51%)<br>[Trans: Cis 97:3]
3	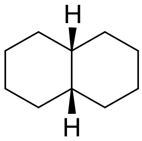	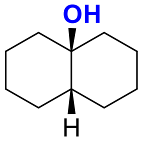	85(96%)<br>[100% Cis]
4	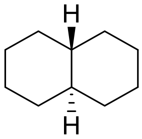	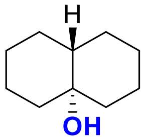	51(68%)<br>[Trans :Cis 98:2]
5	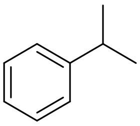	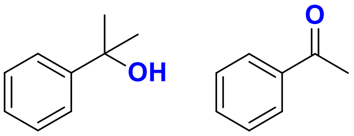	92(100%)<br>[A: K 80:20]
6	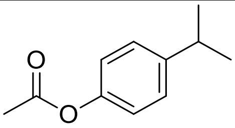	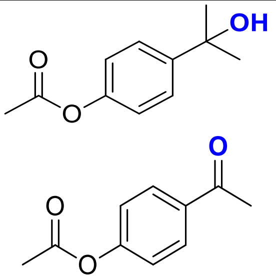	87(100%)<br>[A: K 95:5]
7	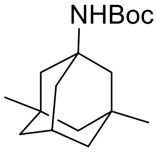	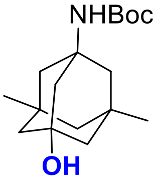	81(92%)<br>[100% 3°]
8	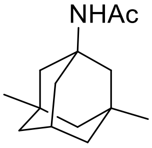	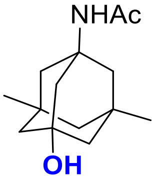	76(88%)<br>[100% 3°]
9	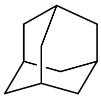	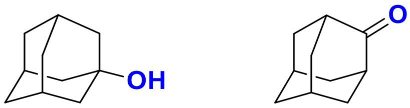	75(87%)<br>[100% 3°]
10	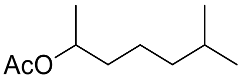	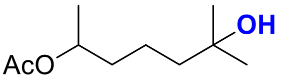	87(93%)<br>[100% A]
11	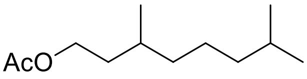	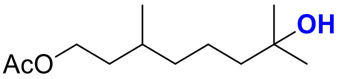	62(75%)<br>[100% A]
12	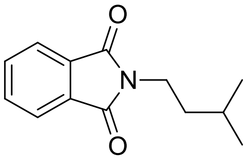	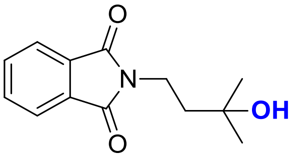	43(57%)<br>[100% 3°]

13	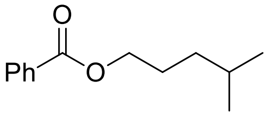	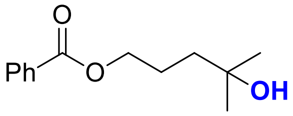	75(88%)<br>[3°:2° 90:10]
14	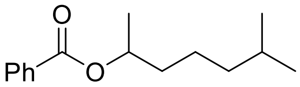	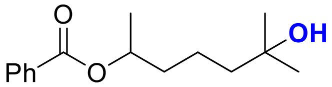	70(91%)<br>[3°:2° 84:16]
15	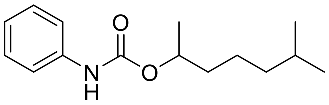	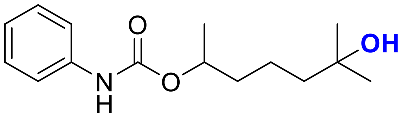	62(85%)<br>[3°:2° 85:15]
16	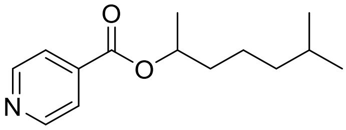	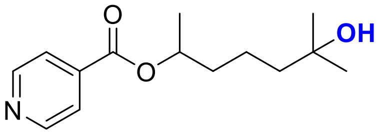	64(84%)<br>[3°:2° 84:16]
17	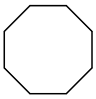	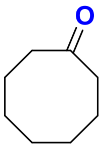 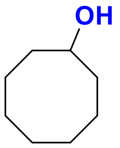	74(91%)<br>[K: A 90:10]
18	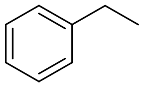	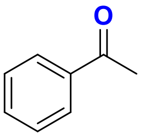	94(100%)<br>[100% K]
19	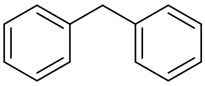	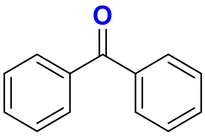	91(100%)<br>[100% K]
20	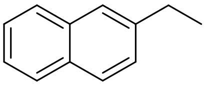	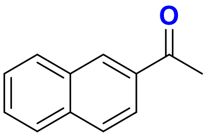	88(97%)<br>[100% K]
21	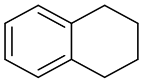	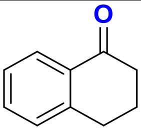	84(98%)<br>[100% K]
22	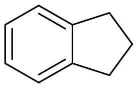	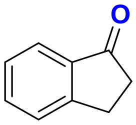	87(97%)<br>[100% K]
23	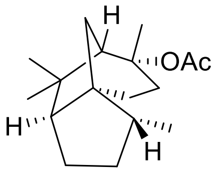	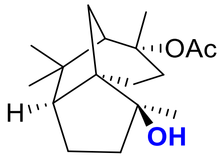	65(76%)<br>[100% A]
24	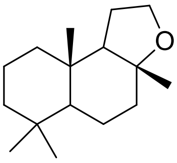	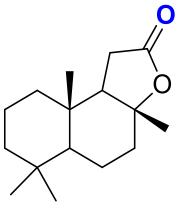	85(96%)<br>[100% K]

25	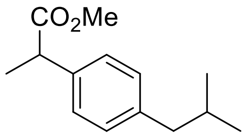	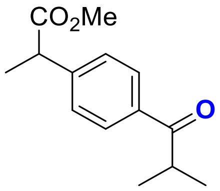 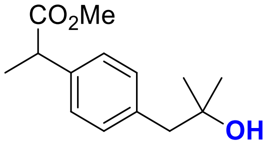	70(92%) [K: A 92:8]
26	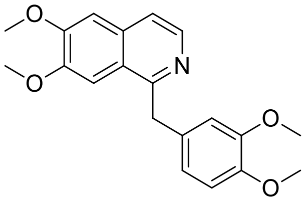	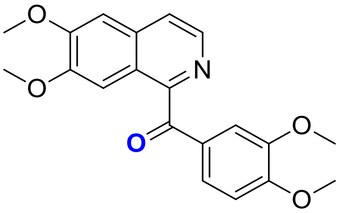	92(100%) [100% K]
27	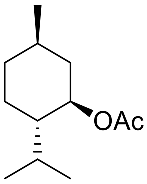	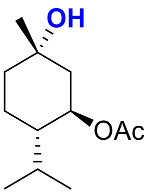 **27a** 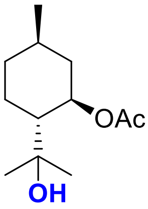 **27b**	69(82%) [25a :25b 80:20]

Table S6. Substrate Scope for **3** catalyzed epoxidation in acetonitrile-water (1:1) solvent using H_2O_2 as the oxidant

**Sl. No.**	**Substrate**	**Product**	**% GC yield (GC conversion) [selectivity] in CH_3CN-water (1:1) solvent**
1	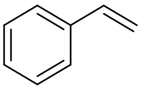	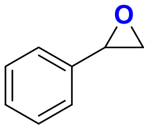	93(98%)
2	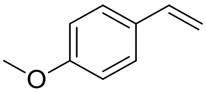	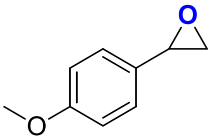	95(100%)
3	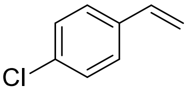	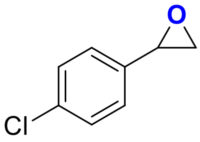	91(98%)
4	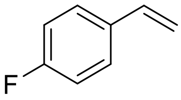	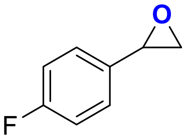	89(94%)

5	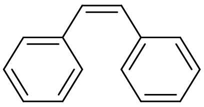	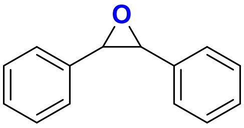	87(96%)<br>[Cis: Trans 90:10]
6	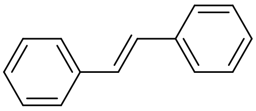	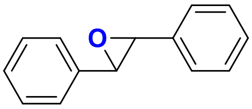	85(94%)<br>[100% Trans]
7	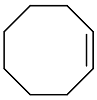	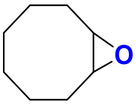	97(100%)

[a]0.2 mmol, [b]0.25 mmol, [c]0.27 mmol, [d]0.30 mmol, [e]0.4 mmol, [f]0.5 mmol scale; A and K denotes alcohol and ketone respectively. Loading: 2 mol% catalyst (1, 3, 5, 6, 10, 15, 16, 17, 18, 19, 20, 21, 22, 26); 3 mol% (2, 4, 7, 8, 9,11, 23, 24, 25); 4 mol% (12, 27, 13, 14); For hydroxylation, % isolated yield (GC conversion)[selectivity] and for epoxidation, % GC yield based on standard (bromobenzene) (conversion)[selectivity], for alkenes 1 mol% loading(28, 29, 30, 31, 34); 2 mol% (32, 33); here selectivity was determined by GC.

3J. Comparison Studies

Table S7. Comparison study of different C-H bond substrates in 100% water medium w.r.t. CH_3CN-water (1:1) medium using H_2O_2

Entry No.	Substrate	Product	% Isolated yield (GC conversion) [selectivity]	
			Water (100% water)	CH_3CN : water (1:1)
1	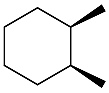	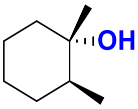 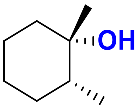	85 (100%) [100% cis]	87 (97%) [cis:trans 98:2]
2	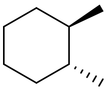	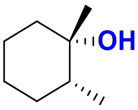 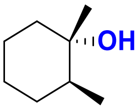	54 (68%) [100% trans]	39 (51%) [trans:cis 97:3]
5	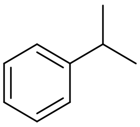	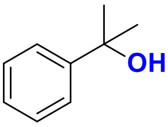 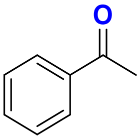	89 (96%) [100% A]	92 (100%) [A: K 80:20]
6	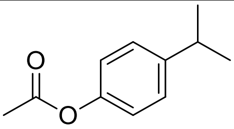	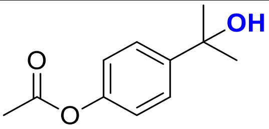 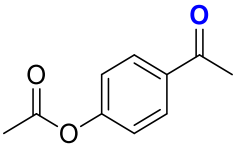	82 (95%) [100% A]	87 (100%) [A: K 95:5]

13	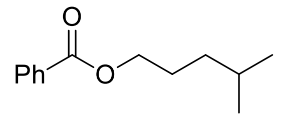	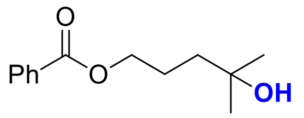	40 (50%) [100% 3°]	75 (88%) [3°:2° 90:10]
14	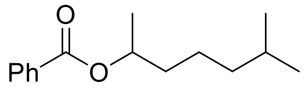	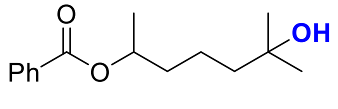	33 (49%) [100% 3°]	70 (91%) [3°:2° 84:16]

Table S8. Comparison between **3** catalyzed (H_2O_2, water and CH_3CN-water 1:1) and 1B′ catalyzed (*m*CPBA, CH_3CN-water 4:1) C-H bond oxidation.

**Entry No.**	**Product**	**% Isolated yield (GC conversion) [selectivity]**		
		**Water (100% buffer)**	**CH_3CN : water (1:1)**	**Previous Report[9]**
1	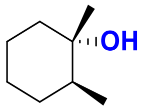 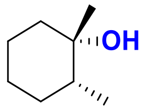	85 (100%) [100% cis]	87 (97%) [cis:trans 98:2]	85 (97%) [cis:trans 99:1]
2	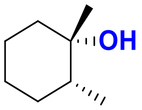 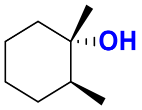	54 (68%) [100% trans]	39 (51%) [trans:cis 97:3]	60 (85%) [trans:cis 76:24]
3	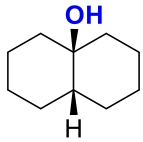	79 (92%) [100% cis]	85 (96%) [100% cis]	77 (90%) [cis:trans 99:1]
4	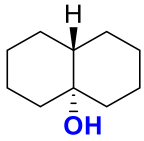	48 (65%) [100% trans]	51 (68%) [trans:cis 98:2]	27 (90%) [trans:cis 38:62]
7	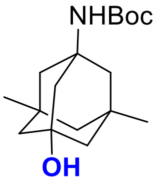	67 (82%) [100% 3°]	81 (92%) [100% 3°]	80 (93%) [3°:2° 94:6]
10	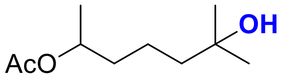	77 (95%) [100% 3°]	87 (93%) [100% 3°]	52 (76%) [3°:2° 75:25]
11	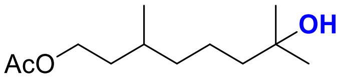	56 (75%) [100% 3°	62 (75%) [100% 3°]	51 (70%) [3°:2° 95:5]
15	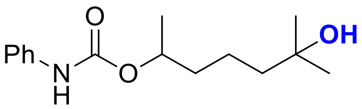	71 (92%) [3°:2° 86:14]	62 (85%) [3°:2° 85:15]	65 (85%) [3°:2° 86:14]

16	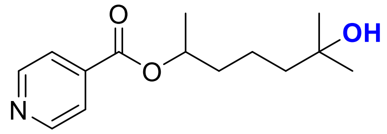	66 (84%) [3°:2° 85:15]	64 (84%) [3°:2° 84:16]	80 (93%) [3°:2° 89:11]
23	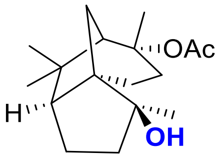	52 (68%) [100% A]	65 (76%) [100% A]	80 (89%) [100% A]
24	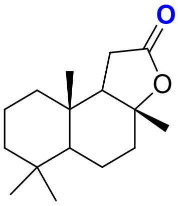	80 (93%) [100% K]	85 (96%) [100% K]	90 (99%) [100% K]
25	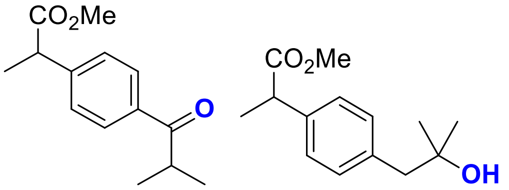	65 (86%) [K: A 92:8]	70 (92%) [K: A 92:8]	50 (82%)
26	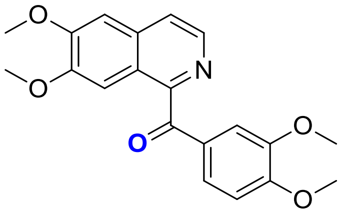	85 (95%) [100% K]	92 (100%) [100% K]	68 (76%) [100% K]
27	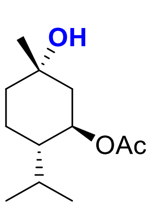 **27a** 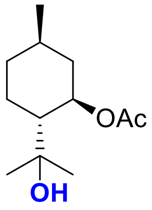 **27b**	62 (82%) [27a:27b 80:20] [100% A]	69 (82%) [27a:27b 80:20] [100% A]	55 (72%) [27a:27b 75:25] [3°:2° 85:15]

3K. Product Characterization Data

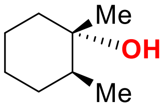

1. *(1S,2S)+(1R,2R)-1,2-dimethylcyclohexanol*[9] *(Table 2, entry 1):* (0.4 mmol scale) Purified by column chromatography using 5.7:1 hexane:EtOAc. Isolated yield: 85%, 44 mg. TLC R_f = 0.6 (4:1 hexane:EtOAc). ^{1}H NMR (400.13 MHz, $CDCl_3$) δ 1.73-1.63 (m, 4H), 1.53-1.47 (m, 1H), 1.41-1.29 (m, 2H), 1.28-1.25 (m, 2H), 1.10 (s, 3H), 0.93-0.91 (d, 3H, J = 6.85 Hz); ^{13}C NMR (100.61 MHz, $CDCl_3$) δ 73.09, 42.35, 41.39, 32.08, 25.32, 24.16, 20.88, 15.23 ppm; GC-MS (EI) m/z: M$^+$ peak: 128.20, base peak: 71.08, tR: 5.89 min.

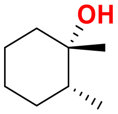

2. *(1S,2R)+(1R,2S)-1,2-dimethylcyclohexanol*[9] *(Table 2, entry 2):*(0.4 mmol scale) Purified by column chromatography using 19:1 hexane:EtOAc. Isolated yield: 44%, 23 mg. TLC R_f = 0.5 (7:3 hexane:EtOAc); ^{1}H NMR (400.13 MHz, $CDCl_3$, δ, ppm) δ 1.89-1.34 (m, 10H), 1.19 (s, 3H), 0.92-0.91 (d, 3H, J = 6.10 Hz); ^{13}C NMR (100.61 MHz, $CDCl_3$) δ 71.99, 41.97, 39.56, 31.14, 29.69, 24.91, 22.07, 14.71 ppm; GC-MS (EI) m/z: M^+ peak: 128.21, base peak: 71.02, tR: 5.59 min.

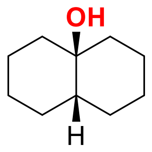

3. *cis-9-Decalinol*[9] *(Table 2, entry 3):* (0.4 mmol scale) Purified by column chromatography using 6:1 hexane:EtOAc. Isolated yield: 61%, 38 mg. TLC R_f = 0.7 (7:3 hexane:EtOAc). ^{1}H NMR (200.13 MHz, $CDCl_3$); δ 1.90−1.15 (m, 18H); ^{13}C NMR (50.32 MHz, $CDCl_3$) δ 71.78, 42.75, 29.67, 27.98 (br), 23.06 (br) ppm; GC-MS (EI) m/z: M^+ peak: 154.08, base peak: 111.04, tR: 8.97 min.

^{1}H NMR:

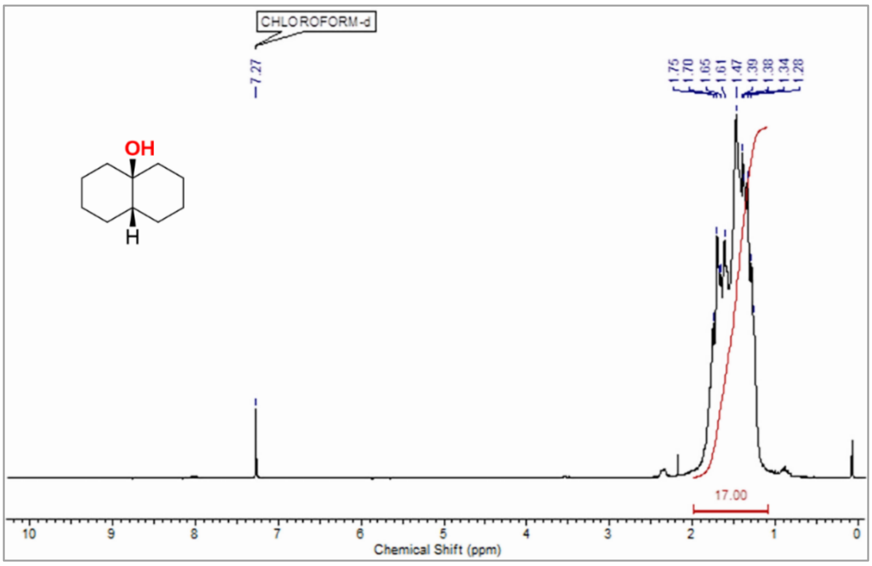

^{13}C NMR:

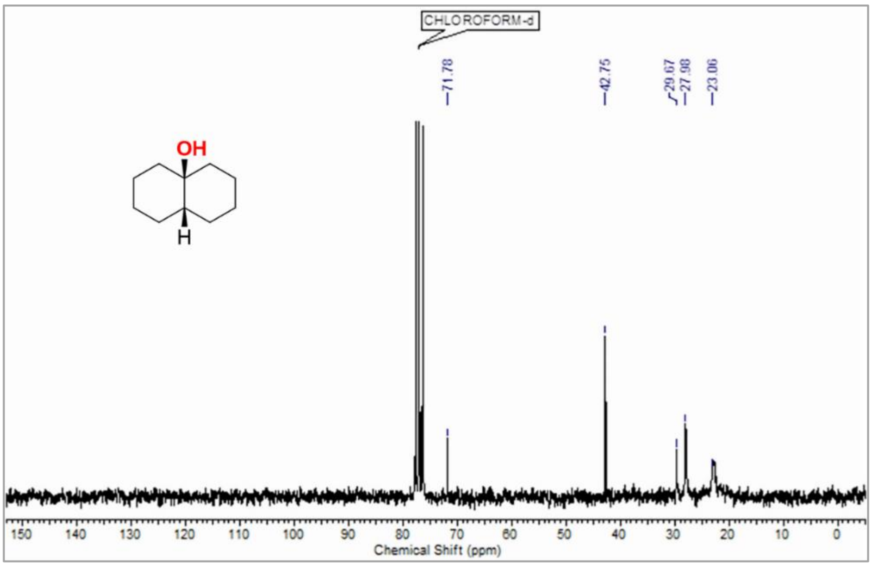

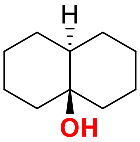

4. *trans-9-Decalinol*[9] *(Table 2, entry 4):* (0.6 mmol scale) Purified by column chromatography using 19:1 hexane:EtOAc. Isolated yield: 38%, 36 mg. TLC R_f = 0.6 (3:1 hexane:EtOAc); ^{1}H NMR (400.13 MHz, $CDCl_3$) δ 2.42-1.87 (m, 1H), 1.64-1.48 (m, 7H), 1.34-1.19 (m, 8H); ^{13}C NMR (50.32 MHz, $CDCl_3$) δ 71.33, 39.70, 28.63, 28.25, 21.63 ppm. GC-MS (EI) m/z: M^+ peak: 154.23, base peak: 111.05, tR: 8.62 min.

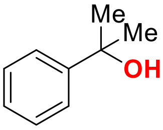

5. *2-Phenyl-2-propanol*[10] (Table 2, entry 5): (0.4 mmol scale) Purified by column chromatography using 19:1 hexane:EtOAc. Isolated yield: 89%, 48 mg. TLC R_f = 0.5 (4:1 hexane:EtOAc). ^{1}H NMR (500 MHz, $CDCl_3$) δ 7.51 (d, J = 8.2 Hz, 2H), 7.36 (t, J = 7.7 Hz, 2H), 7.26 (d, J = 14.8 Hz, 1H), 1.60 (s, 6H). ^{13}C NMR (126 MHz, $CDCl_3$) δ 149.23, 128.30, 126.77, 124.48, 72.61, 31.82. GC-MS (EI) m/z: M^+ peak: 135.71, base peak: 121.10, tR: 5.868 min.

^{1}H NMR:

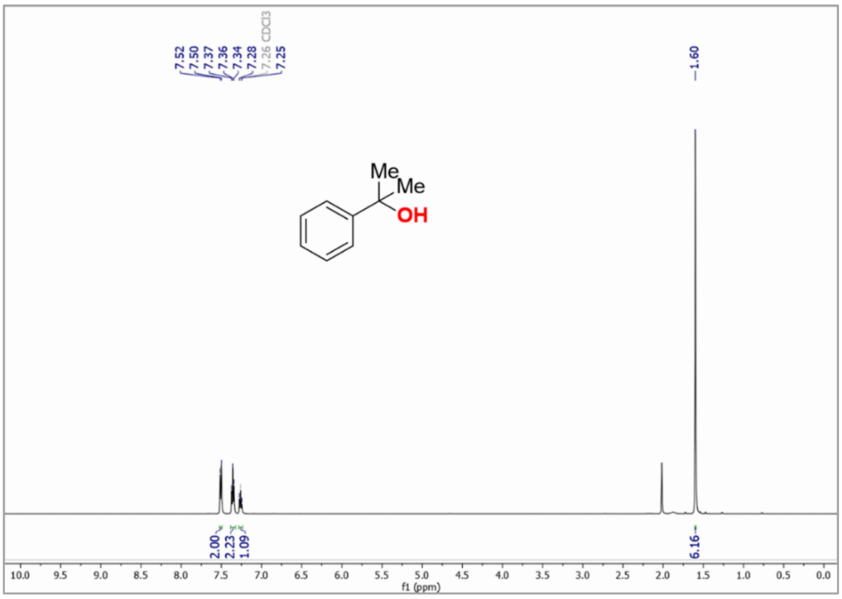

^{13}C NMR:

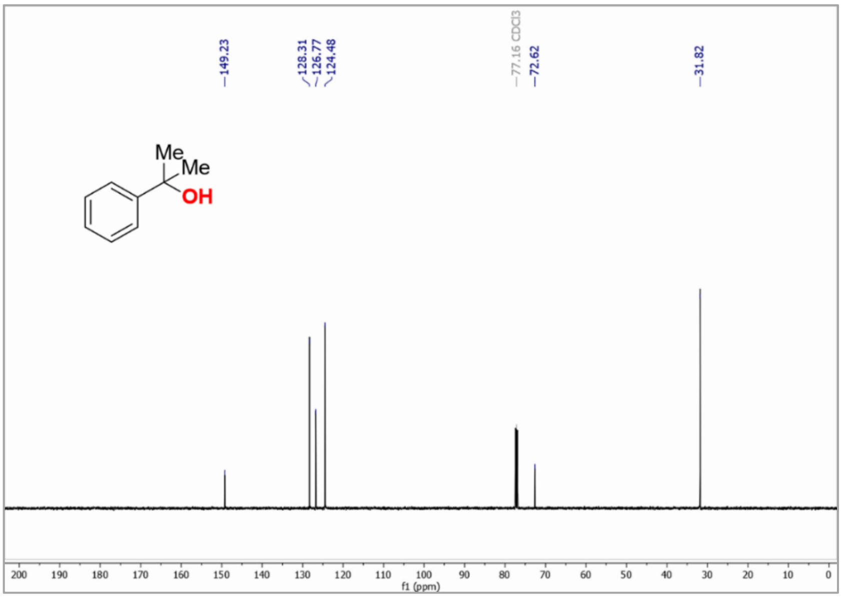

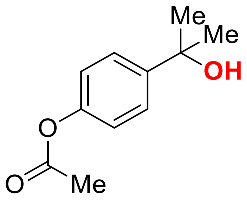

6. *4-(2-hydroxypropan-2-yl)phenyl acetate* (Table 2, entry 6): (0.4 mmol scale) Purified by column chromatography using 7:1 hexane:EtOAc. Isolated yield: 82%, 63 mg. TLC R_f = 0.3 (8:2 hexane:EtOAc). ^1H NMR (500 MHz, $CDCl_3$) δ 7.47 (d, J = 8.5 Hz, 2H), 7.02 (d, J = 8.5 Hz, 2H), 2.27 (s, 3H), 2.10 (s, 1H), 1.55 (s, 6H); ^{13}C NMR (126 MHz, $CDCl_3$) δ 169.73, 149.38, 146.83, 125.71, 121.18, 72.33, 31.82, 21.18. HRMS (ESI) m/z calc'd $C_{11}H_{14}O_3Na$ $[M+Na]^+$: 217.0943, found 217.0941.

^{1}H NMR:

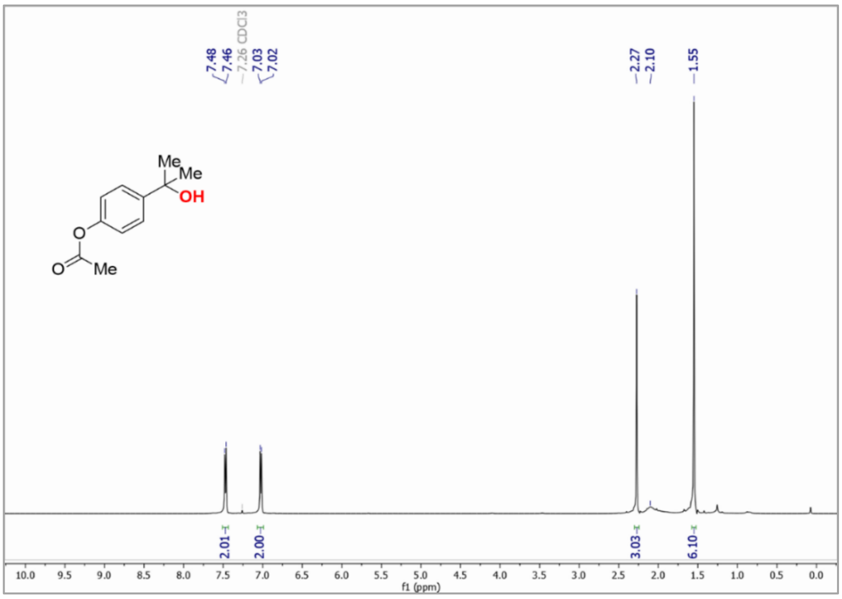

^{13}C NMR:

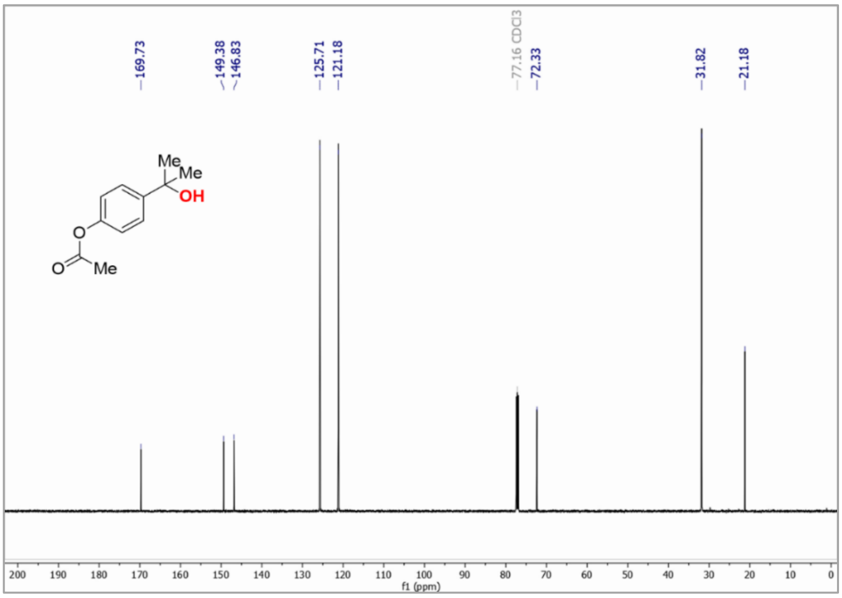

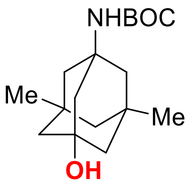

7. *tert-butyl((1r,3s,5R,7S)-3-hydroxy-5,7-dimethyladamantan-1-yl)carbamate*[9] *(Table 2, entry 7)*: (0.27 mmol scale) Purified by column chromatography using 1:1 hexane:EtOAc. Isolated yield: 67%, 53 mg. TLC R_f = 0.3 (7:3 hexane:EtOAc). ^{1}H NMR (400.13 MHz, $CDCl_3$) δ 4.47 (s, 1H), 1.79 (s, 2H), 1.59 (s, 4H), 1.473 (s, 9H), 1.38-1.36 (m, 4H), 1.10-1.09 (d, 2H, J = 4.48 Hz), 0.93 (s, 6H); ^{13}C NMR (100.61 MHz, $CDCl_3$) δ 153.87, 78.69, 70.08, 53.53, 50.11, 49.13,

47.70, 46.42, 33.82, 28.81, 28.11 ppm; HRMS (ESI) m/z calc'd $C_{17}H_{29}O_3NNa$ $[M+Na]^+$: 318.2040, found 318.2038.

^{1}H NMR:

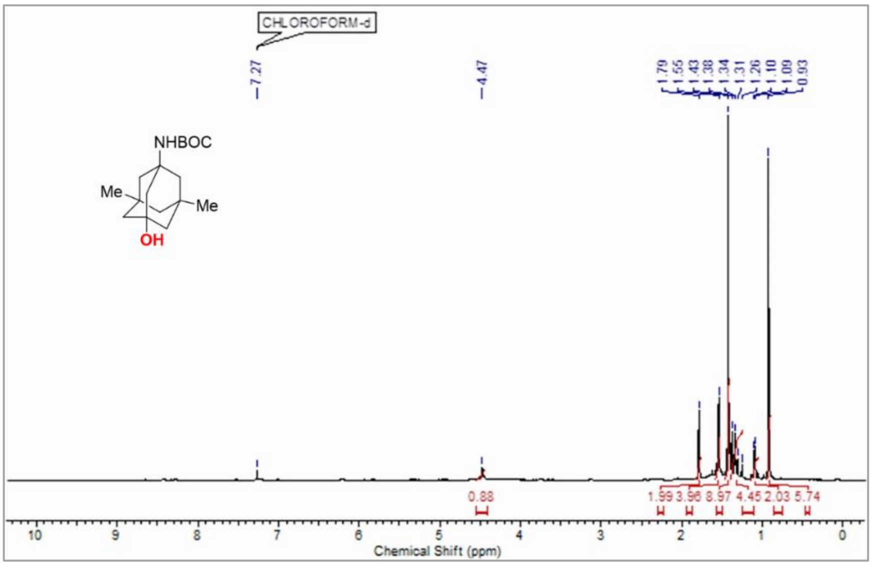

^{13}C NMR:

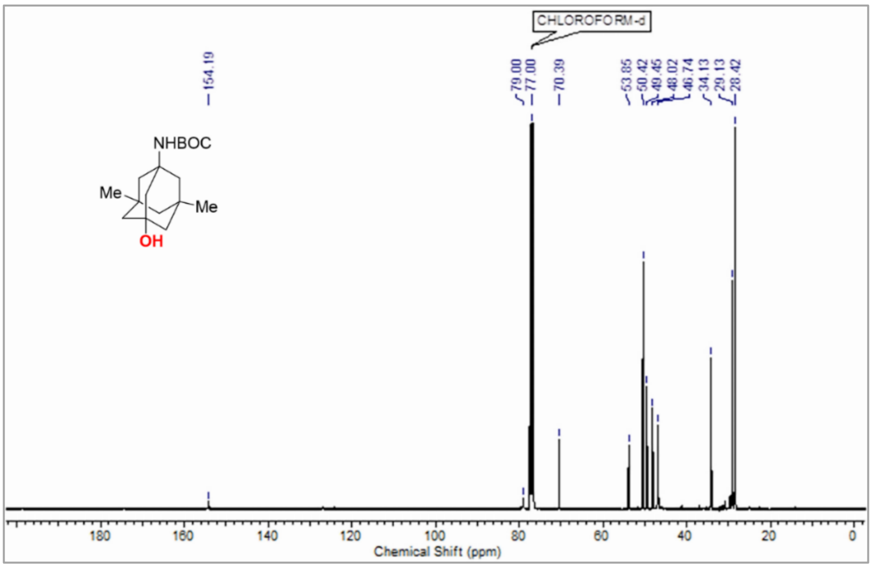

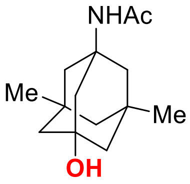

8.N-(3-Hydroxy-5,7-dimethyladamantan-1-yl)acetamide[11] *(Table 2, entry 8)*: Purified by column chromatography by using 1:4 hexane:EtOAc. Isolated yield (0.3 mmol scale): 52%, 37 mg. TLC R_f = 0.5 (EtOAc). ^{1}H NMR (500 MHz, $CDCl_3$) δ 5.25 (s, 1H), 1.91 (s, 3H), 1.87 (s, 2H), 1.60 (d, J = 12.4 Hz, 5H), 1.41 (d, J = 11.4 Hz, 2H), 1.34 (s, 2H), 1.11 (d, J = 11.1 Hz,

2H), 0.92 (s, 6H). ^{13}C NMR (126 MHz, $CDCl_3$) δ 169.67, 70.46, 55.30 (2C), 50.53, 49.61, 47.78, 46.63 (2C), 34.29, 29.84, 29.26, 24.72. HRMS (ESI) m/z calc'd $C_{14}H_{23}O_2NNa$ $[M+Na]^+$: 260.1729, found 260.1721.

^{1}H NMR:

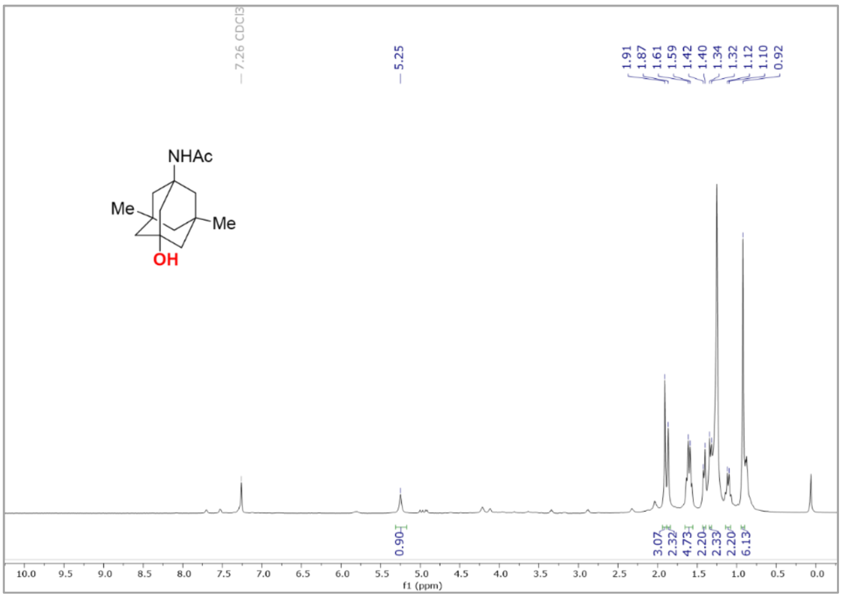

^{13}C NMR:

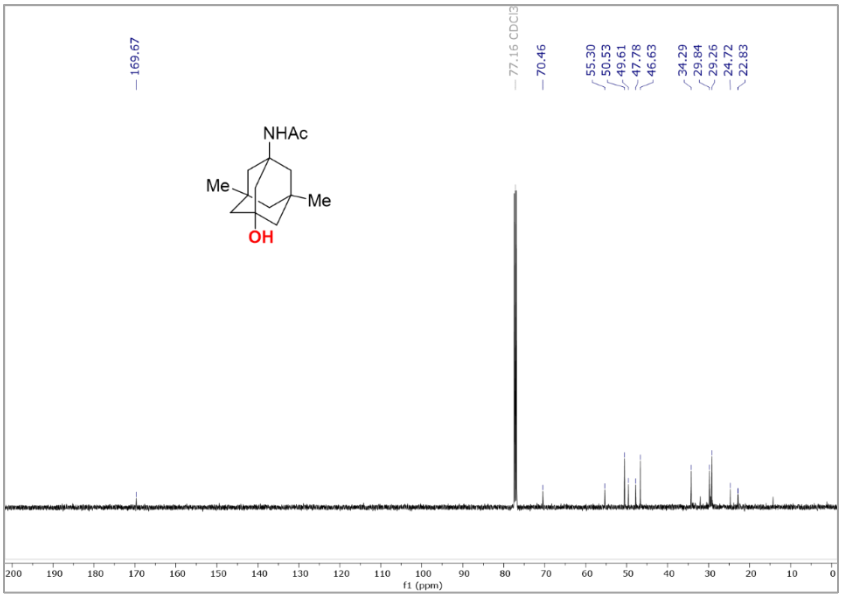

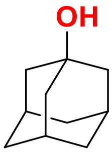

9. *Adamantan-1-ol*[12] (Table 2, entry 9): (0.4 mmol scale) Purified by column chromatography using 19:1 hexane:EtOAc. Isolated yield: 53%, 33 mg. TLC R_f = 0.5 (9:1 hexane:EtOAc). ^{1}H NMR (500 MHz, $CDCl_3$) δ 2.11 (br s, 3H), 1.69 (br d, J = 3.2 Hz, 6H), 1.59 (m, 6H), 1.56 (m,

1H). ^{13}C NMR (126 MHz, $CDCl_3$) δ 68.30, 45.45, 36.19, 30.69. GC-MS (EI) m/z: M^+ peak: 152.12, base peak: 95.07, tR: 7.75 min.

^{1}H NMR:

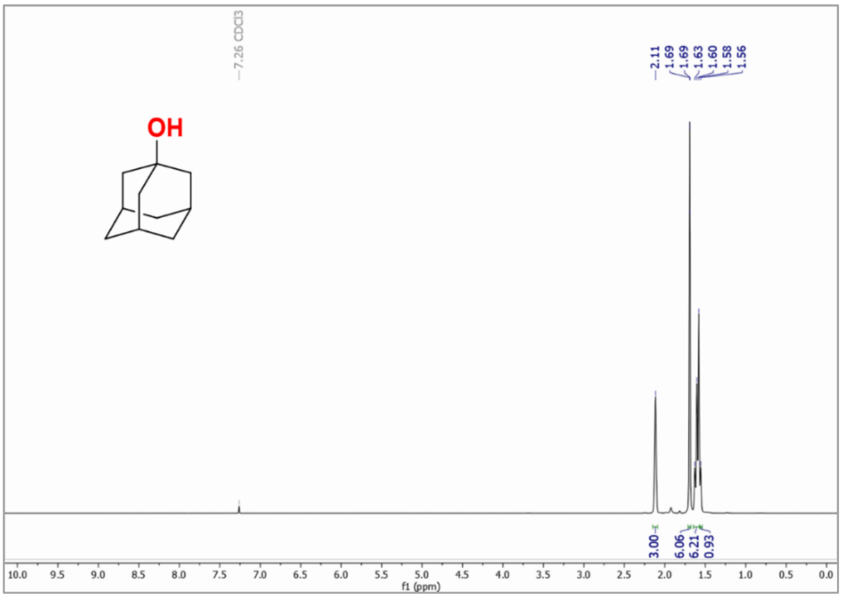

^{13}C NMR:

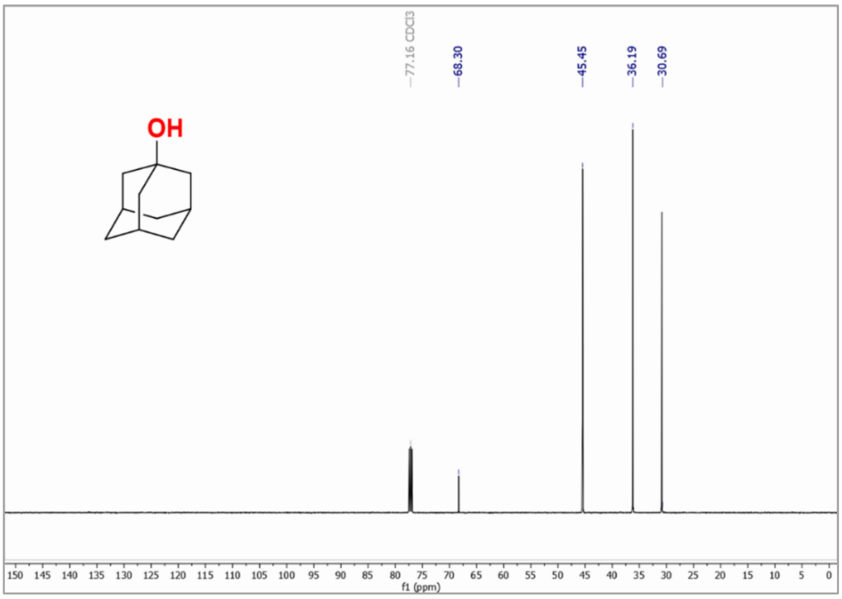

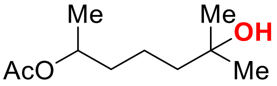

10. *6-hydroxy-6-methylheptan-2-yl acetate*[9] *(Table 2, entry 10):* Purified by column chromatography using 4:1 hexane:EtOAc. Isolated yield (0.4 mmol scale): 67%, 55 mg. TLC R_f = 0.6 (7:3 hexane:EtOAc). ^{1}H NMR (500 MHz, $CDCl_3$) δ 4.90 (q, J = 6.3 Hz, 1H), 2.01 (s, 3H), 1.59 (dq, J = 8.9, 4.1 Hz, 1H), 1.50 – 1.36 (m, 5H), 1.23 (d, J = 9.2 Hz, 3H), 1.19 (s, 6H);

^{13}C NMR (126 MHz, $CDCl_3$) δ 171.03, 71.03, 70.97, 43.62, 36.45, 29.81, 29.28, 21.50, 20.26, 20.10 ppm; HRMS (ESI) m/z calc'd $C_{10}H_{20}O_3Na$ $[M+Na]^+$: 211.1305, found 211.1306.

^1H NMR:

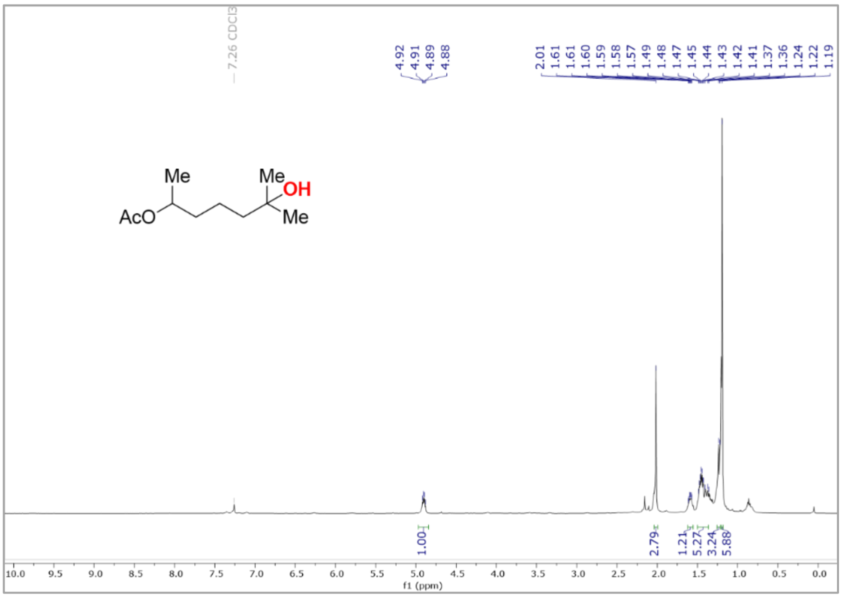

^{13}C NMR:

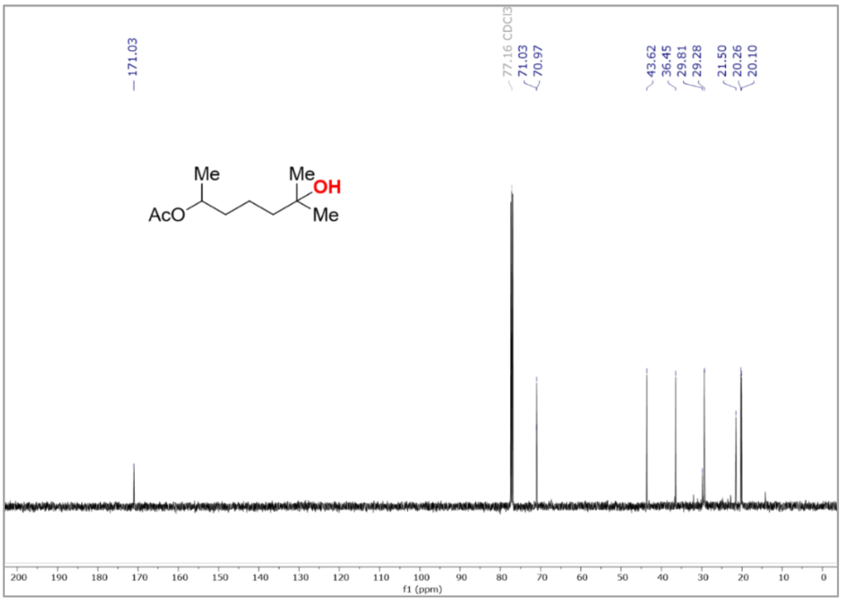

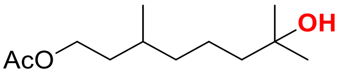

11. *7-hydroxy-3,7-dimethyloctyl acetate*[9] (Table 2, entry 11): (0.4 mmol scale) Purified by column chromatography using 4:1 hexane:EtOAc. Isolated yield: 51%, 44 mg. TLC R_f = 0.5 (3:1 hexane:EtOAc). ^1H NMR (500 MHz, $CDCl_3$) δ 4.15 – 4.04 (m, 2H), 2.04 (s, 3H), 1.70 – 1.62 (m, 2H), 1.57 – 1.52 (m, 2H), 1.47 – 1.38 (m, 4H), 1.35 – 1.29 (m, 2H), 1.21 (s, 6H), 0.91 (d, J = 6.6 Hz, 3H). ^{13}C NMR (126 MHz, $CDCl_3$) δ 171.41, 71.18, 63.17, 44.25, 37.52, 35.63,

29.99, 29.46, 29.40, 21.75, 21.19, 19.61. HRMS (ESI) m/z calc'd $C_{12}H_{24}O_3Na$ $[M+Na]^+$: 239.1618, found 239.1619.

^{1}H NMR:

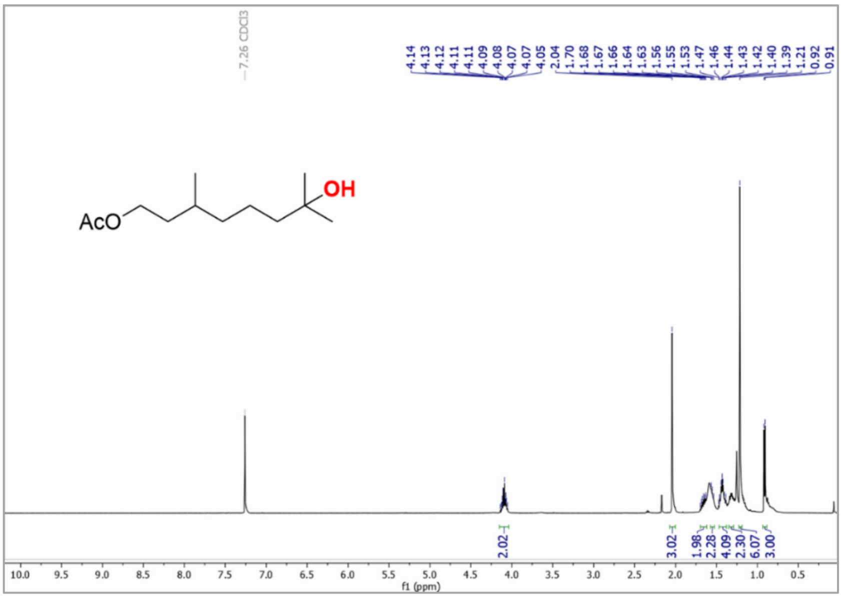

^{13}C NMR:

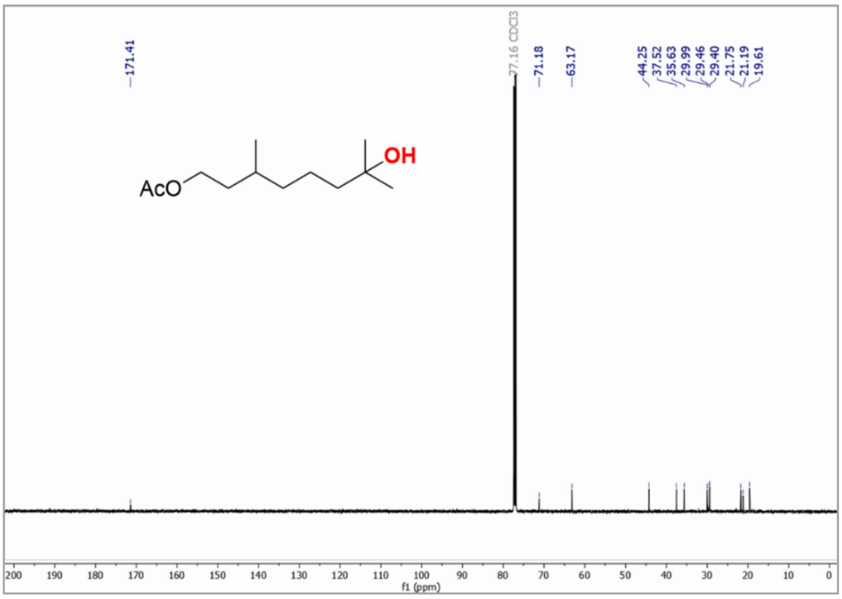

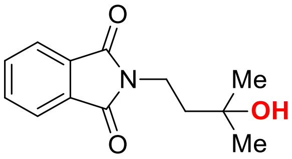

12. *2-(3-hydroxy-3-methylbutyl)isoindoline-1,3-dione*[13] *(Table 2, entry 12):* (0.4 mmol scale) The product was purified over silica gel column chromatography using 1:9 EtOAc-hexane as the eluent, gave the product as a colourless oil. Isolated yield 56 mg, 61%; TLC R_f = 0.4 (1:4

EtOAc/hexane). ^{1}H NMR (500 MHz, $CDCl_3$) δ 7.85 – 7.81 (m, 2H), 7.72 – 7.67 (m, 2H), 3.84 (t, J = 7.6 Hz, 2H), 2.76 (s, 1H), 1.85 (t, J = 7.6 Hz, 2H), 1.30 (s, 6H). ^{13}C NMR (126 MHz, $CDCl_3$) δ 168.63, 134.44, 134.03, 123.72, 123.33, 70.07, 41.56, 34.13, 29.54. HRMS (ESI) m/z calc'd $C_{13}H_{15}NO_3Na$ $[M+Na]^+$: 256.1052, found 256.1047.

^{1}H NMR:

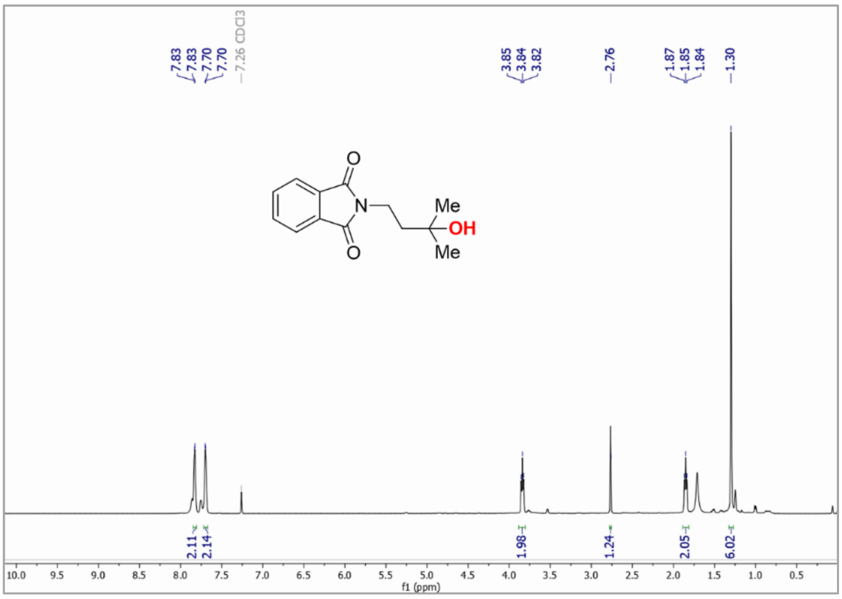

^{13}C NMR:

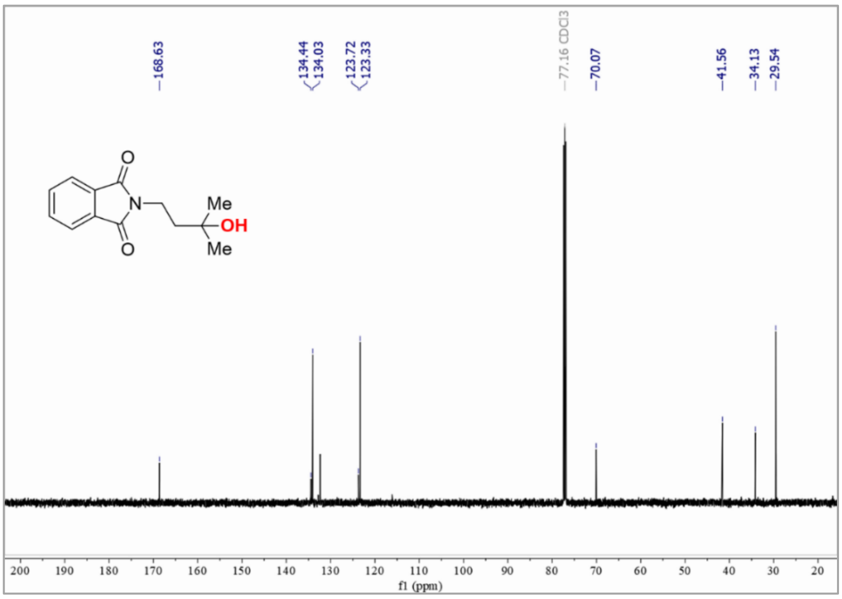

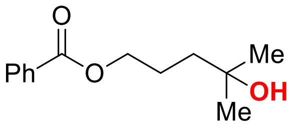

13. *4-hydroxy-4-methylpentyl benzoate*[14] *(Table 2, entry 13)*: (0.4 mmol scale) The product was purified by chromatography on silica gel (1:3 EtOAc/hexanes); colourless oil was obtained;

isolated yield 39 mg, 40%; TLC R_f = 0.5 (3:7 EtOAc/hexanes) ^{1}H NMR (500 MHz, $CDCl_3$) δ 8.04 (d, *J* = 7.6 Hz, 2H), 7.55 (t, *J* = 6.9 Hz, 1H), 7.44 (t, *J* = 7.4 Hz, 2H), 4.35 (t, *J* = 6.4 Hz, 2H), 1.94 – 1.80 (m, 2H), 1.72 – 1.53 (m, 2H), 1.26 (s, 6H). ^{13}C NMR (126 MHz, $CDCl_3$) δ 166.81, 133.02, 130.52, 129.68, 128.49, 70.87, 65.50, 40.13, 29.83, 29.44, 24.03. HRMS (ESI) m/z calc'd $C_{13}H_{18}O_3Na$ $[M+Na]^+$: 245.1256, found 245.1301

^{1}H NMR:

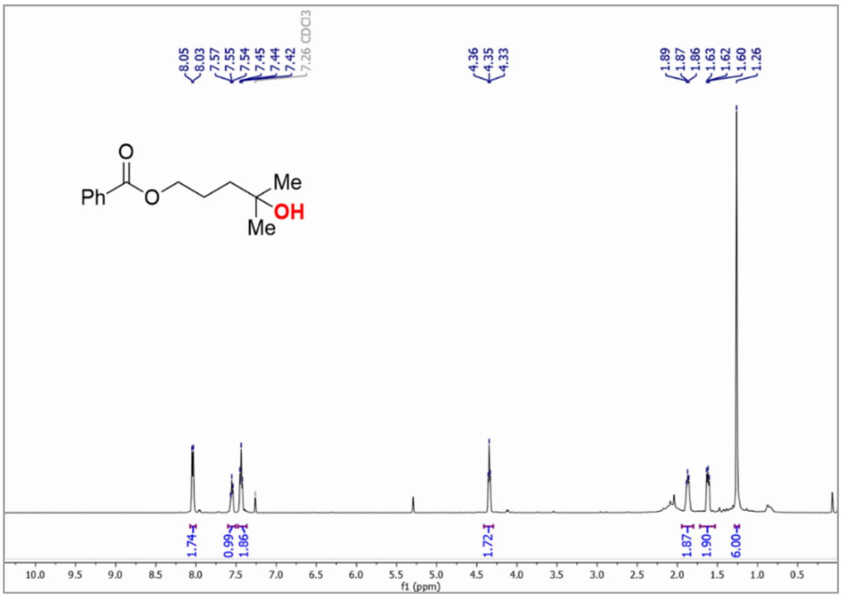

^{13}C NMR:

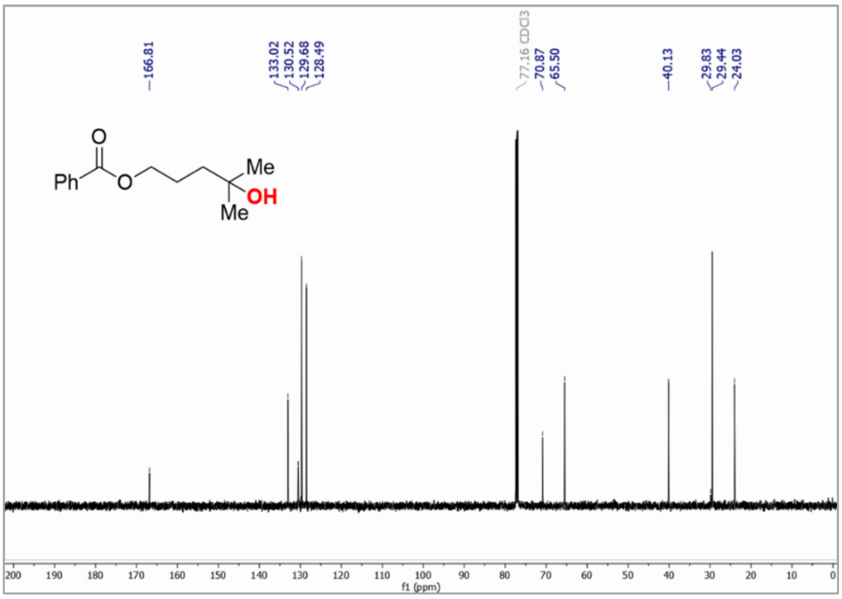

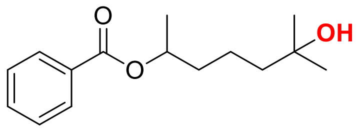

14. *6-hydroxy-6-methylheptan-2-yl benzoate*[9] *(Table 2, entry 14)*: (0.4 mmol scale) Purified by column chromatography using 3:1 hexane:EtOAc. Isolated yield: 23%, 24 mg. TLC R_f = 0.5 (7:3 hexane:EtOAc). ^{1}H NMR (400.13 MHz, $CDCl_3$) δ 8.06-8.04 (d, 2H, J = 7.82), 7.58-7.54 (t, 1H, J = 14.67 Hz), 7.46-7.43 (2H, t, J = 15.16 Hz), 5.22-5.17 (m, 1H), 1.79-1.75 (m, 1H), 1.66-1.62 (m, 1H), 1.54-1.50 (m, 3H), 1.46-1.42 (m, 2H), 1.37-1.35 (d, 3H, J = 6.36 Hz), 1.21 (s, 6H); ^{13}C NMR (100.61 MHz, $CDCl_3$) δ 166.56, 133.04, 131.12, 129.81, 128.60, 71.77, 71.17, 43.86, 36.80, 29.99, 29.59, 29.51, 20.51, 20.40 ppm; HRMS (ESI) m/z calc'd for $C_{15}H_{22}O_3Na$ $[M+Na]^+$: 273.1461, found 273.1459.

^{1}H NMR:

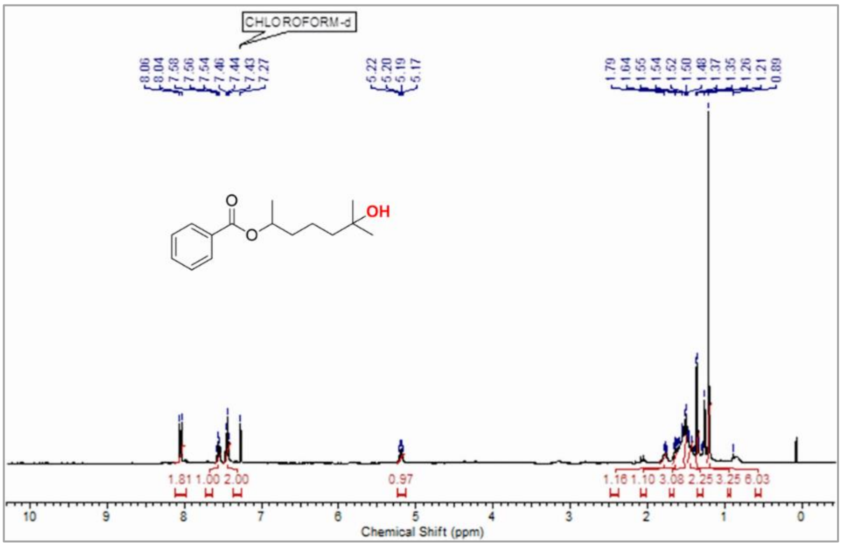

^{13}C NMR:

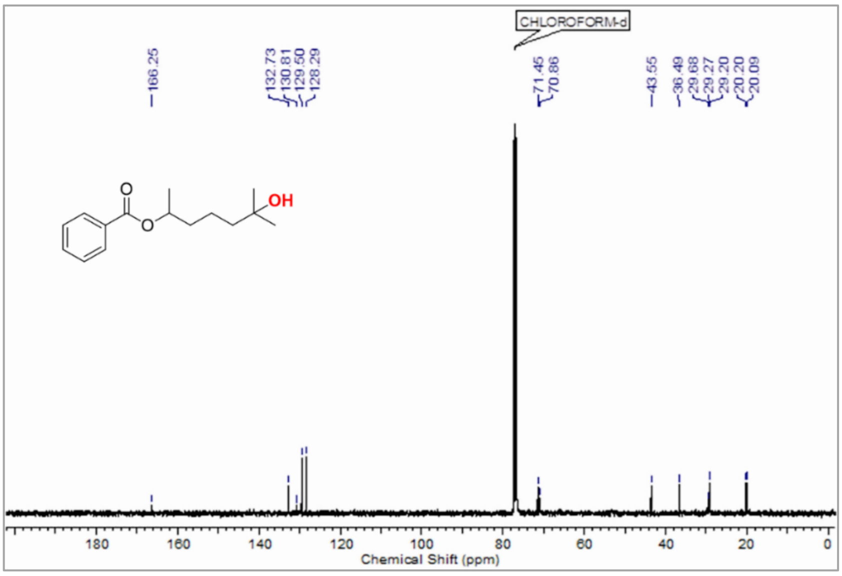

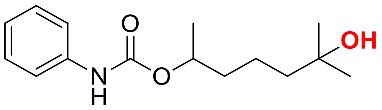

15. *6-hydroxy-6-methylheptan-2-yl phenylcarbamate*[9] *(Table 2, entry 15)*: (0.2 mmol scale) Purified by column chromatography using 7:3 hexane:EtOAc. Isolated yield: 71%, 38 mg. TLC R_f = 0.4 (7:3 hexane:EtOAc). ^{1}H NMR (400.13 MHz, $CDCl_3$) δ 7.40-7.38 (d, 2H, *J* = 8.31 Hz), 7.33-7.27 (m, 2H), 7.08-7.04 (t, 1H, *J* = 14.64 Hz), 6.59 (s, 1H), 4.96-4.92 (m, 1H), 1.62 (bs, 2H), 1.51-1.45 (m, 4H), 1.30-1.29 (d, 2H, *J* = 6.36 Hz), 1.22 (s, 6H); ^{13}C NMR (100.61 MHz, $CDCl_3$) δ 153.68, 138.36, 129.34, 123.58, 118.86, 72.19, 71.18, 43.86, 36.88, 29.64, 29.54, 20.57, 20.45 ppm; HRMS (ESI) m/z calc'd $C_{15}H_{23}O_3NNa$ $[M+Na]^+$: 288.1570, found 288.1568.

^{1}H NMR:

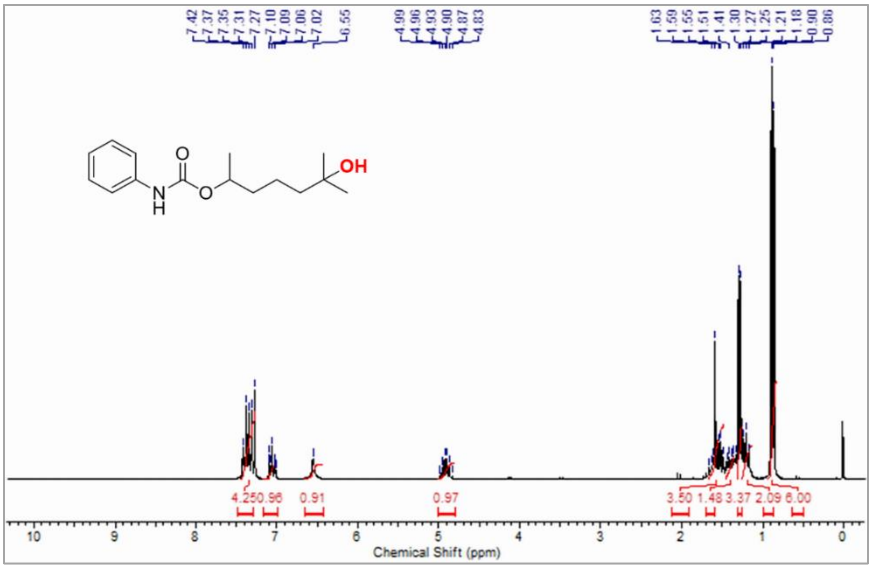

^{13}C NMR:

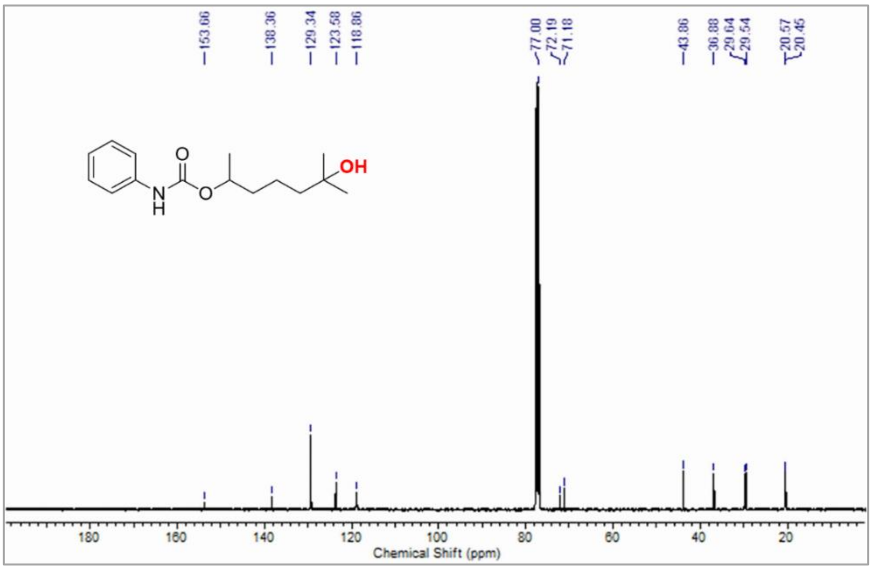

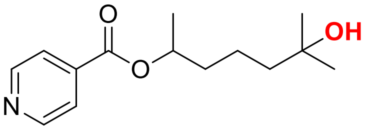

16. *6-hydroxy-6-methylheptan-2-yl isonicotinate*[9] *(Table 2, entry 16)*: (0.4 mmol scale) Purified by column chromatography using 1:1 hexane:EtOAc. Isolated yield: 66%, 66 mg. TLC R_f = 0.3 (1:1 hexane:EtOAc). ^{1}H NMR (400.13 MHz, $CDCl_3$) δ 8.78-8.76 (d, 2H, J = 5.49 Hz), 7.85-7.84 (d, 2H, J = 6.10 Hz), 5.25-5.17 (sxt, J = 6.2x(5), 1H), 1.79-1.74 (m, 1H), 1.67-1.62 (m, 1H), 1.51-1.49 (m, 3H), 1.38-1.36 (d, 3H J = 6.10 Hz), 1.26 (bs, 2H), 1.21 (s, 6H); ^{13}C NMR (100.61 MHz, $CDCl_3$) δ 165.02, 150.81, 138.27, 123.14, 73.03, 71.05, 43.75, 36.64, 29.98, 29.56, 20.44, 20.26 ppm; HRMS (ESI) m/z calc'd $C_{14}H_{22}O_3N$ $[M+Na]^+$: 252.1594, found 252.1595

^{1}H NMR:

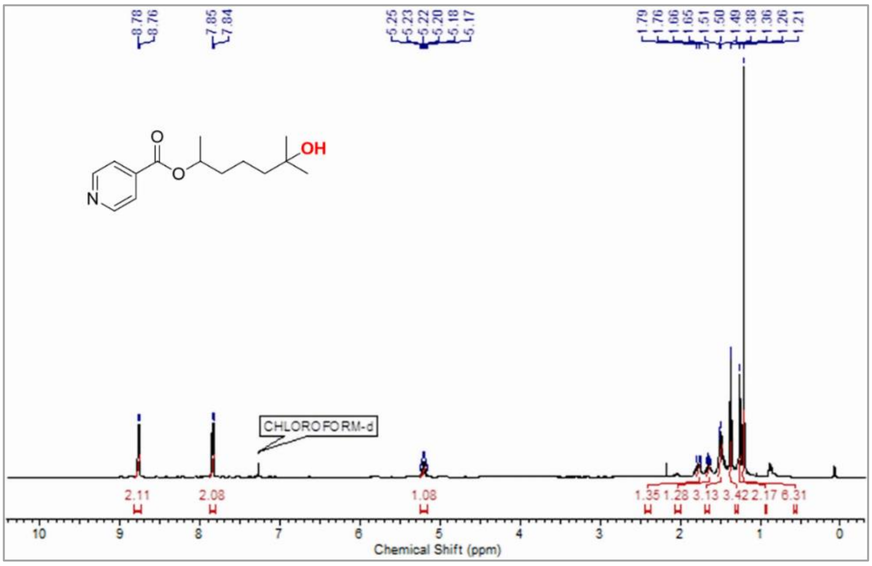

^{13}C NMR:

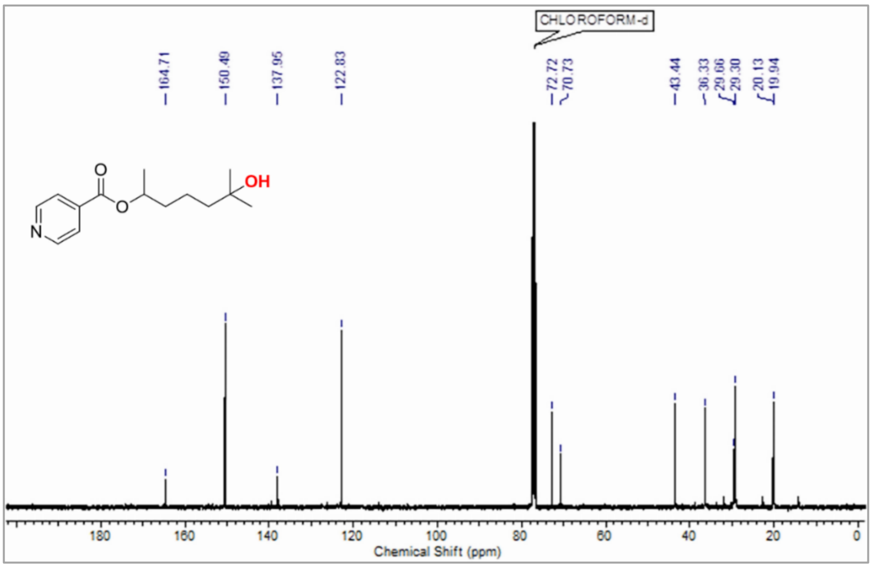

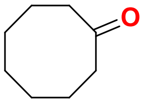

17. *Cyclooctanone*[15] (Table 2, entry 17): (0.4 mmol scale) Purified by column chromatography using 19:1 hexane:EtOAc. Isolated yield: 85%, 44 mg. TLC R_f = 0.6 (4:1 hexane:EtOAc). ^{1}H NMR (500 MHz, $CDCl_3$) δ 2.41 – 2.37 (m, 4H), 1.89 – 1.83 (m, 4H), 1.56 – 1.50 (m, 4H), 1.39 – 1.32 (m, 2H). ^{13}C NMR (126 MHz, $CDCl_3$) δ 218.48, 42.06, 27.28, 25.78, 24.83. GC-MS (EI) m/z: M^+ peak: 126.01, base peak: 97.99, tR: 7.36 min.

^{1}H NMR:

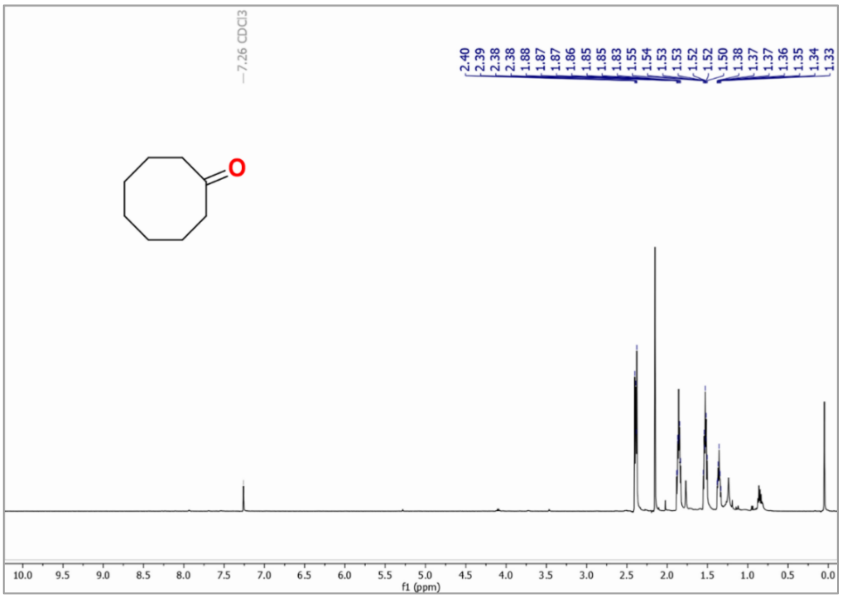

^{13}C NMR:

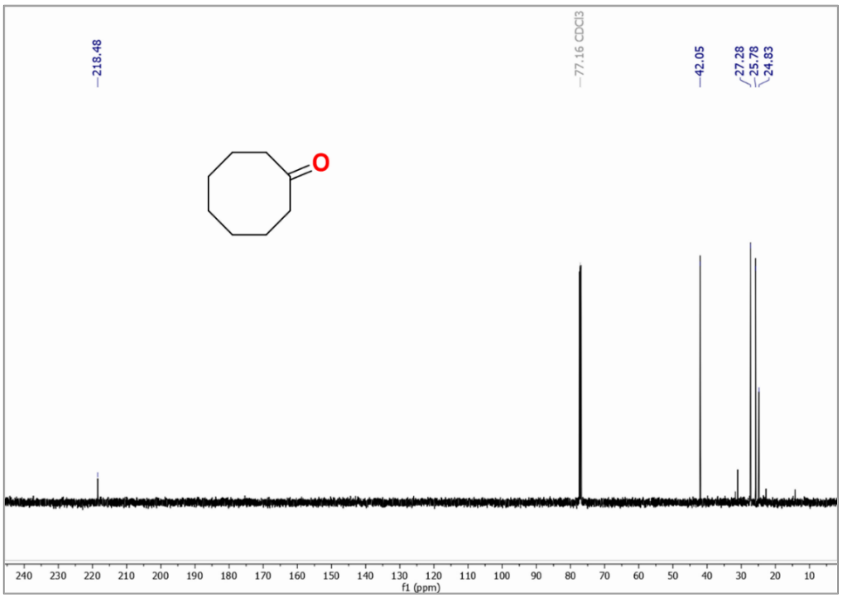

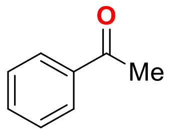

18. *Acetophenone*[16] (Table 2, entry 18): (0.4 mmol scale) Purified by column chromatography using 19:1 hexane:EtOAc. Isolated yield: 93%, 45 mg. TLC R_f = 0.6 (9:1 hexane: EtOAc). ^{1}H NMR (500 MHz, $CDCl_3$) δ 7.91 (d, *J* = 7.2 Hz, 2H), 7.51 (t, *J* = 7.4 Hz, 1H), 7.41 (t, *J* = 7.7 Hz, 2H), 2.55 (s, 3H). ^{13}C NMR (126 MHz, $CDCl_3$) δ 198.09, 137.07, 133.06, 128.52, 128.24, 26.51. GC-MS (EI) m/z: M^+ peak: 120.09, base peak: 105.07, tR: 5.69 min.

^{1}H NMR:

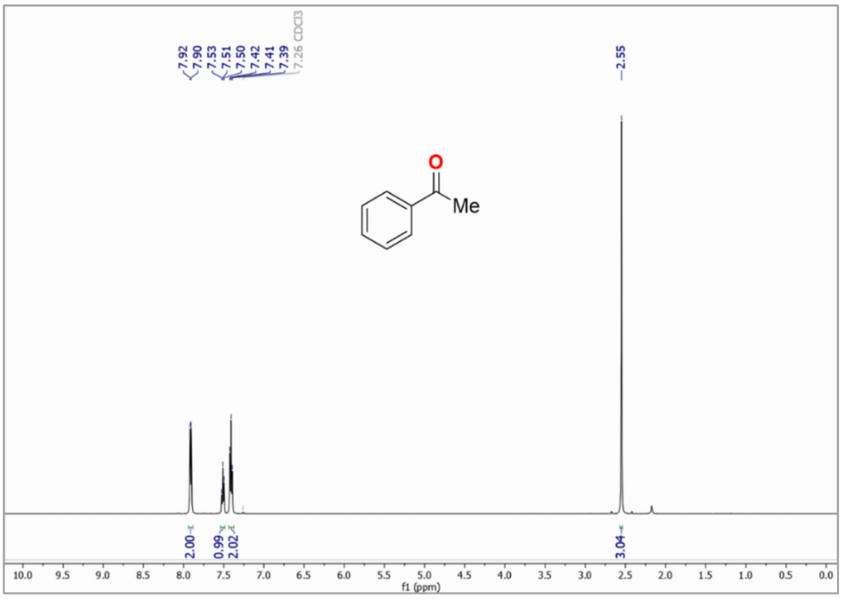

^{13}C NMR:

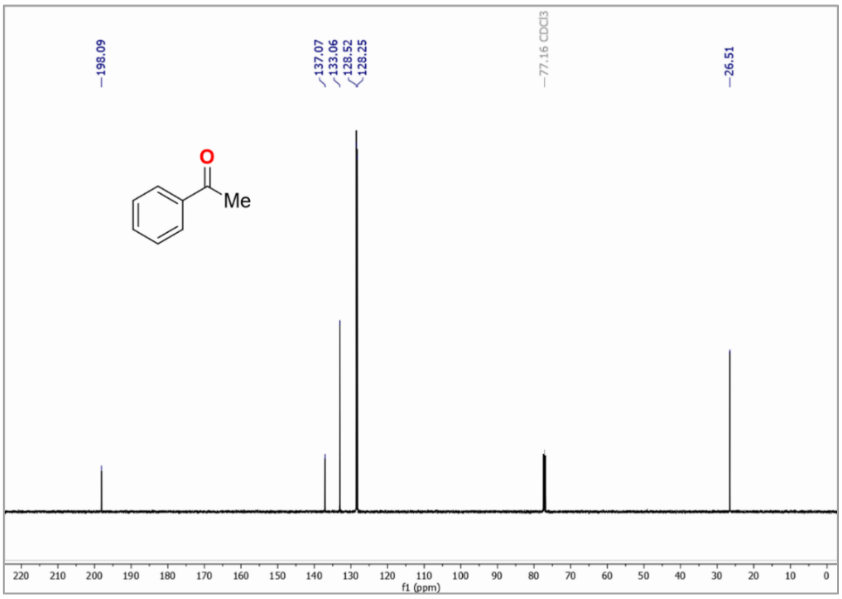

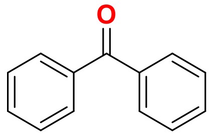

19. *Benzophenone*[16] (Table 2, entry 19): (0.3 mmol scale) Purified by column chromatography using 19:1 hexane:EtOAc. Isolated yield: 92%, 50 mg. TLC R_f = 0.7 (4:1 hexane:EtOAc). ^{1}H NMR (500 MHz, $CDCl_3$) δ 7.81 (d, J = 8.5 Hz, 4H), 7.58 (t, J = 7.4 Hz, 2H), 7.48 (t, J = 7.7 Hz, 4H). ^{13}C NMR (126 MHz, $CDCl_3$) δ 196.81, 137.68, 132.49, 130.13, 128.36. GC-MS (ESI) m/z: M^+ peak: 182.21, base peak: 105.06, tR: 11.3 min.

^{1}H NMR:

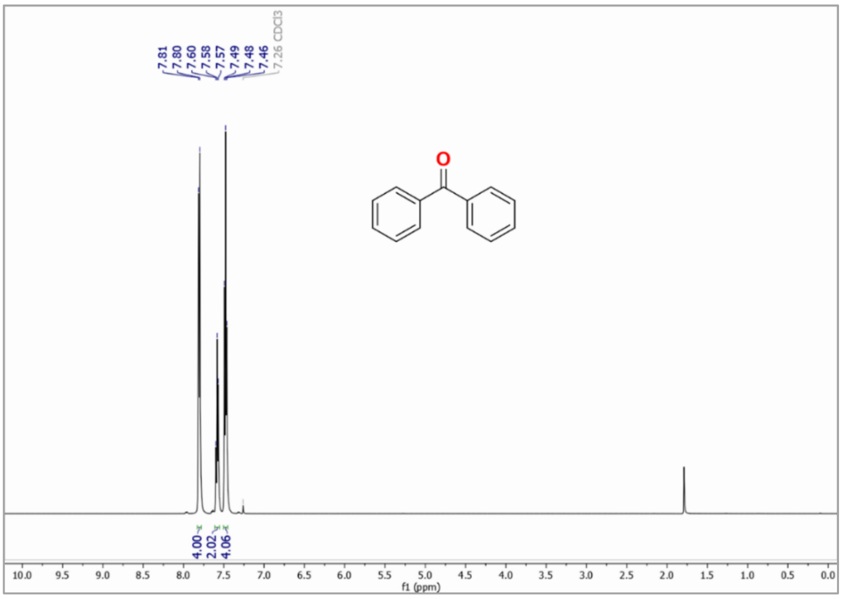

^{13}C NMR:

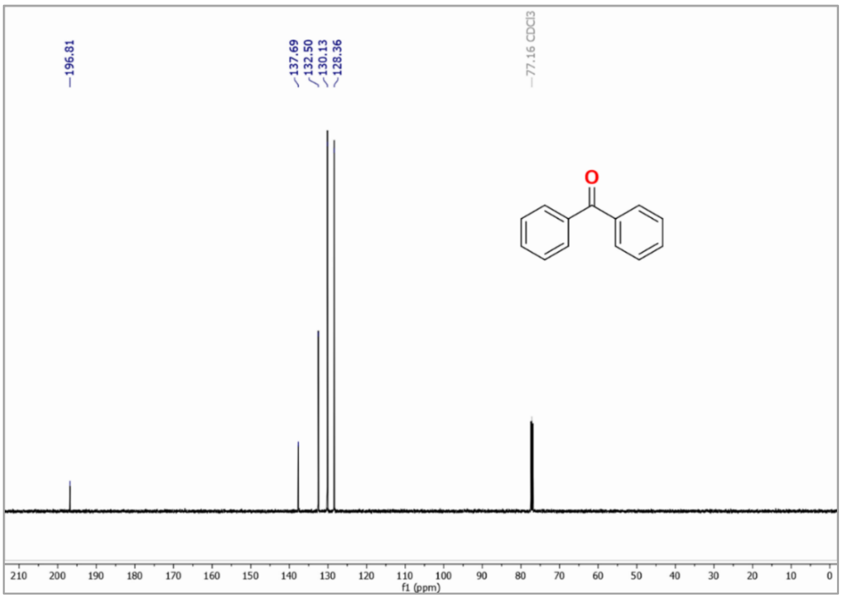

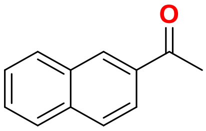

20. *1-(naphthalen-2-yl)ethenone*[16] (Table 2, entry 20): (0.3 mmol scale) Purified by column chromatography using 11.5:1 hexane:EtOAc. Isolated yield: 86%, 44 mg. TLC R_f = 0.3 (8:2 hexane:EtOAc). ^{1}H NMR (500 MHz, $CDCl_3$) δ 8.43 (s, 1H), 8.01 (d, J = 8.6 Hz, 1H), 7.93 (d, J = 8.1 Hz, 1H), 7.85 (dd, J = 8.1, 5.2 Hz, 2H), 7.55 (dt, J = 23.7, 7.1 Hz, 2H), 2.70 (s, 3H). ^{13}C NMR (126 MHz, $CDCl_3$) δ 198.04, 135.61, 134.54, 132.55, 130.20, 129.58, 128.43, 127.80, 126.79, 123.92, 26.67. GC-MS (EI) m/z: M^+ peak: 170.03, base peak: 155.00, tR: 11.011 min.

^{1}H NMR:

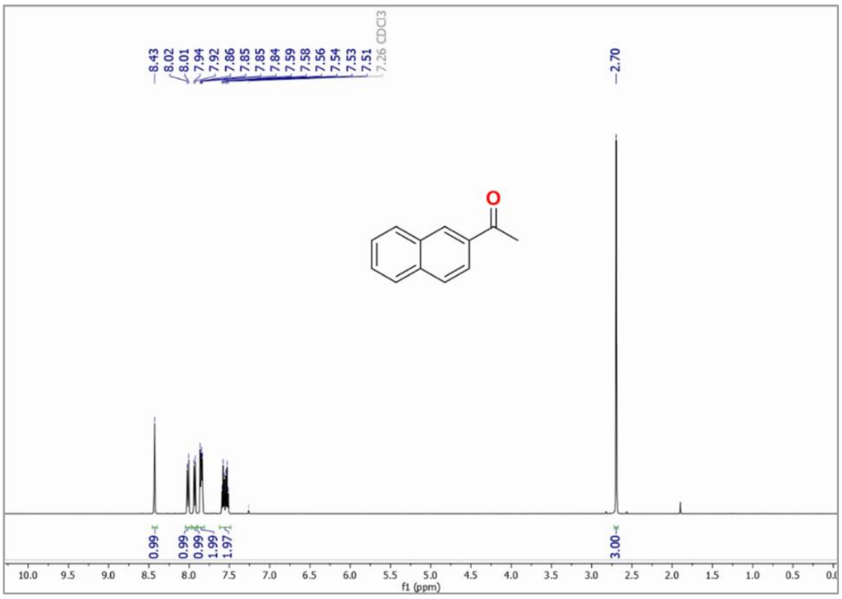

^{13}C NMR:

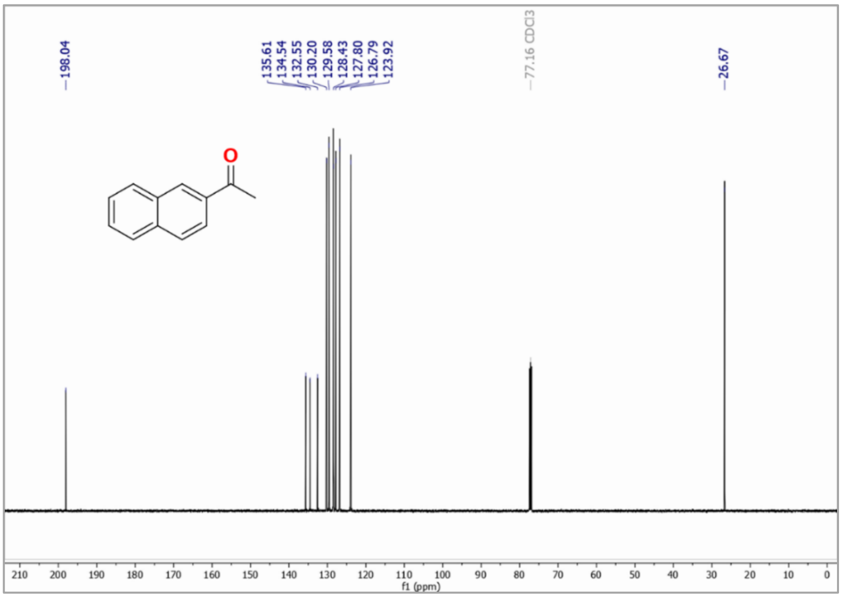

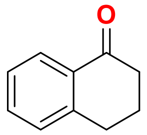

21. *3,4-dihydronaphthalen-1(2H)-one*[16] (Table 2, entry 21): (0.4 mmol scale) Purified by column chromatography using 19:1 hexane:EtOAc. Isolated yield: 91%, 53 mg. TLC R_f = 0.3 (8:2 hexane:EtOAc). ^{1}H NMR (500 MHz, $CDCl_3$) δ 8.02 (d, *J* = 7.8 Hz, 1H), 7.45 (t, *J* = 7.4 Hz, 1H), 7.28 (t, *J* = 7.5 Hz, 1H), 7.23 (d, *J* = 7.6 Hz, 1H), 2.95 (t, *J* = 6.0 Hz, 2H), 2.64 (t, *J* = 6.5 Hz, 2H), 2.12 (p, *J* = 6.2 Hz, 2H). ^{13}C NMR (126 MHz, $CDCl_3$) δ 198.31, 144.51, 133.39, 132.65, 128.80, 127.15, 126.62, 39.18, 29.72, 23.31. GC-MS (EI) m/z: M^+ peak: 146.07, base peak: 118.04, tR: 8.86 min.

^{1}H NMR:

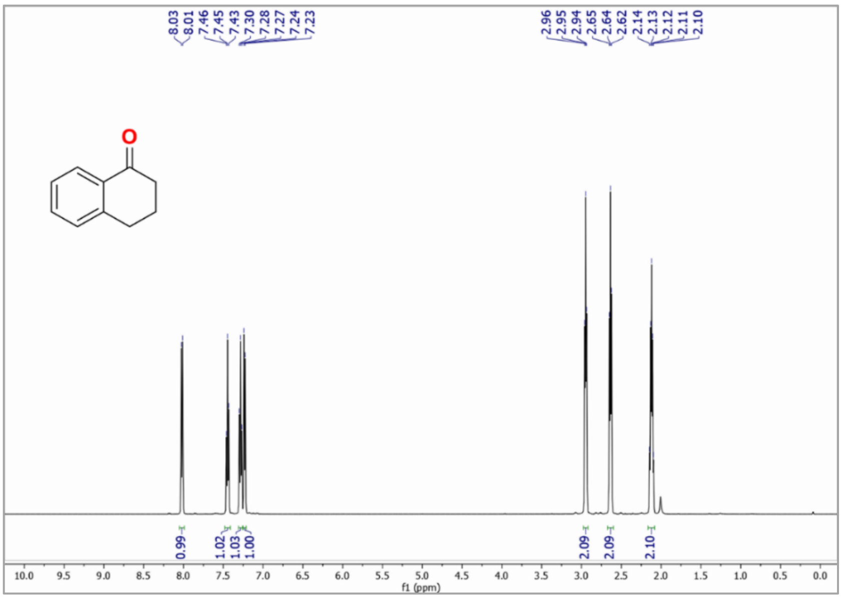

^{13}C NMR:

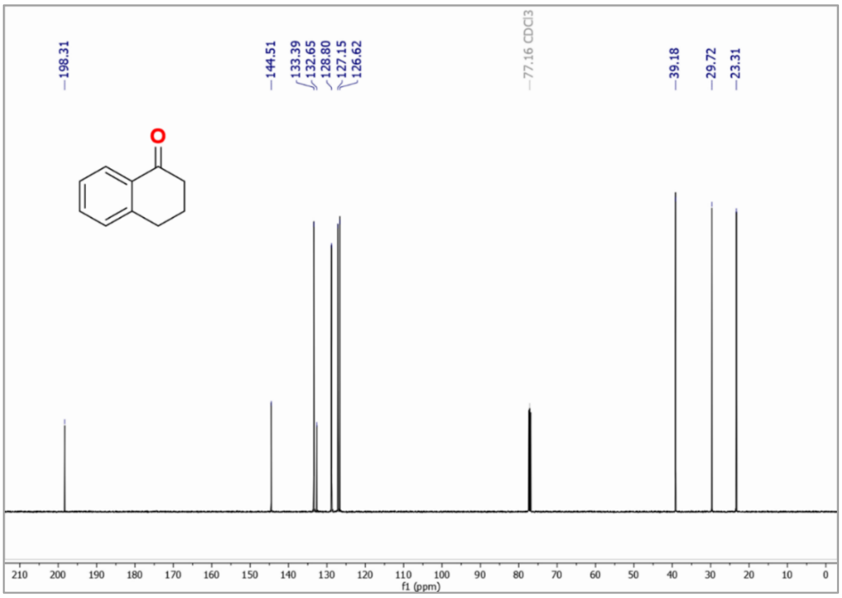

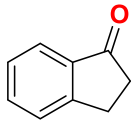

22. *2,3-dihydro-1H-inden-1-one*[16] *(Table 2, entry 22):* (0.4 mmol scale) Purified by column chromatography using 11.5:1 hexane:EtOAc. Isolated yield: 85%, 45 mg. TLC *Rf* = 0.4 (9:1 hexane:EtOAc). ^{1}H NMR (500 MHz, $CDCl_3$) δ 7.74 (d, J = 7.7 Hz, 1H), 7.59 – 7.53 (m, 1H), 7.46 (d, J = 7.7 Hz, 1H), 7.34 (t, J = 7.4 Hz, 1H), 3.15 – 3.09 (m, 2H), 2.69 – 2.63 (m, 2H). ^{13}C NMR (126 MHz, $CDCl_3$) δ 207.09, 155.23, 134.66, 127.35, 126.78, 123.77, 36.29, 25.88. GC-MS (EI) m/z: M^+ peak: 132.10, base peak: 104.11, tR: 7.97 min.

^{1}H NMR:

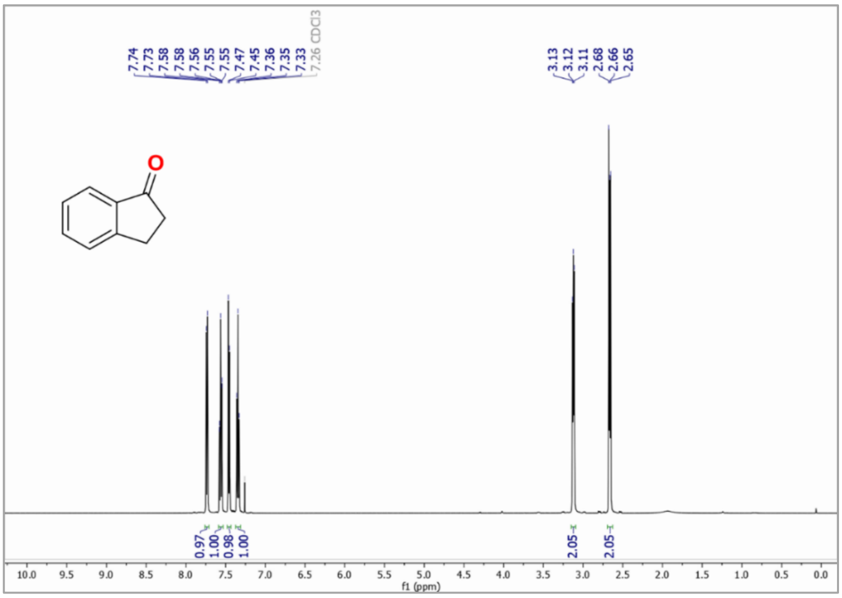

^{13}C NMR:

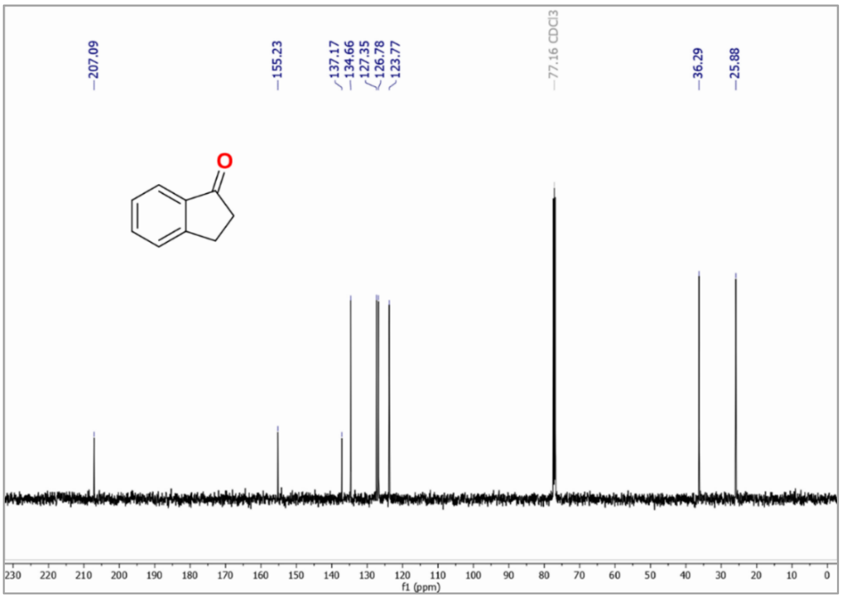

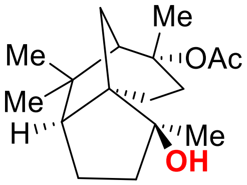

23. *3-hydroxy-3,6,8,8-tetramethyloctahydro-1H-3a,7-methanoazulen-6-yl acetate*[9] *(Table 2, entry 23):* (0.3 mmol scale) Purified by column chromatography using 7:1 hexane:Et_2O. Isolated yield: 52%, 44 mg. TLC R_f = 0.7 (7:3 hexane:EtOAc). ^1H NMR (400.13 MHz, $CDCl_3$, δ, ppm) δ 2.44-2.43 (d, 1H, J = 5.49 Hz), 2.10-2.05 (dd, 1H, J = 18.50 Hz), 1.95 (s, 3H), 1.92-1.89 (m, 2H), 1.86-1.81 (m, 2H),1.62-1.58 (m, 2H), 1.55 (s, 3H), 1.52-1.48 (m, 2H), 1.44-1.36 (m, 1H), 1.30-1.25 (m, 2H), 1.17 (s, 3H), 1.15 (s, 3H), 1.02 (s, 3H); ^{13}C NMR (100.61 MHz, $CDCl_3$) δ 170.36, 85.85, 79.37, 57.32, 54.85, 53.69, 44.81, 41.19, 35.67, 33.52, 29.56, 27.91, 26.69, 25.83, 24.16, 22.69, 21.44 ppm; HRMS (ESI) m/z calc'd $C_{17}H_{28}O_3Na$ $[M+Na]^+$: 303.1931, found 303.1929.

^1H NMR:

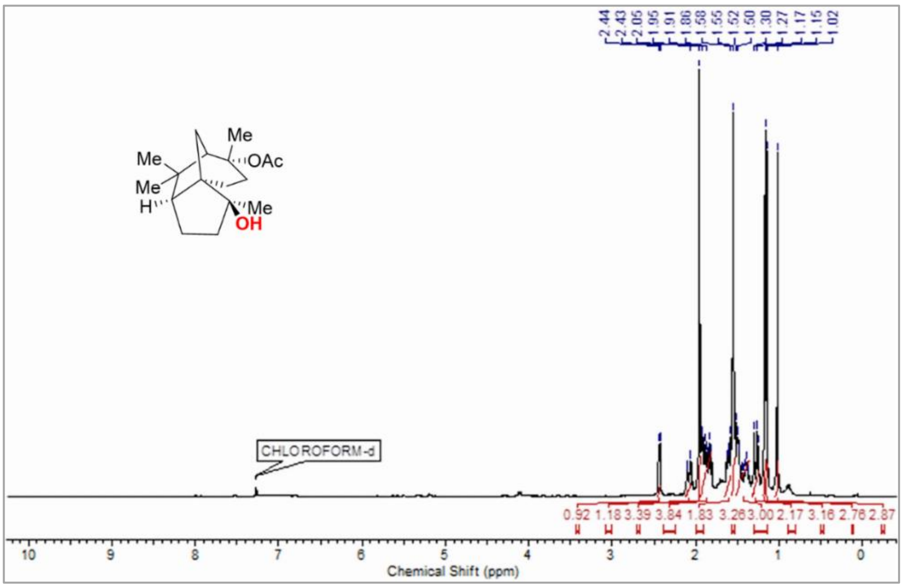

^{13}C NMR:

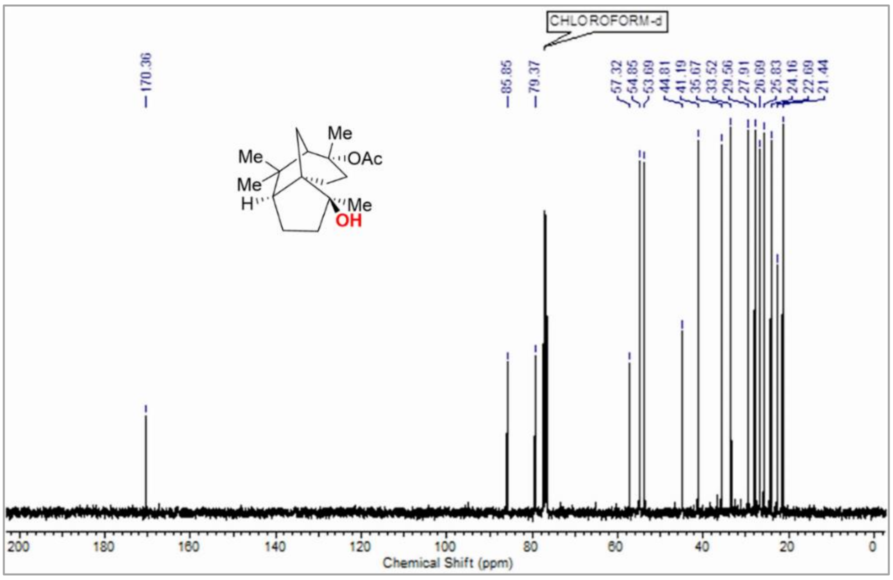

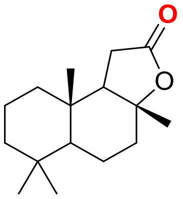

24. *(3aR)-(+)-Sclareolide*[9] (Table 2, entry 24): (0.25 mmol scale) Purified by column chromatography using 19:1 hexane:EtOAc. Isolated yield: 72%, 45 mg. TLC R_f = 0.5 (9:1 hexane:EtOAc). ^{1}H NMR (500 MHz, $CDCl_3$) δ 2.44 – 2.34 (m, 1H), 2.23 (dd, *J* = 16.2, 6.5 Hz, 1H), 2.07 (dt, *J* = 11.9, 3.3 Hz, 1H), 1.96 (dd, *J* = 14.8, 6.5 Hz, 1H), 1.87 (dq, *J* = 14.2, 3.1 Hz, 1H), 1.68 (ddt, *J* = 17.4, 9.9, 4.0 Hz, 2H), 1.49 – 1.34 (m, 4H), 1.33 (d, *J* = 1.0 Hz, 3H), 1.19 (td, *J* = 13.6, 4.4 Hz, 1H), 1.07 (d, *J* = 2.8 Hz, 1H), 1.03 (dd, *J* = 12.9, 3.3 Hz, 1H), 0.91 (s, 3H), 0.88 (s, 3H), 0.83 (s, 3H). ^{13}C NMR (126 MHz, $CDCl_3$) δ 177.01, 86.52, 59.26, 56.80, 42.31, 39.65, 38.86, 36.20, 33.30, 33.26, 28.85, 21.70, 21.05, 20.69, 18.22, 15.20. HRMS (ESI) m/z calc'd $C_{16}H_{26}O_2Na$ $[M+Na]^+$: 273.1825, found 273.1823.

^{1}H NMR:

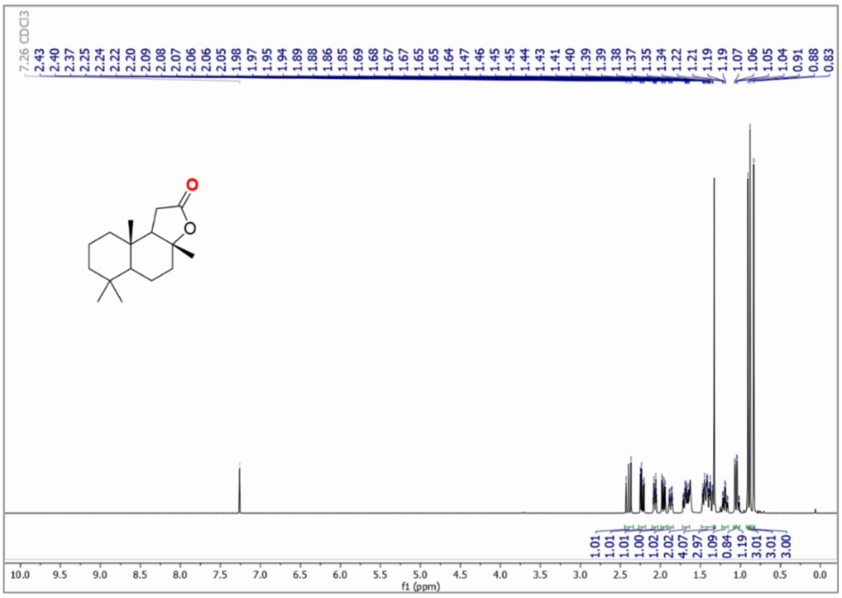

^{13}C NMR:

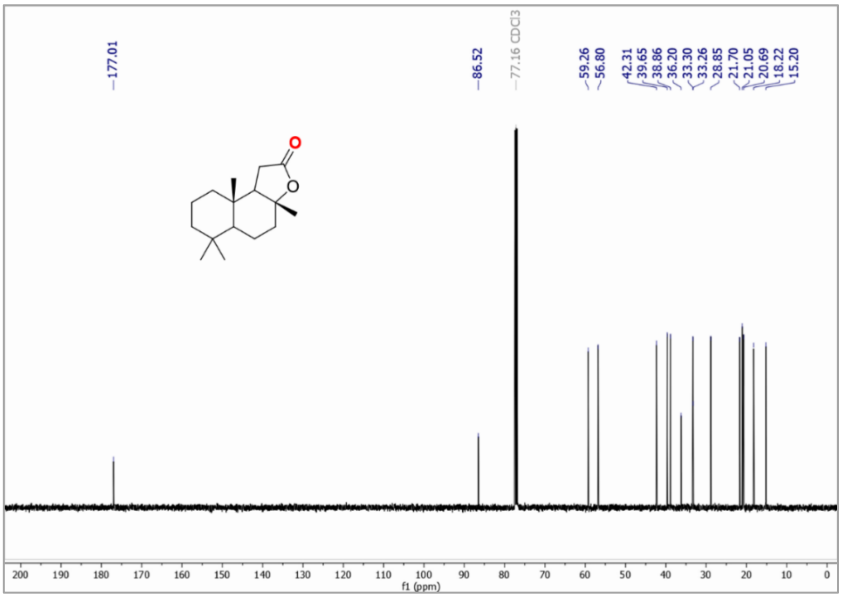

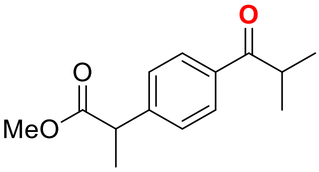

25. *Methyl-2-(4-isobutyrylphenyl)propanoate*[9] (Table 2, entry 25): (0.27 mmol scale) Purified by column chromatography using 6:1 hexane:EtOAc. Isolated yield: 65%, 42 mg. TLC *Rf* = 0.6 (7:3 hexane:EtOAc). ^{1}H NMR (500 MHz, $CDCl_3$) δ 7.92 (d, *J* = 8.4 Hz, 2H), 7.39 (d, *J* = 8.4 Hz, 2H), 3.79 (q, *J* = 7.2 Hz, 1H), 3.67 (s, 3H), 3.53 (hept, *J* = 6.9 Hz, 1H), 1.52 (d, *J* = 7.2 Hz, 3H), 1.21 (d, *J* = 6.9 Hz, 6H). ^{13}C NMR (100.61 MHz, $CDCl_3$) δ 203.97, 174.32, 144.94, 135.14, 128.77, 127.81, 52.22, 45.41, 35.34, 19.14, 18.14 ppm. HRMS (ESI) m/z calc'd $C_{14}H_{19}O_3$ $[M+H]^+$: 235.1329, found 235.1330.

^{1}H NMR:

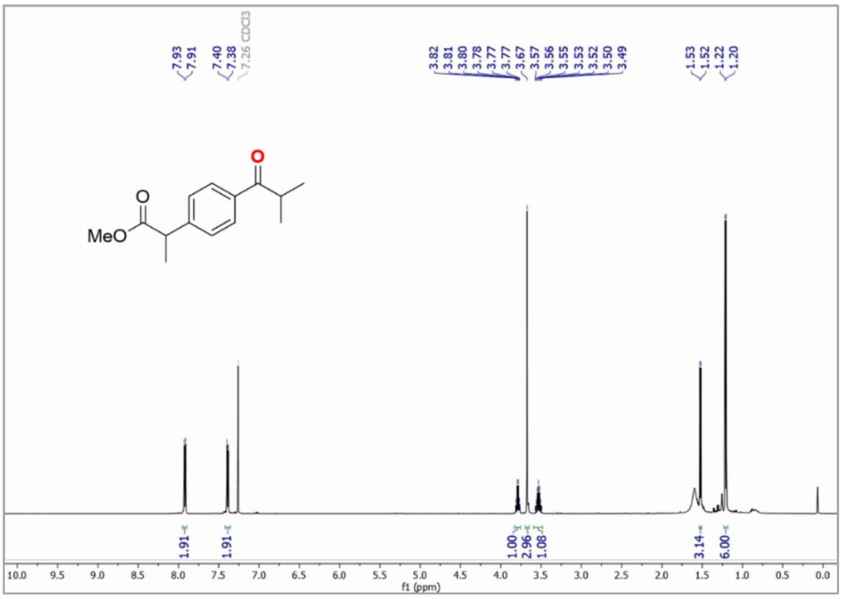

^{13}C NMR:

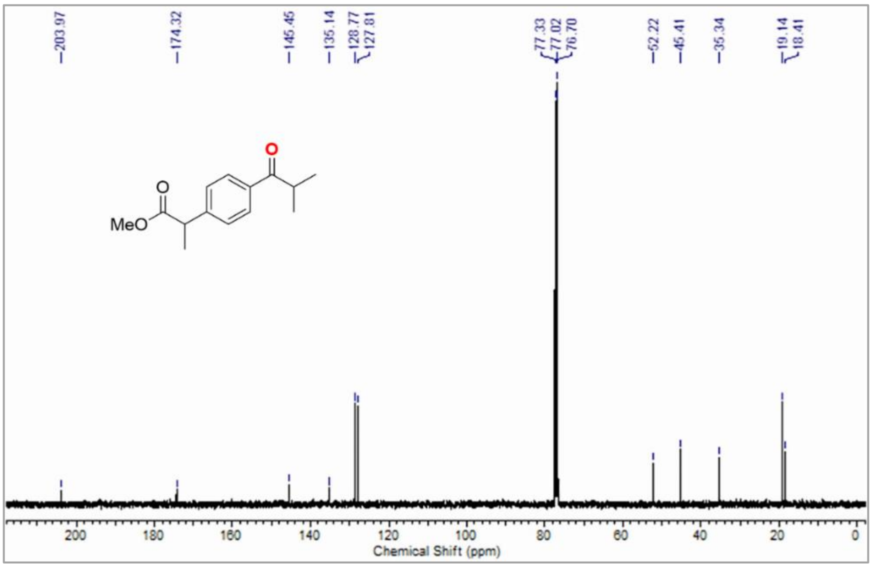

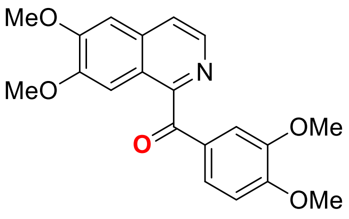

26. *6,7-dimethoxyisoquinolin-1-yl)(3,4-dimethoxyphenyl)methanone*[9] *(Table 2, entry 26)*: (0.2 mmol scale) Purified by column chromatography using 1:1 hexane:EtOAc. Isolated yield: 85%, 60 mg. TLC R_f = 0.5 (3:2 hexane:EtOAc). ^{1}H NMR (400.13 MHz, DMSO-d$_6$) δ 8.41-8.40 (d, 1H, J = 5.38 Hz), 7.87-7.85 (d, 1H, J = 5.38 Hz), 7.58 (s, 1H), 7.50 (s, 1H), 7.30 (s, 1H), 7.26-7.24 (d, 1H, J = 8.31 Hz), 7.04-7.02 (1H, d, J = 8.31 Hz), 3.96 (s, 3H), 3.84 (s, 3H),

3.83 (s, 3H), 3.80 (s, 3H) ppm; ^{13}C NMR (100.61 MHz, DMSO-d$_6$) δ 193.67, 154.05, 153.36, 151.16, 149.13, 140.27, 133.89, 129.58, 127.05, 122.16, 121.05, 111.81, 111.15, 106.02, 103.64, 56.35, 55.98, 55.61 ppm; HRMS (ESI) m/z calc'd $C_{20}H_{20}O_5N$ [M+H]$^+$: 354.1336, found 354.1334.

^{1}H NMR:

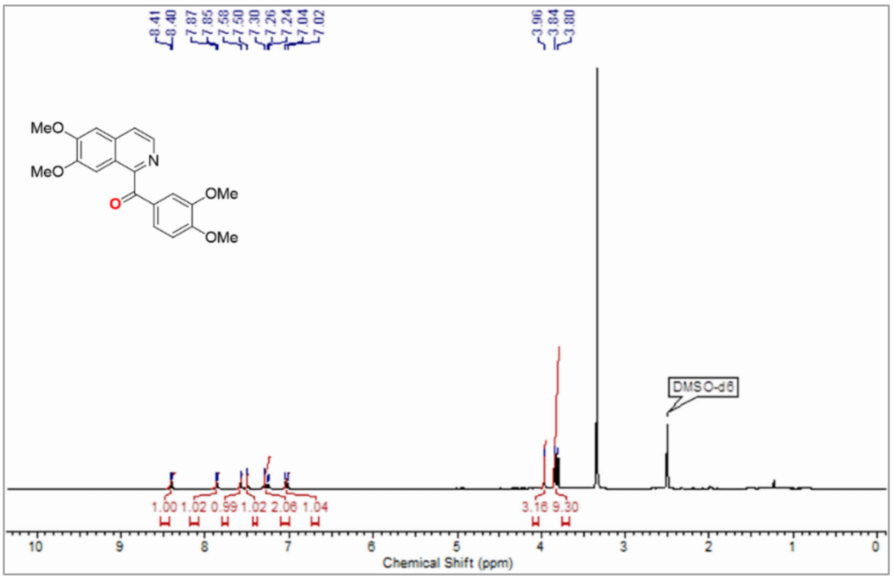

^{13}C NMR:

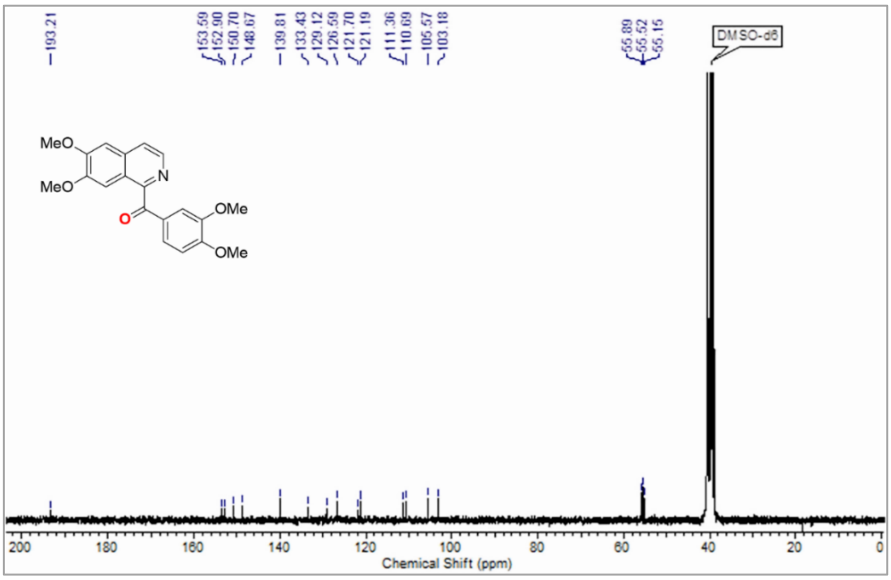

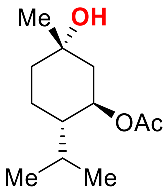

27. *(1R,2S,5S)-5-hydroxy-2-isopropyl-5-methylcyclohexyl acetate*[9] *(Table 2, entry 27a)*: Purified by column chromatography using 8:1 hexane:EtOAc. Overall isolated yield (27a + 27b) (0.4 mmol scale): 62%, 53 mg. TLC R_f = 0.4 (4:1 hexane:EtOAc). ^{1}H NMR (500 MHz, $CDCl_3$) δ 4.98 (td, J = 10.9, 4.6 Hz, 1H), 2.02 (s, 3H), 1.90 – 1.85 (m, 1H), 1.67 (d, J = 13.7 Hz, 1H), 1.53 – 1.47 (m, 2H), 1.39 – 1.31 (m, 3H), 1.31 – 1.27 (m, 1H), 1.25 (s, 1H), 1.23 (s, 3H), 0.91 (d, J = 7.2 Hz, 3H), 0.80 (d, J = 7.0 Hz, 3H). ^{13}C NMR (126 MHz, $CDCl_3$) δ 170.83, 72.09, 71.18, 47.10, 44.73, 37.98, 31.53, 26.48, 21.44, 20.89, 19.50, 16.64. HRMS (ESI) m/z calc'd $C_{12}H_{22}O_3Na$ $[M+Na]^+$: 237.1461, found 237.1460.

^{1}H NMR:

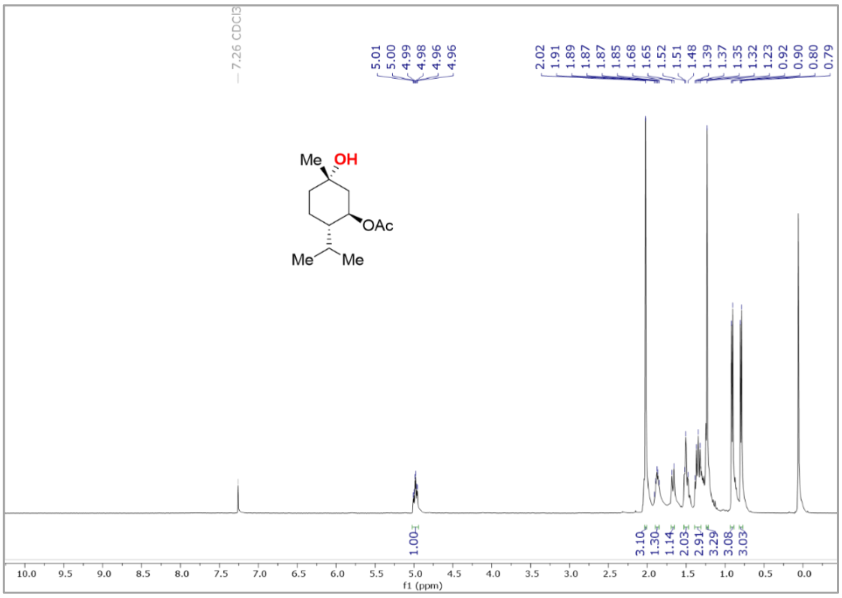

^{13}C NMR:

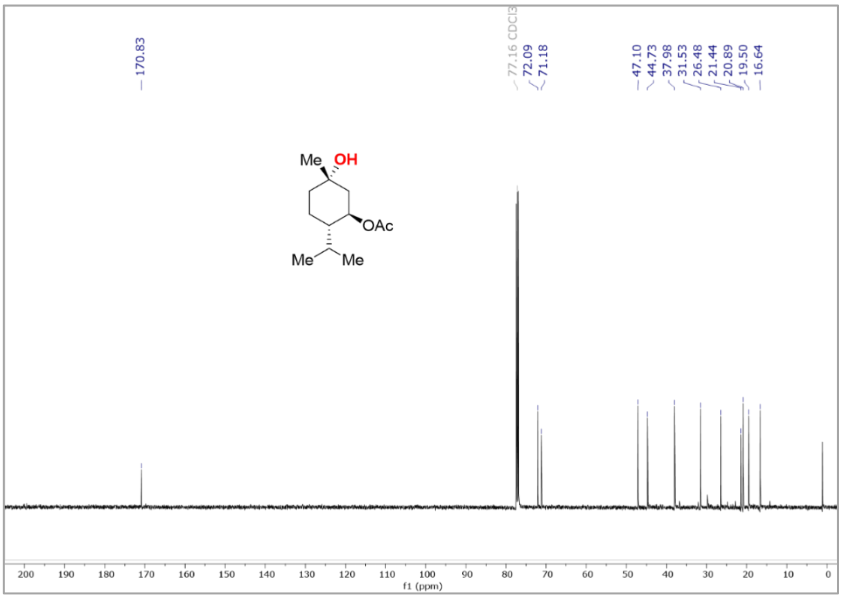

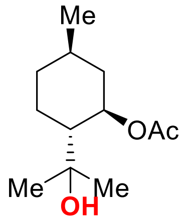

(Table 2, entry 27b) ^{1}H NMR (500 MHz, $CDCl_3$) δ 4.81 (td, *J* = 10.8, 4.2 Hz, 1H), 2.06 (s, 3H), 1.89 (dd, *J* = 13.2, 3.4 Hz, 1H), 1.72 – 1.63 (m, 3H), 1.55 – 1.49 (m, 1H), 1.25 (s, 3H), 1.15 (s, 3H), 1.13 (s, 3H), 1.02 (s, 1H), 0.90 (d, *J* = 6.7 Hz, 3H). ^{13}C NMR (126 MHz, $CDCl_3$) δ 169.93, 76.35, 73.20, 51.73, 41.06, 34.42, 31.44, 28.74, 27.28, 25.81, 21.85.

^{1}H NMR:

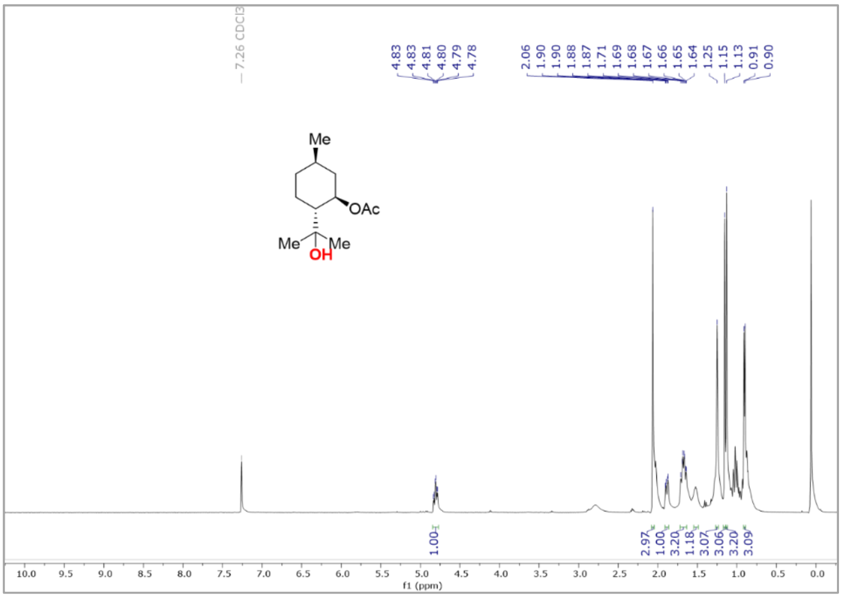

^{13}C NMR:

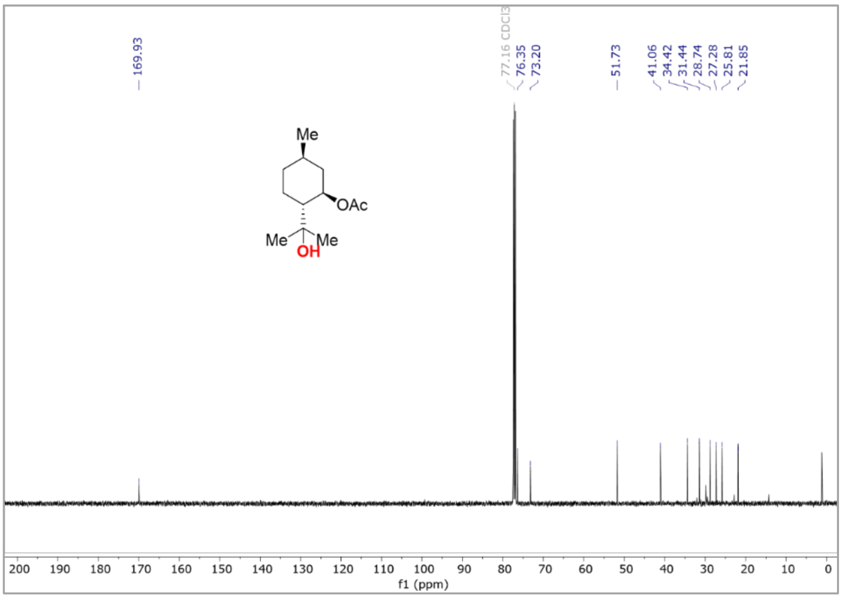

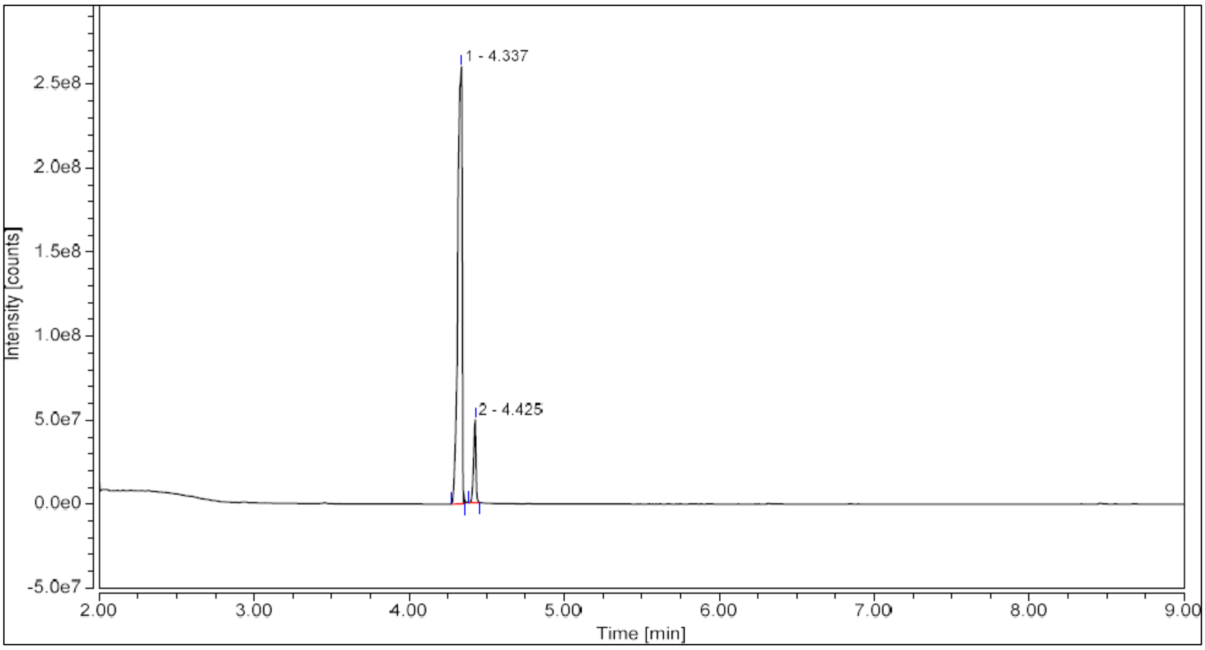

Figure S57. GC-MS chromatogram after reaction with cyclohexane.

4. References

1. W. L. F. Armarego, D. D. Perrin, *Purification of Laboratory Chemicals.* **2009**, 6th edition, Elsevier Science, USA.
2. O. V. Dolomanov, L. J. Bourhis, R. J. Gildea, J. A. K. Howard, H. Puschmann, OLEX2: a complete structure solution, refinement and analysis program. *J. Appl. Crystallogr.* **2009**, *42*, 339-341.
3. G. M. Sheldrick, *Acta Crystallogr., Sect. A: Found. Adv.*, 2015, **71**, 3–8.
4. G. M. Sheldrick, *Acta Crystallogr., Sect. A: Found. Crystallogr.* 2008, **64**, 112– 122.
5. S. Pattanayak, A. J. Jasniewski, A. Rana, A. Draksharapu, K. K. Singh, A. Weitz, M. Hendrich, Que, L., A. Dey, S. Sen Gupta, *Inorg. Chem.* 2017, **56 (11)**, 6352-6361.
6. S.Connolly, A. Fisher, A. Humphries, A. J. Watts, K. E. Wiley, *Preparation and pharmaceutical composition comprising a 4-hydroxy-2-oxo-2,3-dihydro-1,3-benzothiazol-7-yl compound for modulation of beta2-adrenoreceptor activity*, **2009**, World Intellectual Property Organization, WO2009154562 A1.
7. S. Raghavan, J. E. Stelmach, C. J. Smith, H. Li, A. Whitehead, S. T. Waddell, Yi-H. Chen, S. Miao, O. A. Ornoski, J. Garfunkle, X. Liao, J. Chang, X. Han, J. Guo, J. A. Groeper, L. L. Brockunier, K. Rosauer, E. R. Parmee,*Soluble guanylate cyclase activators*, **2011**, WO 2011149921 A1.
8. C. Panda, M. Ghosh, T. Panda, R. Banerjee, S. Sen Gupta, *Chem. Commun.* 2011, **47 (28)**, 8016-8018.
9. S. Jana, M. Ghosh, M. Ambule, S. Sen Gupta, *Org. Lett.* 2017, **19 (4)**, 746-749.
10. S. Hong, Y.-M. Lee, K.-B. Cho, K. Sundaravel, J. Cho, M. J. Kim, W. Shin, W. Nam, *J. Am. Chem. Soc.* 2011, **133 (31)**, 11876-11879.
11. P. L. Hahn, J. M. Lowe, Y. Xu, K. L. Burns, M. K. Hilinski, *ACS Catal.* 2022, **12 (8)**, 4302-4309.
12. B. H. Brodsky, J. Du Bois, *J. Am. Chem. Soc.* 2005, **127 (44)**, 15391-15393.

13. M. Milan, G. Carboni, M. Salamone, M. Costas, M. Bietti, *ACS Catal.* 2017, ***7*** **(9)**, 5903-5911.
14. G.-X. Li, C. A. Morales-Rivera, F. Gao, Y. Wang, G. He, P. Liu, G. Chen, *Chem. Sci.* 2017, **8** **(10)**, 7180-7185.
15. V. Dantignana, M. Milan, O. Cussó, A.Company, M. Bietti, M. Costas, *ACS Cent. Sci.* 2017, **3** **(12)**, 1350-1358.
16. J. A. Marko, A. Durgham, S. L. Bretz, W. Liu, *Chem. Commun.* 2019, **55 (7)**, 937-940.
17. D. F. Evans, D. A. Jakubovic, *J. Chem. Soc., Dalton Trans.* 1988, 2927-2933.
